# Clinical significance of FBXO43 in hepatocellular carcinoma and its impact on tumor cell proliferation, migration and invasion

**DOI:** 10.7717/peerj.15373

**Published:** 2023-05-22

**Authors:** Rulan Ma, Wenbo Liu, Tuanhe Sun, Chengxue Dang, Kang Li

**Affiliations:** 1 Department of Surgical Oncology, The First Affiliated Hospital of Xi’an Jiaotong University, Xi’an, Shaanxi, China; 2 Department of Plastic and Cosmetic Maxillofacial Surgery, The First Affiliated Hospital of Xi’an Jiaotong University, Xi’an, Shannxi, China

**Keywords:** FBXO43, Hepatocellular carcinoma, Prognosis, Immune infiltration, Tumor growth

## Abstract

**Background:**

The effects of FBXO43 on hepatocellular carcinoma (HCC) and its clinical significance have not yet been determined. This study aims to determine the clinical significance of FBXO43 in HCC and its impact on the biological functions of HCC cells.

**Methods:**

Data from TCGA database were downloaded to investigate the expression of FBXO43 in HCC and its correlation with prognosis and immune infiltration. Immunohistochemical staining images of FBXO43 in HCC were acquired from the HPA website. HCC cells (BEL-7404 and SMMC-7721) were transfected with the lentivirus targeting FBXO43 to decrease FBXO43 expression in HCC cells. Western blotting assay was conducted to evaluate the expression level of FBXO43 protein. MTT assay was used to detect the proliferation of HCC cells. The migration and invasion of HCC cells were investigated by performing scratch wound-healing and Transwell invasion assays, respectively.

**Results:**

In comparison to normal tissues, FBXO43 is overexpressed in HCC tissue, and high FBXO43 expression is linked to late T stage, TNM stage and tumor grade. Elevated FBXO43 expression is a risk factor for HCC. In patients with high FBXO43 expression, the overall survival, disease-specific survival, progression-free survival and disease-free survival are poorer. The proliferation, migration and invasion of HCC cells are significantly attenuated in FBXO43 knockdown cells. Also, TCGA data analysis reveals that FBXO43 exhibits a positive correlation with immunosuppression of HCC.

**Conclusion:**

FBXO43 is overexpressed in HCC, and is linked to late tumor stage, worse prognosis and tumor immunosuppression. FBXO43 knockdown restrains the proliferation, migration and invasion of HCC.

## Introduction

Primary liver cancer is the sixth most common cancer and the third leading cause of tumor death worldwide in 2020, with about 906,000 new cases and 830,000 deaths (Sung et al., 2021). In the USA, it is estimated that there will be 41,210 new cases and 29,380 deaths 2023 (Siegel et al., 2023). Hepatocellular carcinoma (HCC) (accounts for 75%–85%) is the main type of liver cancer (Sung et al., 2021). Although in many high-risk countries, the prevalence and mortality rates of liver cancer have declined since the 1970s (Arnold et al., 2020; Petrick et al., 2020), the survival rate of HCC patients remains unsatisfactory (Siegel et al., 2022). Therefore, there is still a need to explore and develop new therapeutic targets and potential treatment options for HCC.

F-box only protein43 (FBXO43), which is also named as EMI2, plays a crucial role in meiosis of spermatocytes (Fei & Zhou, 2022; Gopinathan et al., 2017; Hansen, Tung & Jackson, 2006; Schmidt et al., 2005; Tung et al., 2005; Wang et al., 2021). However, there is limited research on the role of FBXO43 in tumors. It has been reported that FBXO43 is overexpressed in breast cancer and is associated with poor survival and a high risk of metastasis (Vadhan et al., 2020). Our recent study has demonstrated that FBXO43 knockdown significantly inhibits breast cancer cell growth, and prevents the development of a xenograft breast cancer model (Ma et al., 2021). Mechanistic experiments have revealed that the effect of FBXO43 on breast cancer is induced by its interaction with PCNA (Ma et al., 2021). In liver cancer, Xu et al. (2019) found that 10 genes, including FBXO43, were obviously related to HCC progression and prognosis *via* bioinformatics analysis. However, the impact of FBXO43 in HCC and its clinical significance remains unclear.

Therefore, the aim of this study is to clarify the impact of FBXO43 on HCC, including the relationship between FBXO43 expression and prognosis, the effect on HCC cell proliferation, invasion and migration, and the correlation of FBXO43 with HCC immune infiltration.

## Materials & Methods

### Cell culture

RPMI 1640 medium (Corning, USA, 10-040-CVB), containing 10% inactivated FBS (16000-044; Invitrogen, Waltham, MA, USA) and 1% P/S, was used to culture BEL-7404 and SMMC-7721 cells obtained from GeneChem Corporation (Shanghai, China) The culture medium was changed based on the rate of cell growth. A 37 °C incubator containing 5% CO_2_ was used to culture the cells.

### Download and processing of The Cancer Genome Atlas (TCGA) data and acquisition of FBXO43 immunohistochemical staining results

The UCSC database (https://xenabrowser.net/) was used to download the unified and standardized pan-cancer data set: TCGA TARGET GTEx (PANCAN, *N* = 19,131, *G* = 60,499). Then, the FBXO43 gene (ENSG00000156509) expression data in HCC and prognosis-related data were extracted, and each expression value was converted by Log2 (*x* = 0.001).

The immunohistochemical staining images of FBXO43 in HCC and normal liver tissues were obtained from the Human Protein Atlas website (https://www.proteinatlas.org/).

### Construction of lentivirus and cell transfection

The shRNA targeting FBXO43 (5′-CAA GTT ATC AAC TTA GAA A-3′) was designed, and the negative control shRNA is 5′- TTC TCC GAA CGT GTC ACG T-3′. Single-stranded and double-stranded DNA containing shFBXO43 or shCtrl were synthesized. The lentiviral vector was ligated to the endonuclease site of double-stranded DNA. Then, competent cells (TIANGEN, #CB104-03) were transfected. We next identified, extracted and purified the corrected lentiviral vectors. Subsequently, BEL-7404 and SMMC-7721 cells were transfected with the lentiviral. When the transfection efficiency exceeds 80%, the cells are used for further experiment.

### Western blotting

RIPA lysate (P0013B/; Beyotime, Shanghai, China) was used to lyse the cells and extract proteins. A BCA kit (P0010S; Beyotime, Shanghai, China) was used to measure the protein concentration. 10% SDS-PAGE gel electrophoresis and PVDF membrane (Millipore, IPVH00010) were used to separate and transfer protein samples (about 20 µg), respectively. The PVDF membrane was incubated with skim milk at 25 °C for 1 h, and then incubated with the first antibodies (Anti-GAPDH: 1:2,000, sc-32233, Santa-Cruz; Anti-FBXO43: 1:500, HPA024292, Sigma) at 4 °C for the night. The membrane was washed with TBST solution (with 0.1% TWEEN-20). Then, the secondary antibody (Anti-Rabbit-Ig: 1:10000, sc-2004, Santa Cruz; Anti-mouse IgG: 1:2000, sc-2005, Santa Cruz Biotechnology, Inc., Dallas, TX, USA) was used to incubate the membrane at 25 °C for 2 h. The membrane was washed with TBST solution. Finally, the protein bands were detected by using a ECL-PLUS/Kit (M3121; Thermo Fisher, Waltham, MA, USA) in a gel imaging system.

### MTT proliferation assay

A 96-well plate was used to inoculated the cells (2000 cells/well). From 2 to 6 days after inoculation, 5 mg/mL MTT (20 µL/well) was added and incubated for 4 h. After removing the culture medium, DMSO (100 µL/well) was added. The OD value at the 490 nm was detected by enzyme-labeled instrument after oscillating for 2–5 min.

### Colony formation assay

A 6-well plates was used to incubate the cells (800 cells/well). After 11 days, the cells were washed with PBS and fixed with 4% paraformaldehyde (one mL/well). Then, the cells were stained with Giemsa staining (500 µL/ well) for 10-15 min. ddH_2_O was used to wash the cells for 3 times. Finally, the images of the colony were recorded for statistical analysis. The colony formation rate = number of colony / 800 *100%.

### Scratch wound-healing assay

A 6-well plate was used to inoculate the cells (5 ×10^4^ cells/well). The scratches were made on the plate when the cell density exceeds 90%. Then the plate was washed with PBS for 3 times. After 24 and 48 h of culture, the cells were photographed and recorded with a microscope for statistical analysis. Cell migratory rate = (0 h scratch width –24/48 h scratch width)/ 0 h scratch width.

### Transwell invasion assay

Transwell invasion kit (354480; Corning, Corning, NY, USA) was used for cell invasion assay. The serum-free cell suspension of 100uL (containing 1 ×10^5^ cells) was inoculated into Transwell chamber, and the medium containing 30%FBS (600 µL/well) was added to the 24-well plate. After 48 h, the unmigrated cells above the chamber were erased. 4% paraformaldehyde and Giemsa staining were used to fix and stain the chamber, respectively. Then, the cells were photographed and recorded with a microscope for statistical analysis.

### Statistical analysis

The data analysis was performed by using R 3.6.4 (R Core Team, 2020) and GraphPad Prism 8.3.0 software (GraphPad, San Diego, CA, USA). Continuous data is expressed as mean ± standard deviation. Unpaired Student-t test and repeated measurement analysis of variance were conducted to analyze the differences between two groups. The optimal cut-off value was determined by R software package maxstat, HCC patients were divided into high FBXO43 expression group and low FBXO43 expression group according to the optimal cut-off value. Survfit function of R software package survival and Log-rank test were used to perform the prognostic analysis. Besides, we mapped the extracted gene expression profile of HCC to GeneSymbol, and the StromalScore, ImmuneScore and ESTIMATEscore of patients with HCC was calculated *via* using R software package ESTIMATE (version 1.0.1) (Yoshihara et al., 2013). In addition, the infiltration abundance of each immunocyte in tumor tissue of HCC patients was evaluated *via* the R software package IOBR (version 0.99.9; https://github.com/IOBR/IOBR). In addition, the association of FBXO43 expression and the immune infiltrating score and the abundance of infiltrating immunocytes was calculated by using the corr.test function of R software package psych (version 2.1.6; Revelle, 2021). The TIMER2.0 website (http://timer.comp-genomics.org/) was used to evaluate the role of FBXO43 expression and the abundance of tumor-infiltrating immunocytes in HCC prognosis. *P* < 0.05 was considered to be statistically significant.

## Results

### FBXO43 was up-regulated in HCC and was related to worse survival of the patients

**Figure 1:**
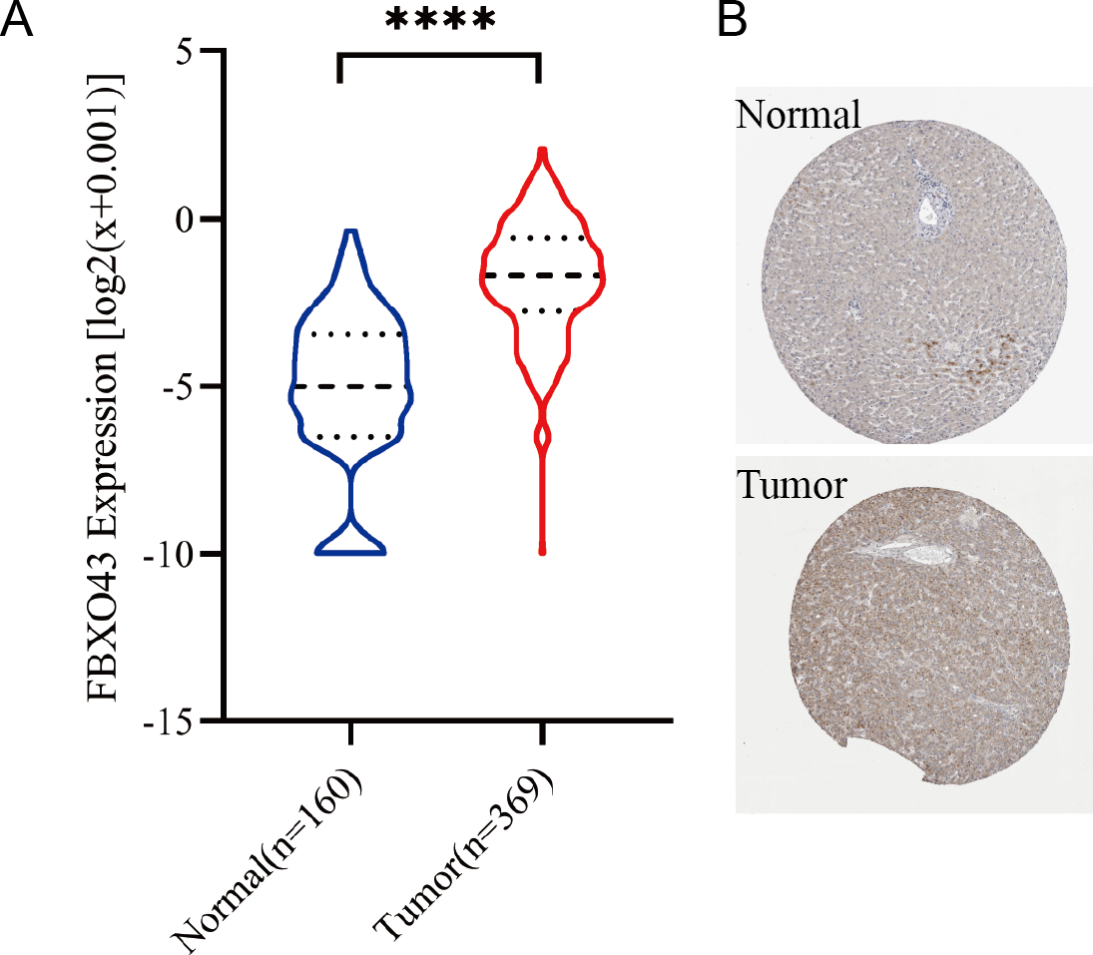
The expression of FBXO43 in HCC. (A) The difference of FBXO43 expression between HCC and normal tissues was analyzed by using TCGA data. (B) The results of immunohistochemical staining of FBXO43 in tumor tissues and normal tissues of HCC from the Human Protein Atlas database. HCC, hepatocellular carcinoma; TCGA, The Cancer Genome Atlas. Unpaired Student-t test was used to analyze the difference between two groups. **** *P* < 0.0001.

**Figure 2:**
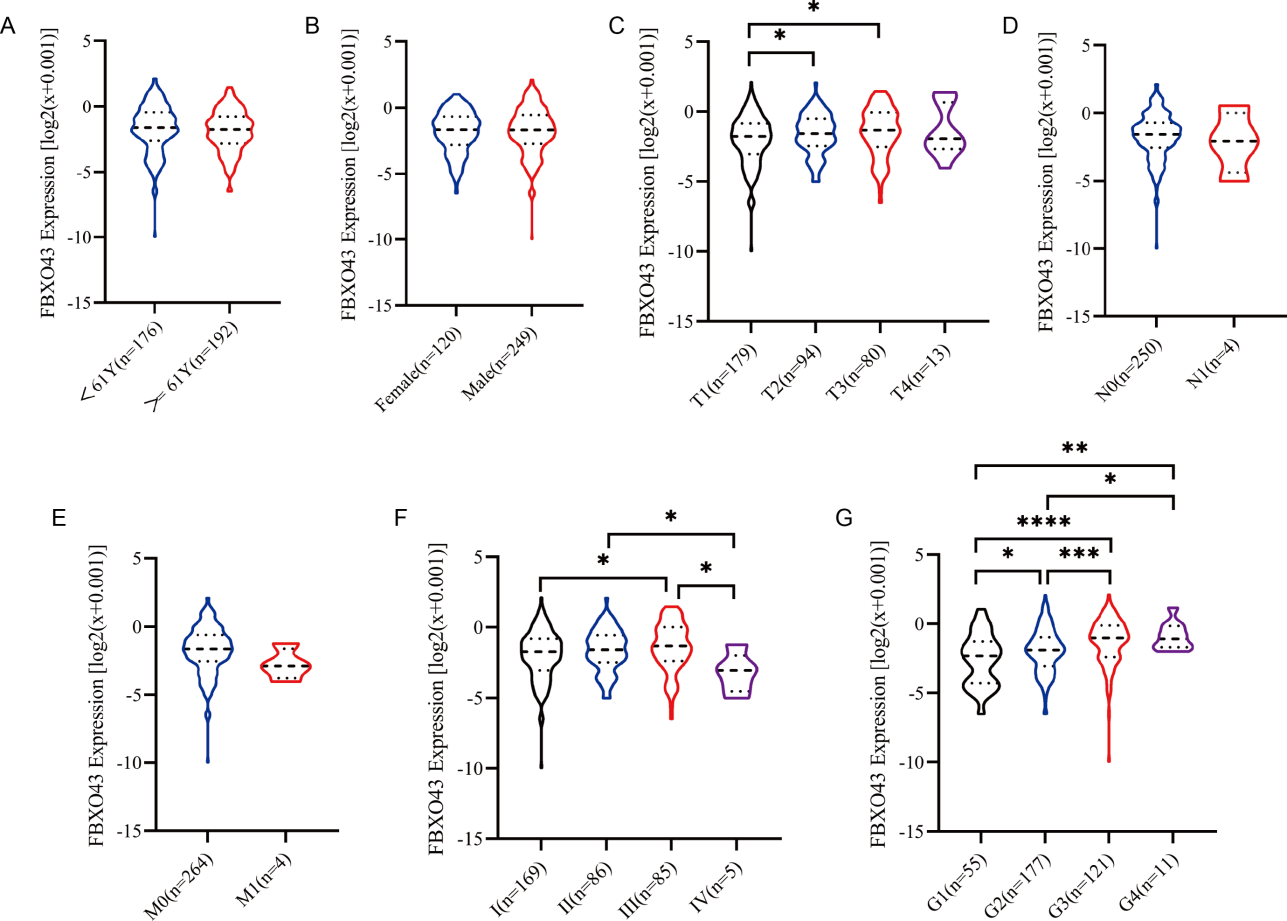
The relationship between FBXO43 expression and the clinicopathological characteristics in HCC. (A–G) The association of the expression level of FBXO43 and the age (A), gender (B), T stage (C), N stage (D), M stage (E), TNM stage (F) and grade (G) of patients with HCC. HCC, hepatocellular carcinoma. Unpaired Student-t test was used to analyze the difference between two groups. * *P* < 0.05; ** *P* < 0.01; *** *P* < 0.001; **** *P* < 0.0001.

**Figure 3:**
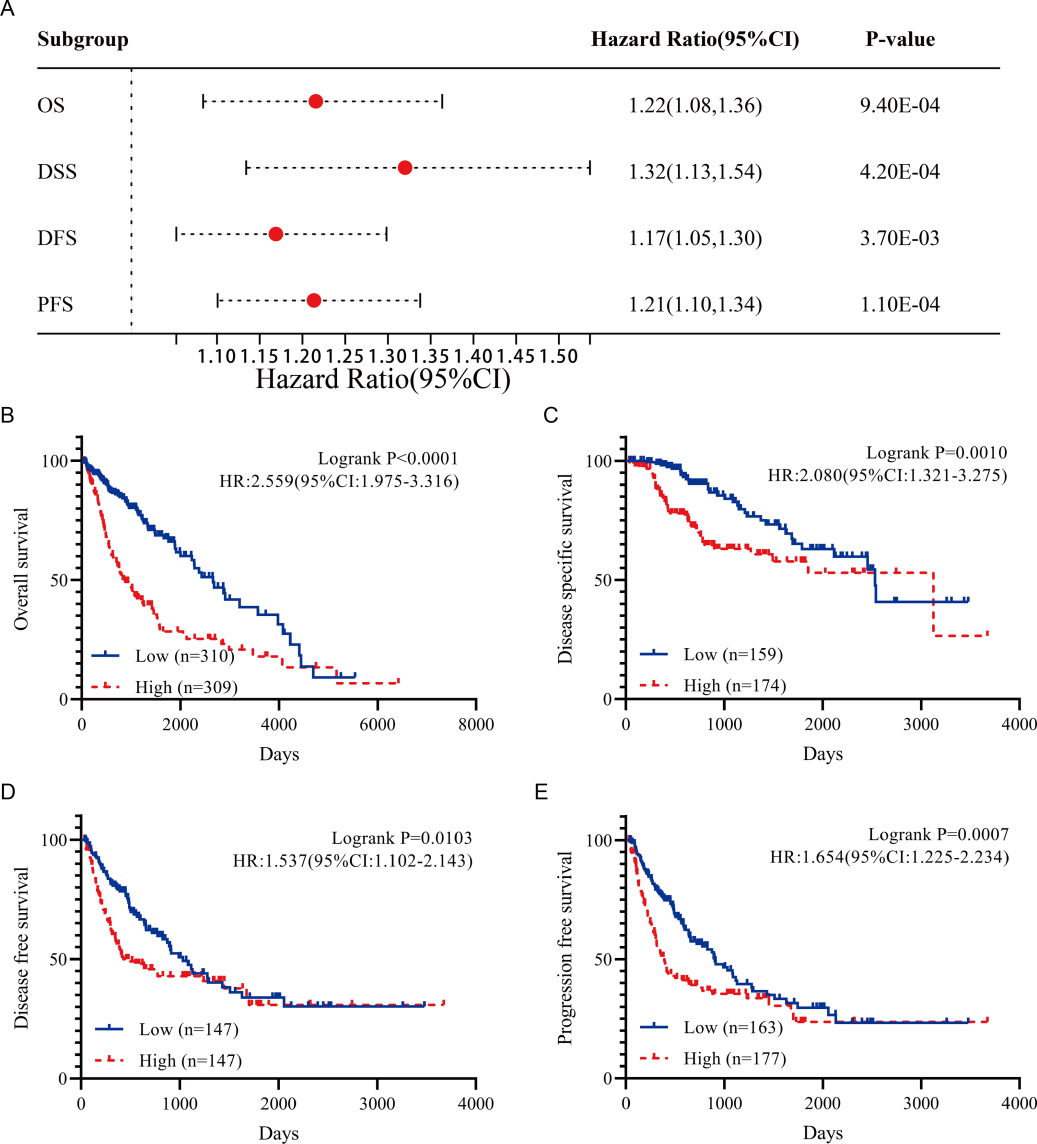
The correlation of FBXO43 expression with the prognosis of HCC patients. (A) Forest plot of the prognosis of HCC patients with high expression of FBXO43. (B) Difference of overall survival of HCC patients with low and high FBXO43 expression level. (C) Difference of disease-specific survival of HCC patients with low and high FBXO43 expression level. (D) Difference of disease-free survival of HCC patients with low and high FBXO43 expression level. (E) Difference of progression-free survival of HCC patients with low and high FBXO43 expression level. HCC, hepatocellular carcinoma. Survfit function of R software package survival and Log-rank test were used to perform the prognostic analysis.

Analysis of HCC data in TCGA database revealed overexpression of FBXO43 in tumor tissues compared to normal tissues (Fig. 1A). The results of immunohistochemical staining from the Human Protein Atlas database also verified that FBXO43 was up-regulated in HCC (Fig. 1B). Further, we investigated the relationship of FBXO43 expression and the clinicopathological features in HCC. The results showed that FBXO43 was not related to age, gender, N stage and M stage, but significantly related to tumor grade, T and TNM stage. The expression level of FBXO43 was positively associated with T1-3, stage I-III and tumor grade (Figs. 2A–2G). The reason why the T4 and stage IV showed lower FBXO43 expression level than other earlier tumor stage might be due to the small sample size of the T4 and stage IV of HCC. The forest plot indicated that the FBXO43 up-regulation was a risk factor for HCC prognosis (Fig. 3A). Further exploration of the relationship between FBXO43 and prognosis also showed that overall survival (OS), disease-specific survival (DSS), disease-free survival (DFS) and progression-free survival (PFS) in patients with high FBXO43 expression were poorer than patients with FBXO43down-regulation (Figs. 3B–3E).

### Downregulation of FBXO43 restrained HCC proliferation

**Figure 4:**
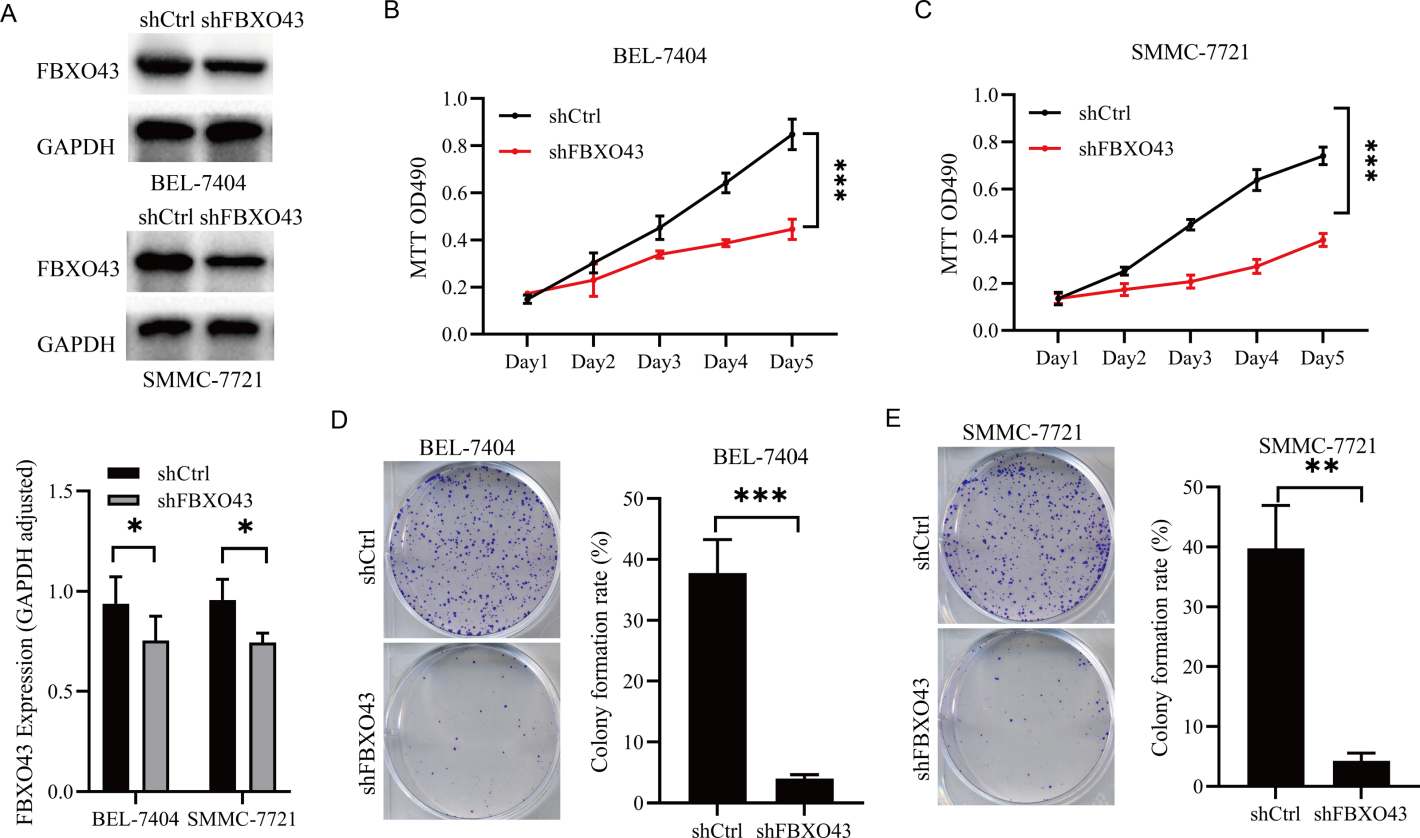
FBXO43 knockdown inhibited the proliferation of HCC cells. (A) Western blotting assay showed that the expression level of FBXO43 was deceased after transfection with lentivirus targeting FBXO43. (B–C) MTT assay showed the inhibition of FBXO43 knockdown on the proliferation of HCC cells. (D–E) Colony formation assay showed the suppression of FBXO43 knockdown on colony formation ability of HCC cells. HCC, hepatocellular carcinoma. The experiments were repeated three times. Unpaired Student-t test and repeated measurement analysis of variance were conducted to analyze the differences between two groups. * *P* < 0.05; ** *P* < 0.01; *** *P* < 0.001.

In order to clarify the role of FBXO43 in HCC, we constructed a lentiviral vector targeting FBXO43 to reduce its expression level. Western blot analysis indicated a significant decrease in the protein expression level of FBXO43 in BEL-7404 and SMMC-7721 cells after knockdown (Fig. 4A). Subsequently, we conducted an MTT proliferation assay, which demonstrated that FBXO43 knockdown significantly inhibited HCC cell proliferation (Figs. 4B–4C). Additionally, the inhibition of FBXO43 knockdown on the colony formation ability of HCC was confirmed through a colony formation assay (Figs. 4D–4E).

### Suppression of FBXO43 inhibited HCC migration and invasion

**Figure 5:**
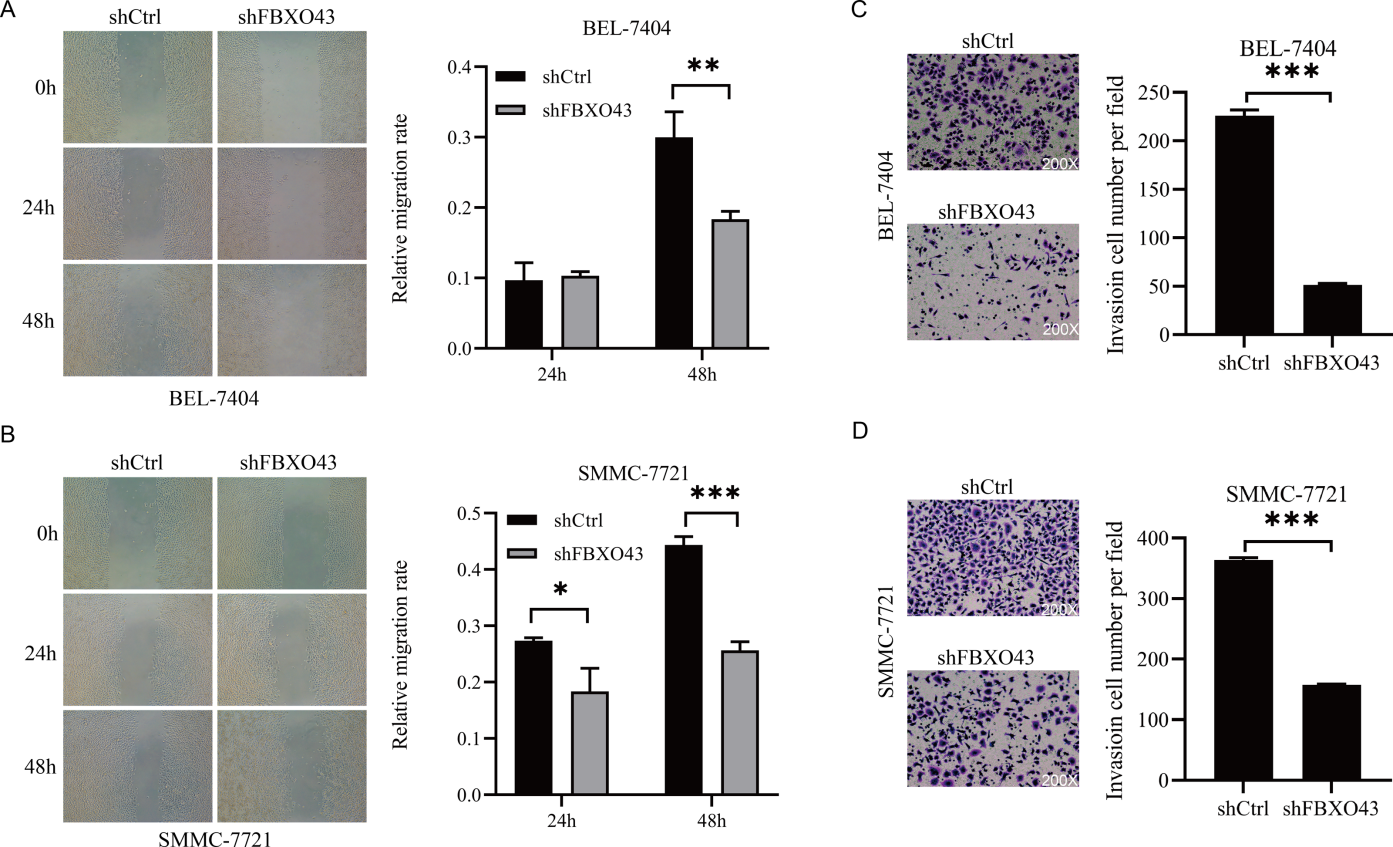
The impact of FBXO43 knockdown on the migration and invasion of HCC cells. (A–B) Scratch wound-healing assay showed the inhibition of FBXO43 knockdown on the migration of HCC cells. (C–D) Transwell invasion assay showed the inhibition of FBXO43 knockdown on the invasion of HCC cells. HCC, hepatocellular carcinoma. The experiments were repeated three times. Unpaired Student-t test was used to analyze the difference between two groups. * *P* < 0.05; ** *P* < 0.01; *** *P* < 0.001.

Given that FBXO43 knockdown affected HCC cell proliferation, we further verified its impact on HCC migration and invasion. It was found that FBXO43 suppression significantly inhibited the migratory (Figs. 5A–5B) and invasive ability (Figs. 5C–5D) of HCC cells.

### FBXO43 was related to immunosuppression of HCC

**Figure 6:**
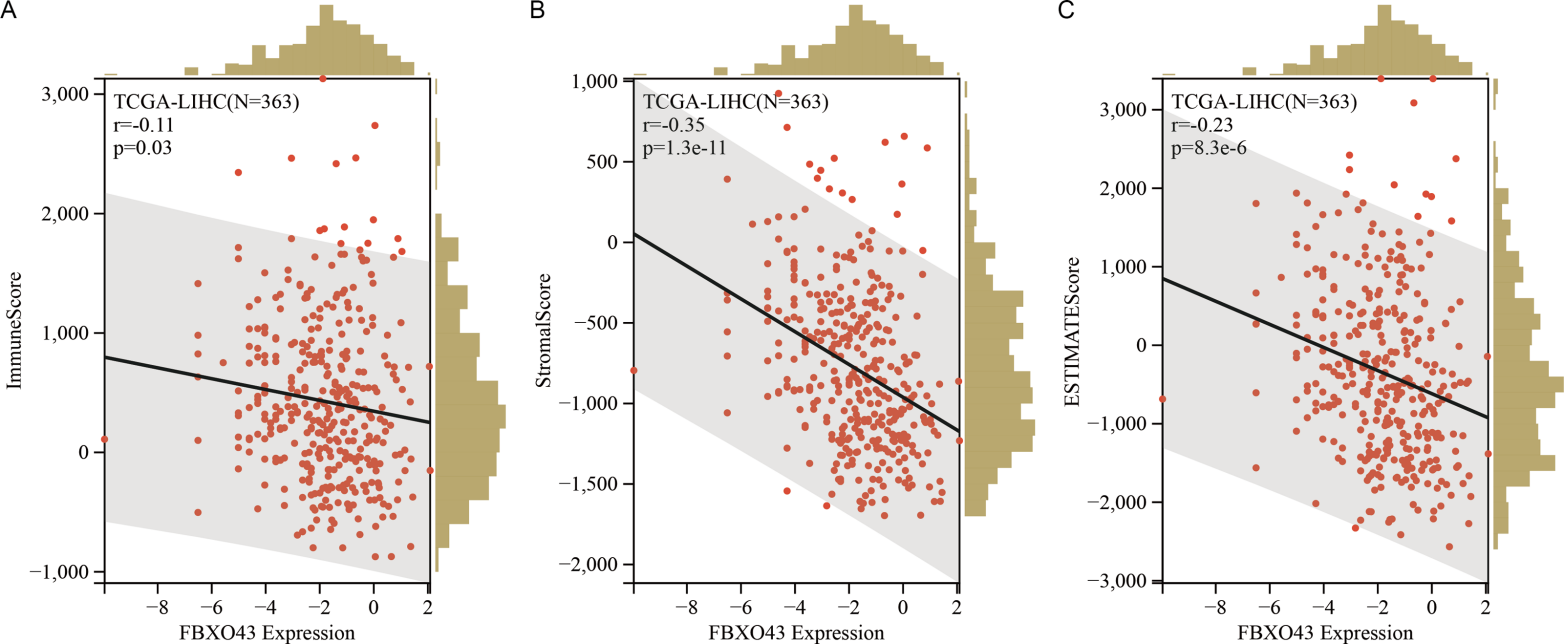
Correlation analysis of FBXO43 expression with immune infiltrating score. (A–C) The expression level of FBXO43 was negatively correlated with ImmuneScore (A), SromalScore (B) and ESTIMATEScore (C) in HCC. HCC, hepatocellular carcinoma. The association of FBXO43 expression and the immune infiltrating score was calculated by using the corr.test function of R software package psych (version 2.1.6; Revelle, 2021).

**Figure 7:**
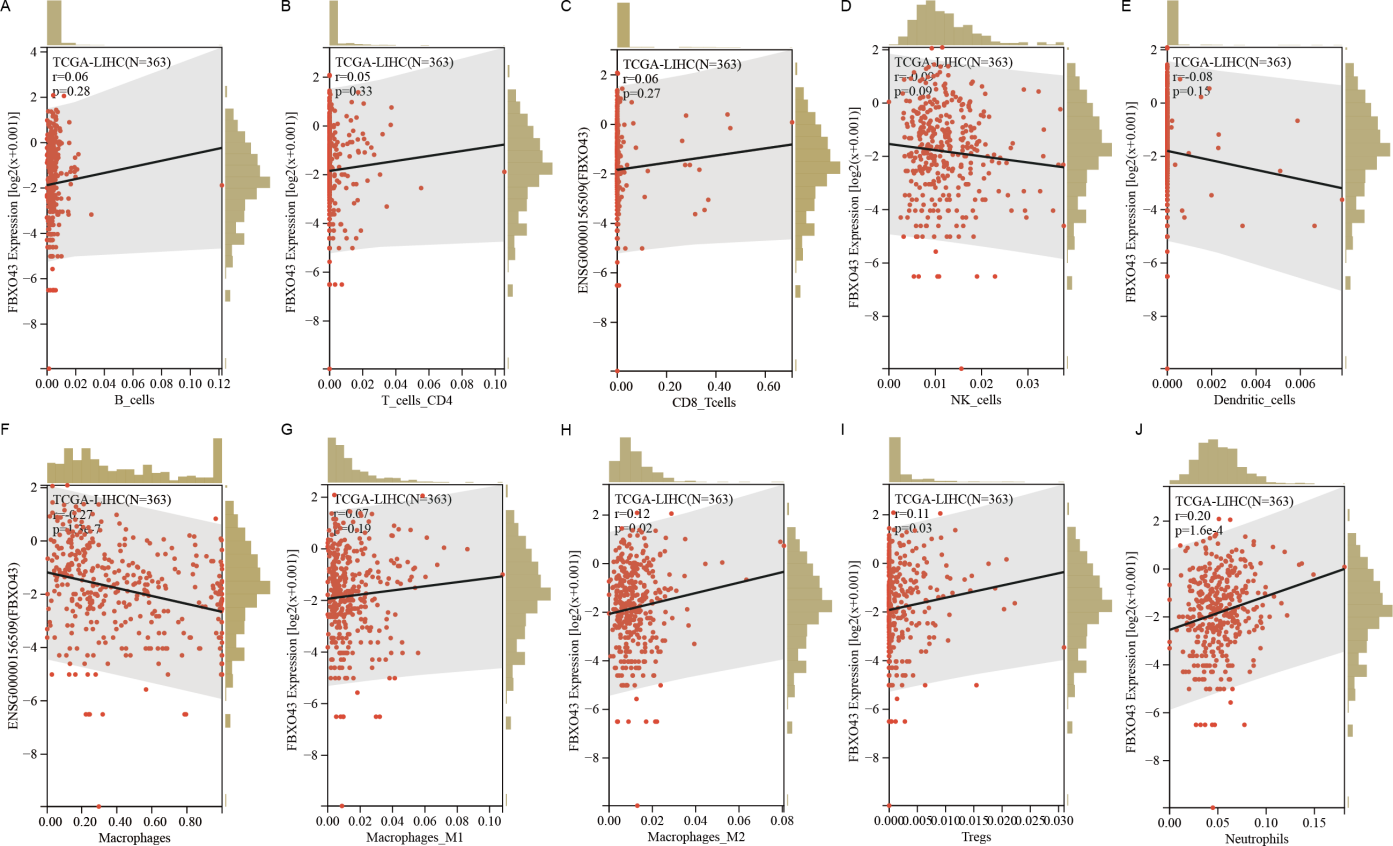
Correlation analysis of FBXO43 expression with the relative abundance of tumor infiltrating immunocytes. (A–J) The correlation between FBXO43 expression level and the tumor infiltrating degree of B cells (A), CD4 T cells (B), CD8 T cells (C), NK cells (D), DCs (E), macrophages (F), M1 macrophages (G), M2 macrophages (H), Tregs (I) and neutrophils (J). HCC, hepatocellular carcinoma; NK cells, natural killer cells; DCs, dendritic cells; Tregs, T regulatory cells. The association of FBXO43 expression and the abundance of infiltrating immunocytes was calculated by using the corr.test function of R software package psych (version 2.1.6; Revelle, 2021).

**Figure 8:**
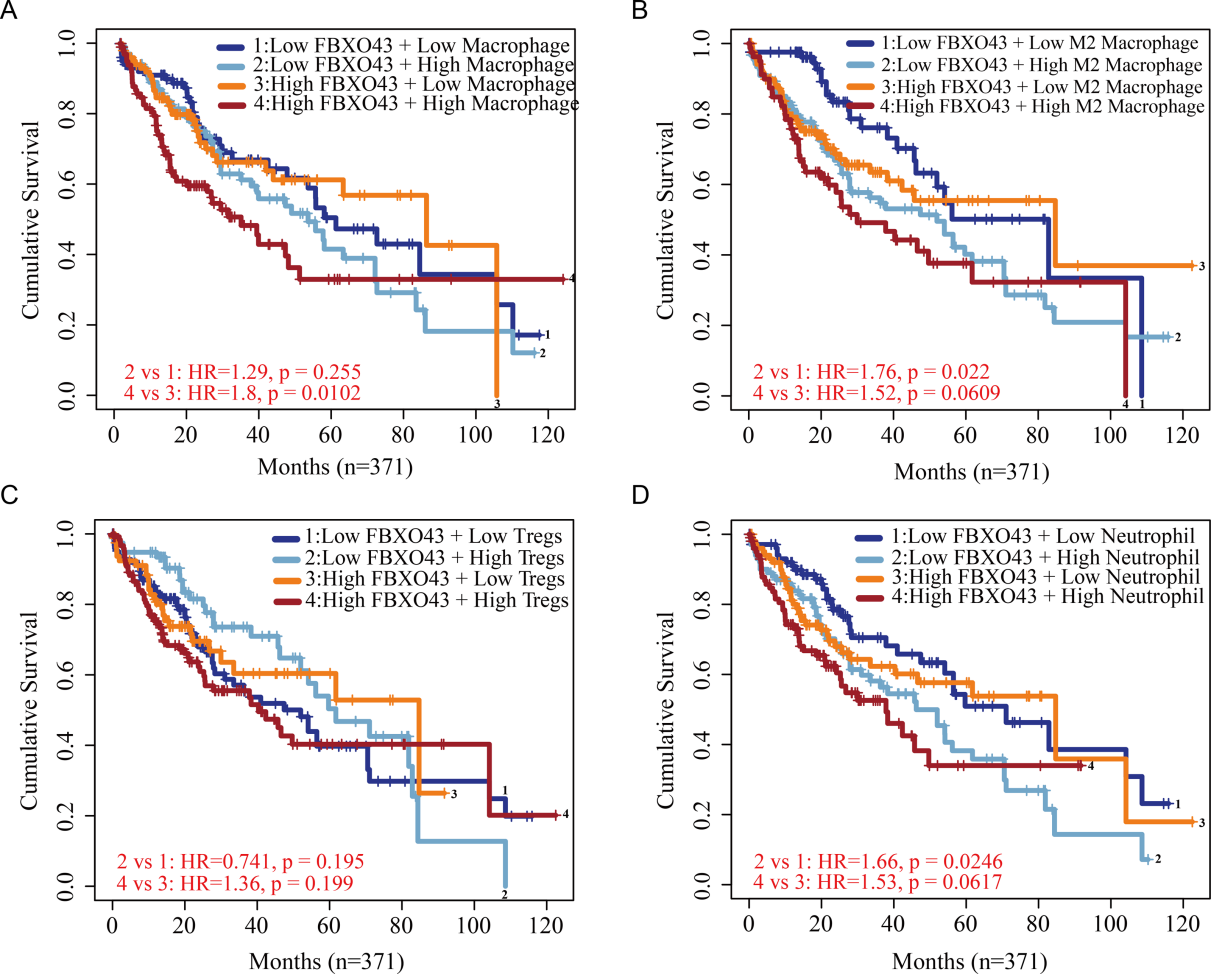
The role of FBXO43 expression and the abundance of tumor-infiltrating immunocytes in HCC prognosis. (A–D) The impact of FBXO43 expression and infiltrating abundance of macrophage, M2 macrophage, Tregs and neutrophil of the overall survival of HCC. HCC, hepatocellular carcinoma; Tregs, T regulatory cells. The TIMER2.0 website was used to evaluate the role of FBXO43 expression and the abundance of tumor-infiltrating immunocytes in HCC prognosis.

Through TCGA database analysis, we observed a negative correlation between high expression of FBXO43 and ImmuneScore (Fig. 6A), StromalScore (Fig. 6B) and ESTIMATEScore (Fig. 6C) in patients with HCC. Further investigation of the relative enrichment of infiltrating immunocytes in HCC did not reveal any association between FBXO43 expression and the infiltration of B cells, CD4 T cells, CD8 T cells, natural killer (NK) cells or dendritic cells (DCs) (Figs. 7A–7E). However, FBXO43 expression was positively related to the relative abundance of immunosuppression-related cells, including M2 macrophages, T regulatory cells (Tregs) and neutrophils (Figs. 7F–7J). The higher the level of FBXO43 expression, the lower the degree of macrophage infiltration (M1 macrophages showed no significant change, but M2 macrophage infiltration level was higher) (Figs. 7F–7H). Moreover, as the expression of FBXO43 increased in HCC, more Tregs and neutrophils were found to infiltrate the tumor tissue (Figs. 7I–7J). The impact of FBXO43 expression and infiltration of macrophages, M2 macrophages, Tregs and neutrophils on HCC prognosis was also investigated. It was showed that when FBXO43 was overexpressed with high infiltrations of macrophages (Fig. 8A), M2 macrophages (Fig. 8B), Tregs (Fig. 8C) and neutrophils (Fig. 8D), patients with HCC had the worst OS. Taken together, FBXO43 overexpression in HCC was associated with an immunosuppressive status and worse prognosis.

## Discussion

Globally, HCC is one of the most common primary malignant tumors (Sung et al., 2021). Although the survival rate of patients with HCC has significantly improved in the past 50 years, most patients are diagnosed at an advanced stage (Miller et al., 2022; Siegel et al., 2022). Thus, it is still necessary to find new promising targets for diagnosing and treating HCC. Previous bioinformatic analysis of HCC showed that 10 genes, including FBXO43, were linked to HCC progression and prognosis (Xu et al., 2019). However, the expression level of FBXO43 in HCC and its clinical significance, as well as its effect on HCC cells and its relationship with HCC immune infiltration, are still unclear. Hence, this study verified the effect of FBXO43 in HCC through TCGA database analysis and cytological experiments.

Our previous study on breast cancer suggests that FBXO43 is overexpressed in breast cancer and is significantly related to a worse prognosis (Ma et al., 2021). Knockdown of FBXO43 can significantly inhibit the development of breast cancer *in vivo* and in vitro (Ma et al., 2021). In this study, we analyzed the TCGA database and found that the expression level of FBXO43 in HCC was significantly higher compared to normal liver tissues. Increased expression of FBXO43 was associated with late stage of HCC patients (including tumor grade, T and TNM stage). Further prognostic analysis indicated that FBXO43 overexpression was a risk factor for HCC prognosis. The higher the expression level of FBXO43, the worse the OS, DSS, DFS and PFS of HCC patients. These findings are consistent with the results of Xu et al. (2019), indicating that FBXO43 is an important prognostic factor in patients with HCC. In addition, we used targeted lentivirus to transfect HCC cells to explore the role of FBXO43 knockdown on the biological functions of HCC cells. It was found that down-regulation of FBXO43 could inhibit the proliferation, migration and invasion of HCC cells, revealing the pro-tumor effect of FBXO43, consistent with its role in breast cancer (Ma et al., 2021).

The immune system has a critical role in the occurrence and development of tumors. Recently, immunotherapy has become a novel first-line treatment choice for advanced HCC (Hilmi et al., 2019; Laface et al., 2022; Sangro et al., 2021). Studies have shown that infiltrating immunocytes in tumor tissue can interact with tumor cells and affect tumor progression, and can affect the prognosis of patients (Dong et al., 2016; Gryziak et al., 2022; Pham et al., 2022; Sia et al., 2017). In particular, reports indicate that immunosuppressive tumor microenvironment due to tumor immunocyte infiltration can lead to poor prognosis (Hoechst et al., 2008; Khanam & Kottilil, 2022). Tumor-associated macrophages have a critical role in HCC progression. Macrophages can be polarized into two different phenotypes: M1 macrophages and M2 macrophages (Chávez-Galán et al., 2015; Martinez et al., 2008). While M1 macrophages have an anti-tumor effect, M2 macrophages promote tumor growth and are linked to poor prognosis (Mantovani & Sica, 2010; Murray, 2017) (Dong et al., 2016; Sia et al., 2017). Studies show that M2 macrophages can inhibit NK cells and CD8^+^ T cells, accelerating tumor progression (Eggert et al., 2016; Li et al., 2017). They can also produce cytokines to promote HCC cell growth and migration (Yeung et al., 2015). Besides, there have been reports indicating that Tregs inhibits the cytotoxicity of CD8^+^ T cells, resulting in a negative outcome for HCC (Moghaddam et al., 2022; Zheng et al., 2017). An increasing number of studies have shown that neutrophil not only play a crucial role in creating an immunosuppressive environment that supports tumor development, but also act as a driving force in tumor progression (Jiang et al., 2022; Shaul & Fridlender, 2019). It also plays an important driving role in HCC (Geh et al., 2022). In our research, we found that FBXO43 expression was inversely correlated with immune infiltration score (including ImmuneScore, StromalScore and ESTIMATEScore) in HCC, but was positively related to the infiltration of macrophages (especially M2 macrophages), Tregs and neutrophils in HCC. Moreover, patients with high FBXO43 expression and high levels of infiltrating M2 macrophages, Tregs and neutrophils had a worse OS, indicating that FBXO43 exerts a critical role in regulating the recruitment of immune infiltrating cells in HCC. Therefore, it can be speculated that FBXO43 may promote HCC progression and poor prognosis by influencing the infiltration of M2 macrophages, Tregs and neutrophils.

However, this study still has some limitation. Firstly, the role of FBXO43 in HCC was only explored through bioinformatics analysis and cell experiments. The effect of FBXO43 *in vivo* was not investigated. Secondly, the underlying mechanism of FBXO43 in HCC was not explored. Therefore, in the next step, we intend to further clarify the underlying mechanism of FBXO43 in HCC in vivo.

## Conclusions

Our research confirms the overexpression of FBXO43 in HCC and its relationship with immunosuppressive status and poor prognosis. FBXO43 knockdown could inhibit HCC cell proliferation, migration and invasion.

##  Supplemental Information

10.7717/peerj.15373/supp-1 Supplemental Information 1 Uncropped Gels/Blots of Fig. 4A

10.7717/peerj.15373/supp-2 Supplemental Information 2 Raw data of Figs. 4B–4C

10.7717/peerj.15373/supp-3 Supplemental Information 3 Raw data of Figs. 4D–4E

10.7717/peerj.15373/supp-4 Supplemental Information 4 Raw data of Figs. 5A–5B

10.7717/peerj.15373/supp-5 Supplemental Information 5 Raw images of Fig. 5A-B-SMMC-7721-200X

10.7717/peerj.15373/supp-6 Supplemental Information 6 Raw images of Fig. 5A-B-BEL-7404-200X

10.7717/peerj.15373/supp-7 Supplemental Information 7 Raw data of Figs. 5C–5D

10.7717/peerj.15373/supp-8 Supplemental Information 8 Raw images of Fig. 5C-D-SMMC-7721-200X

10.7717/peerj.15373/supp-9 Supplemental Information 9 Raw images of Fig. 5C-D-BEL-7404-200X

## Additional Information and Declarations

### Data Availability

The following information was supplied regarding data availability: The raw data are available in the Supplemental Files.
