# Supplementary figures and images for: Clinical significance of FBXO43 in hepatocellular carcinoma and its impact on tumor cell proliferation, migration and invasion
Source: PeerJ. 2023 May 22;11:e15373. doi: 10.7717/peerj.15373 (PMC10211365; doi:10.7717/peerj.15373)

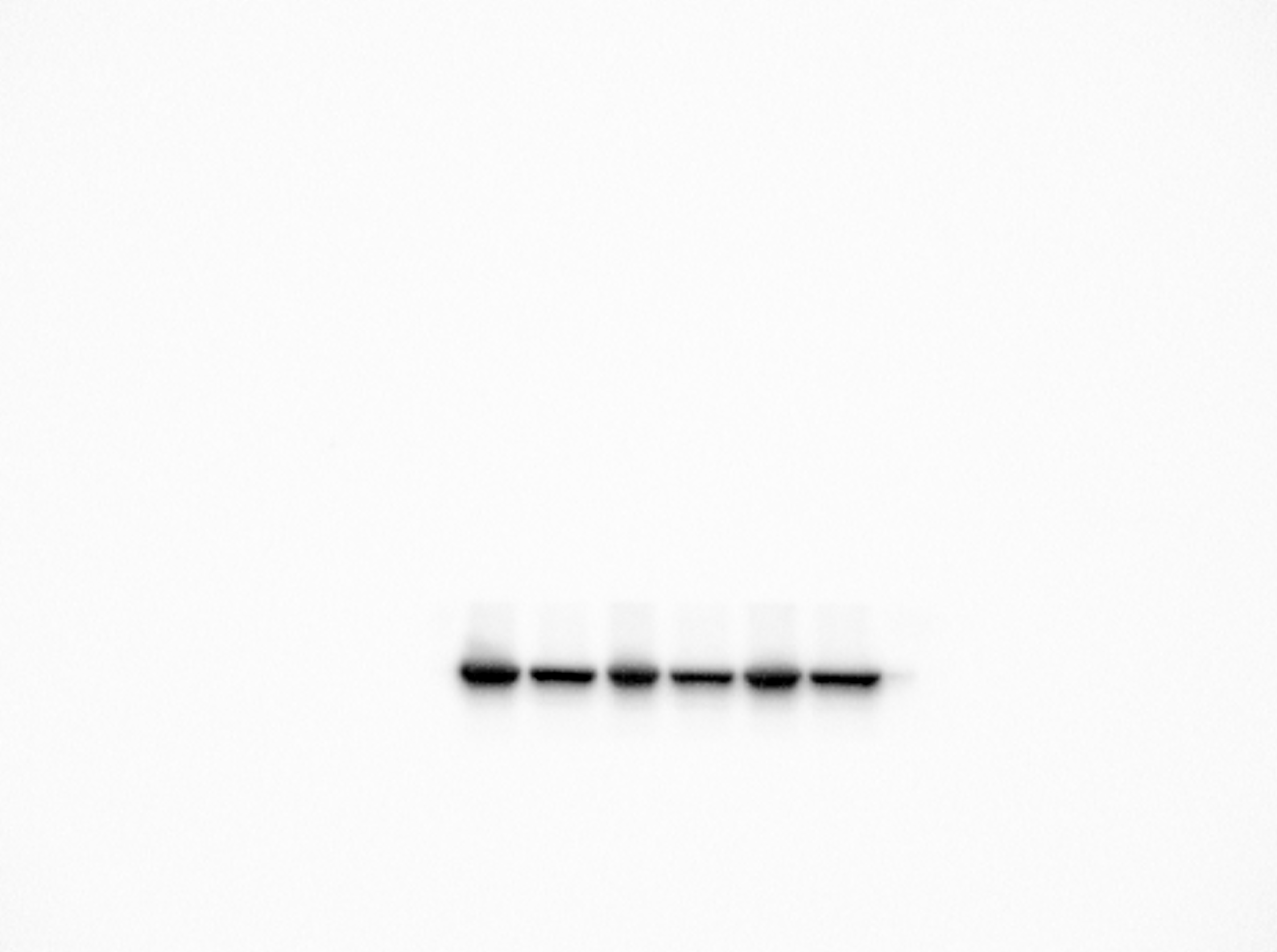

Supplement: Supplemental Information 1 [file peerj-11-15373-s001.zip › Figure 4A-Uncropped Gels-Blots/FBXO43-BEL/FBXO43-BEL.tif]

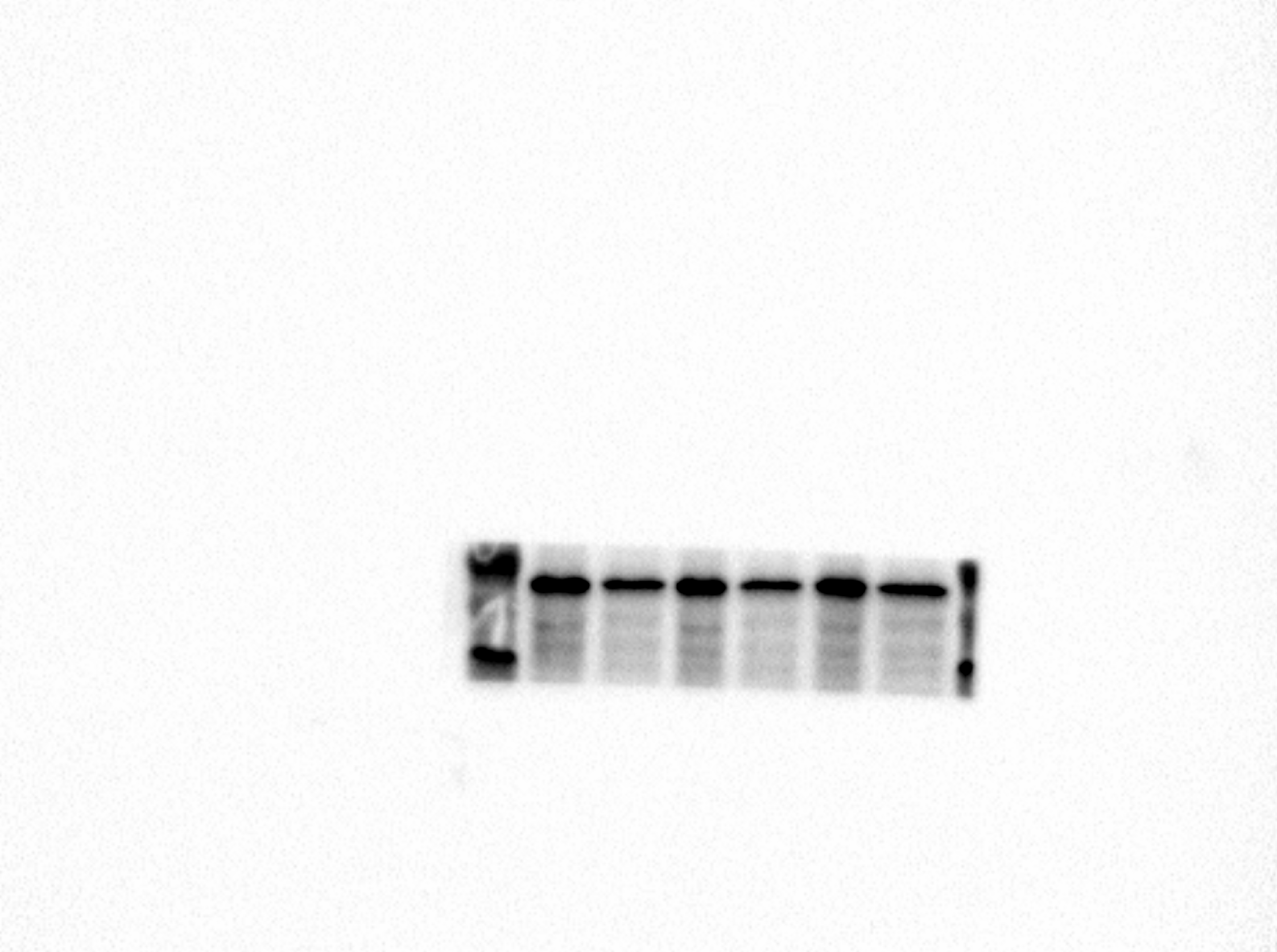

Supplement: Supplemental Information 1 [file peerj-11-15373-s001.zip › Figure 4A-Uncropped Gels-Blots/FBXO43-SMMC/FBXO43-SMMC.tif]

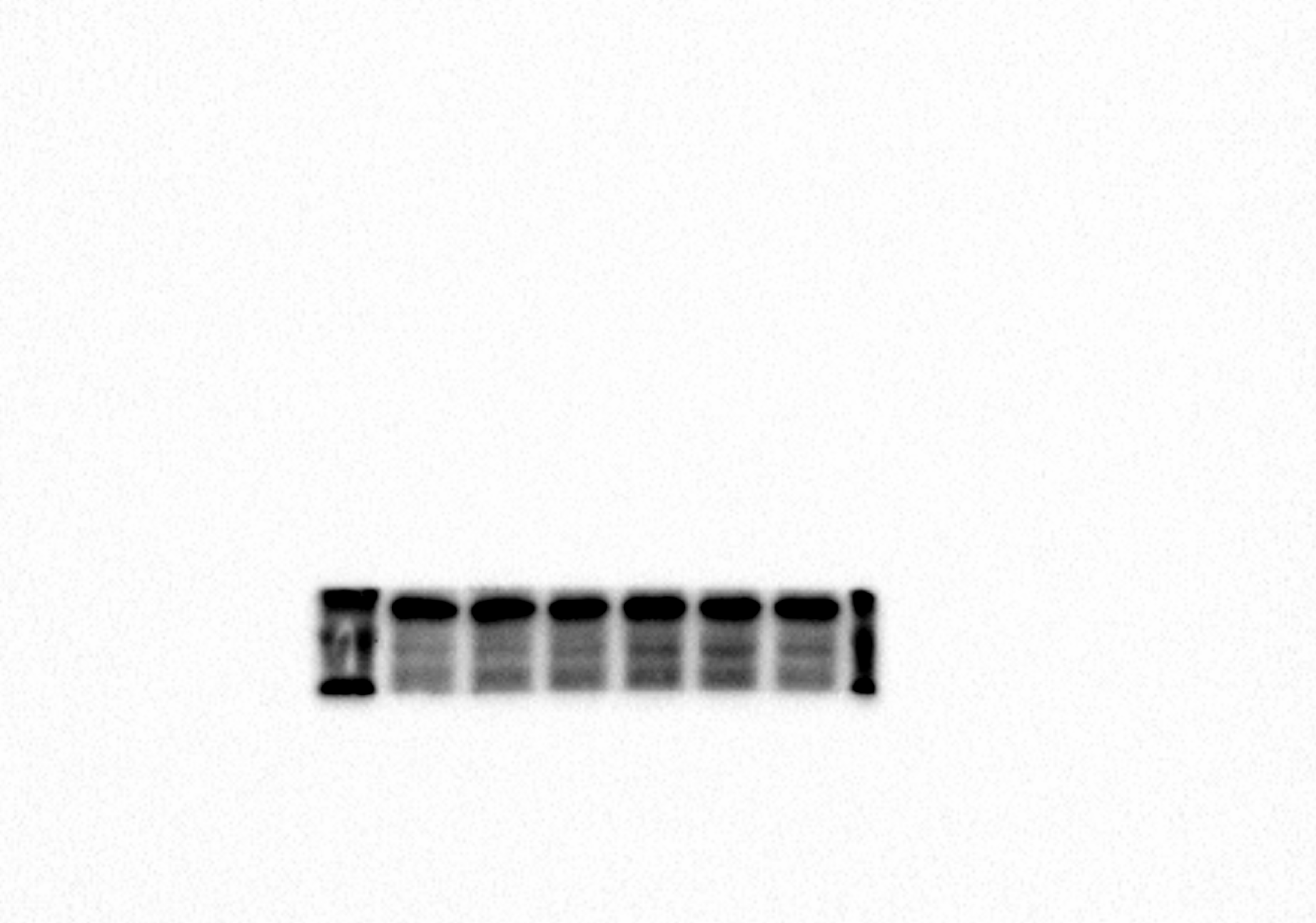

Supplement: Supplemental Information 1 [file peerj-11-15373-s001.zip › Figure 4A-Uncropped Gels-Blots/GAPDH-BEL/GAPDH-BEL.tif]

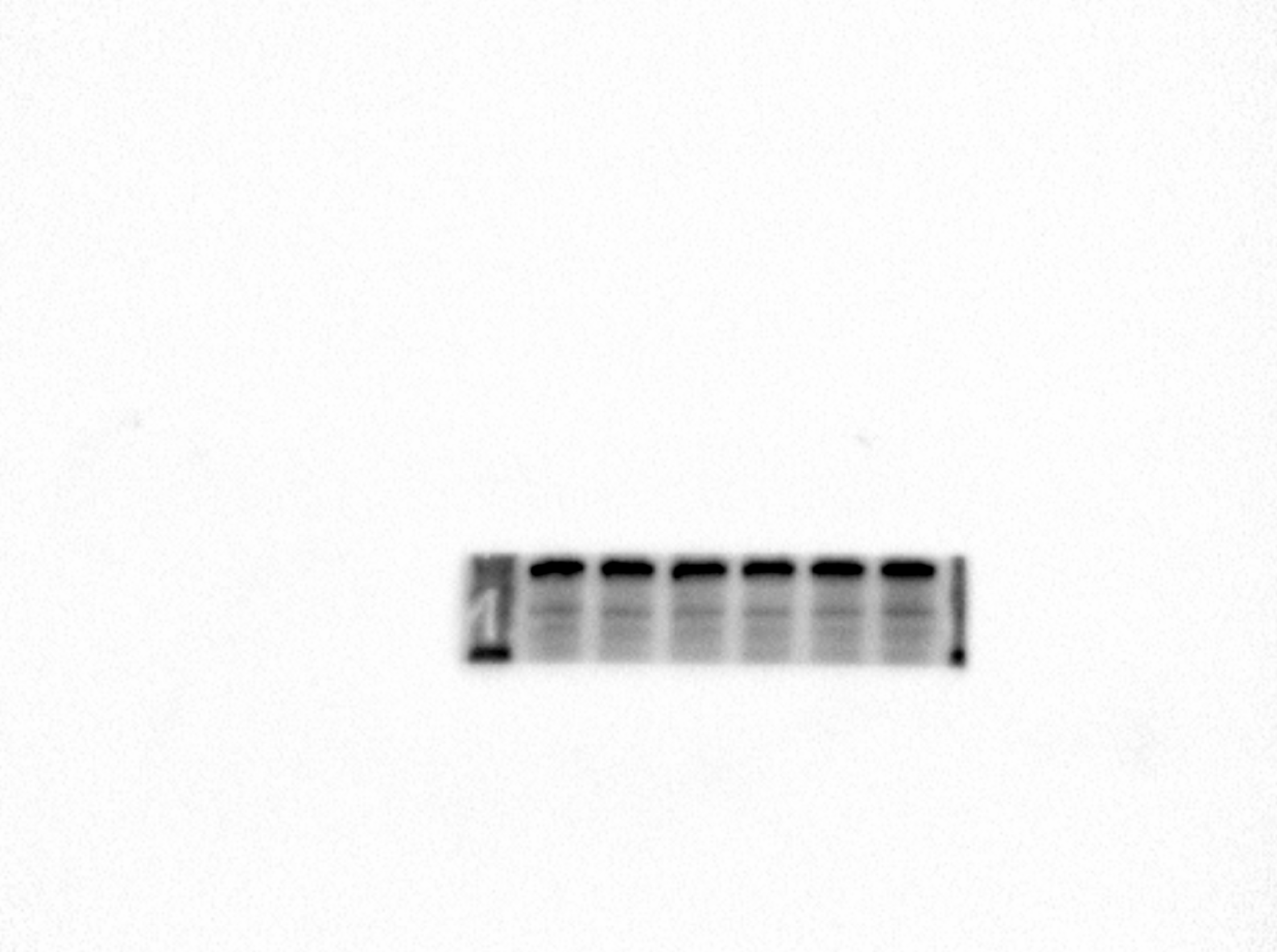

Supplement: Supplemental Information 1 [file peerj-11-15373-s001.zip › Figure 4A-Uncropped Gels-Blots/GAPDH-SMMC/GAPDH-SMMC.tif]

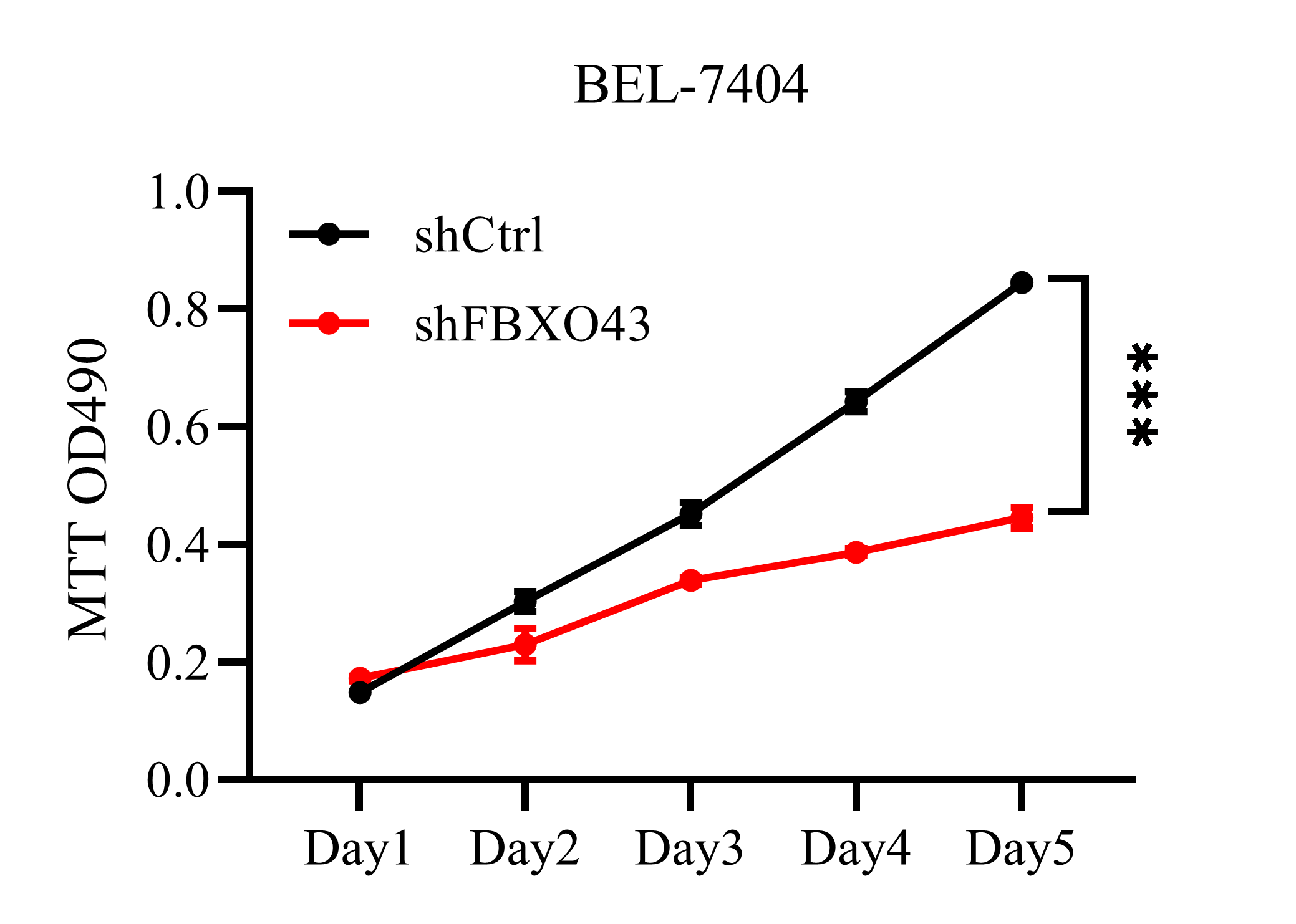

Supplement: Supplemental Information 2 [file peerj-11-15373-s002.zip › Figure 4B-C/BEL-7404.png]

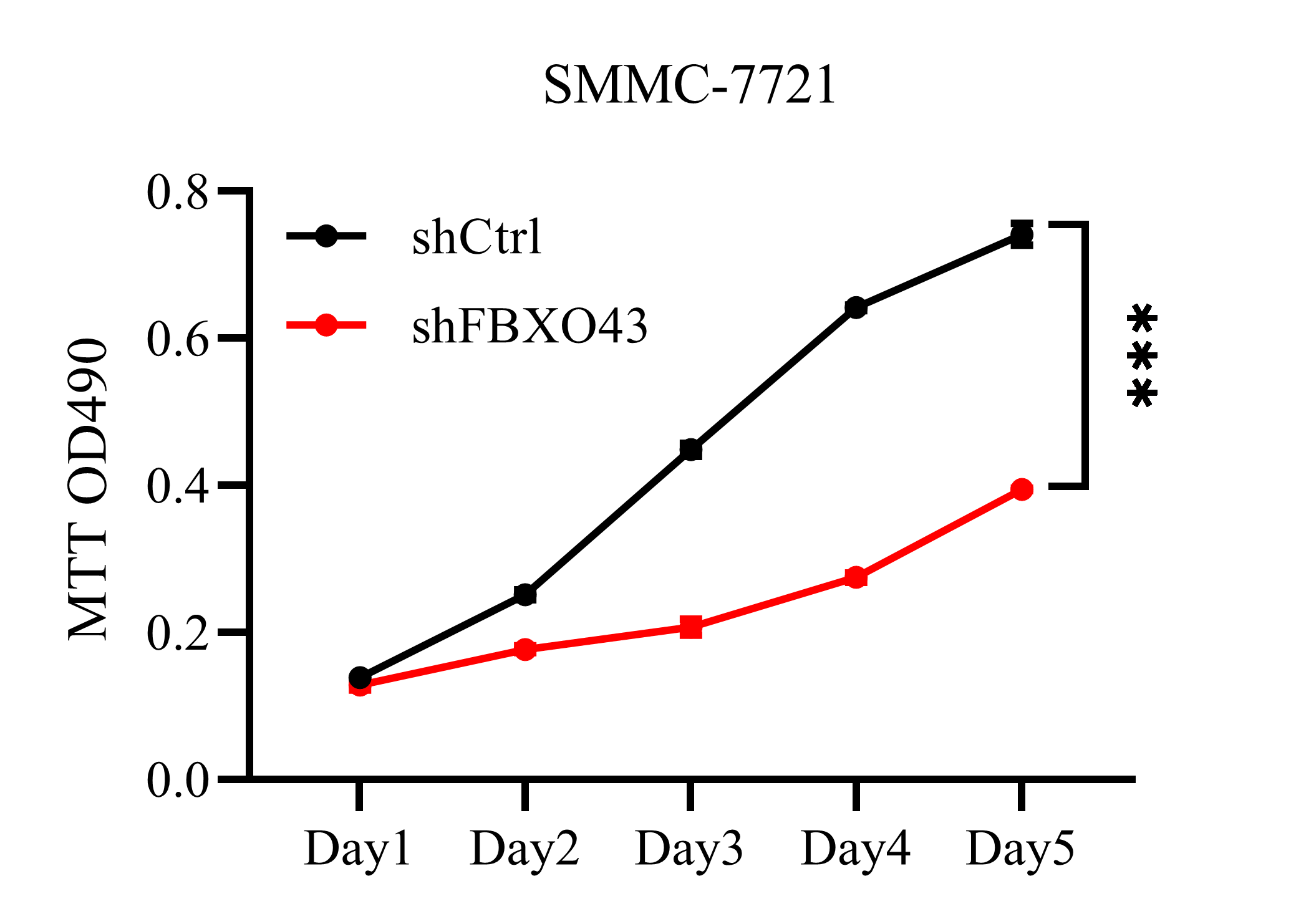

Supplement: Supplemental Information 2 [file peerj-11-15373-s002.zip › Figure 4B-C/SMMC-7721.png]

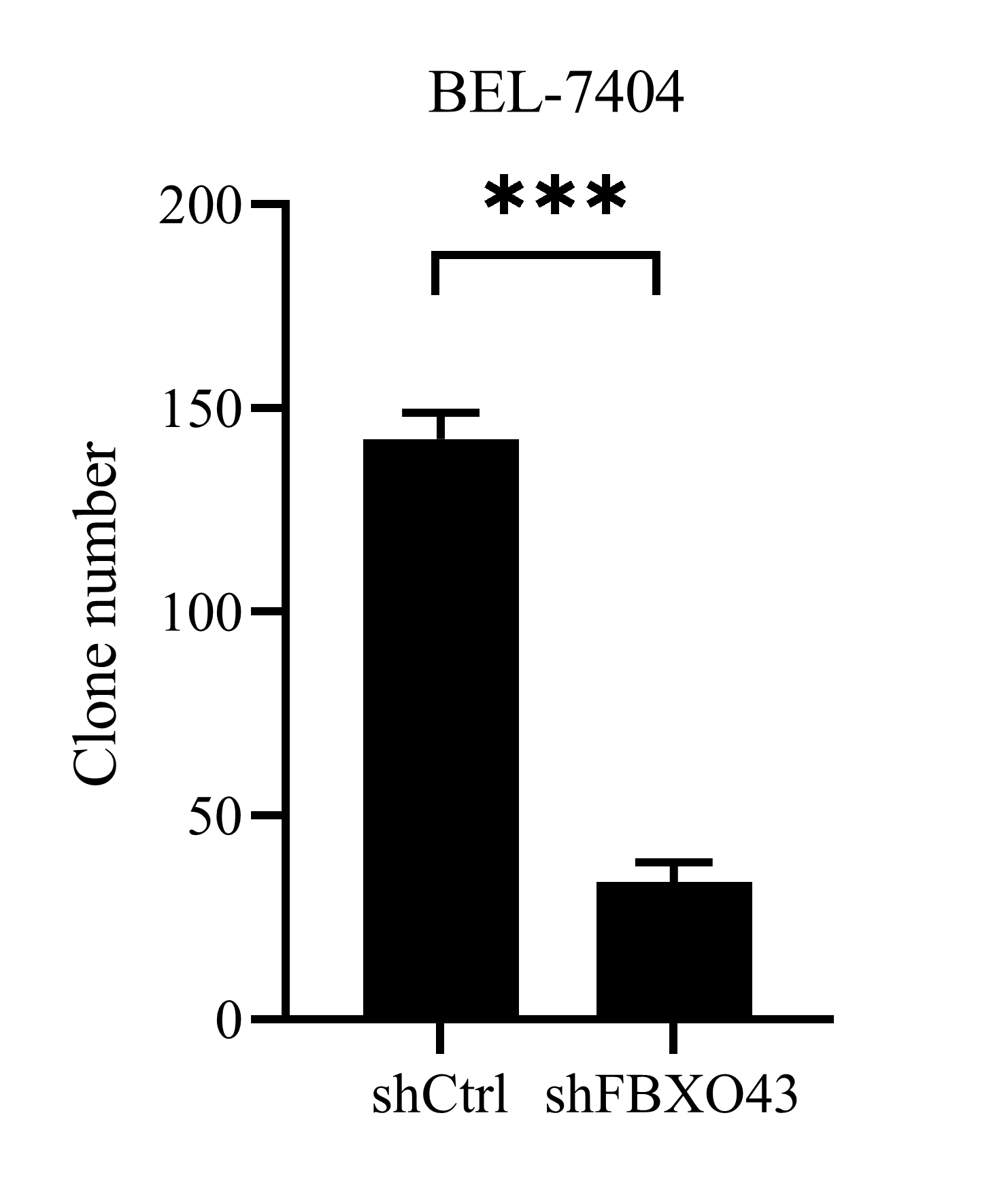

Supplement: Supplemental Information 3 [file peerj-11-15373-s003.zip › Figure 4D-E/BEL-7404.png]

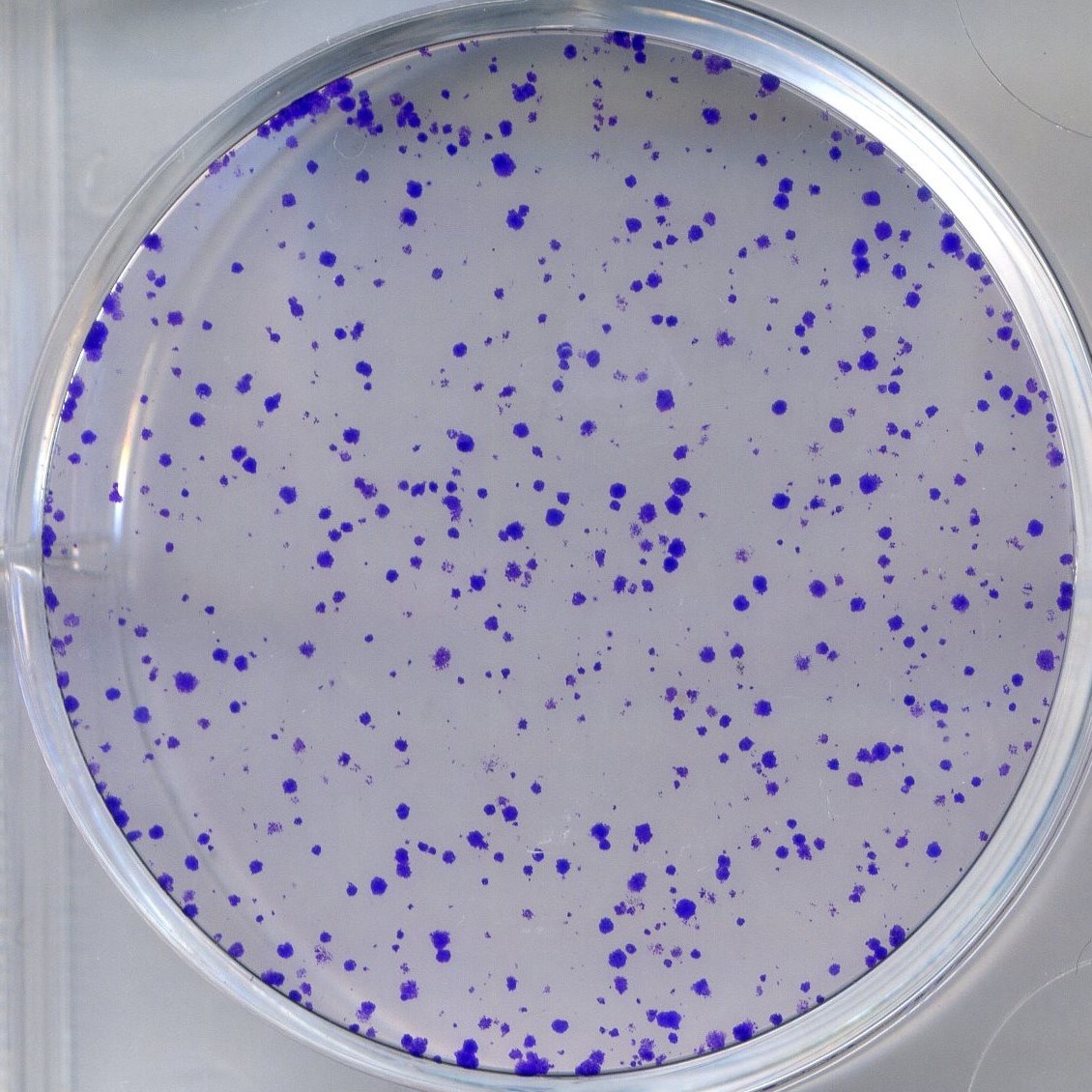

Supplement: Supplemental Information 3 [file peerj-11-15373-s003.zip › Figure 4D-E/BEL-shCtrl.jpg]

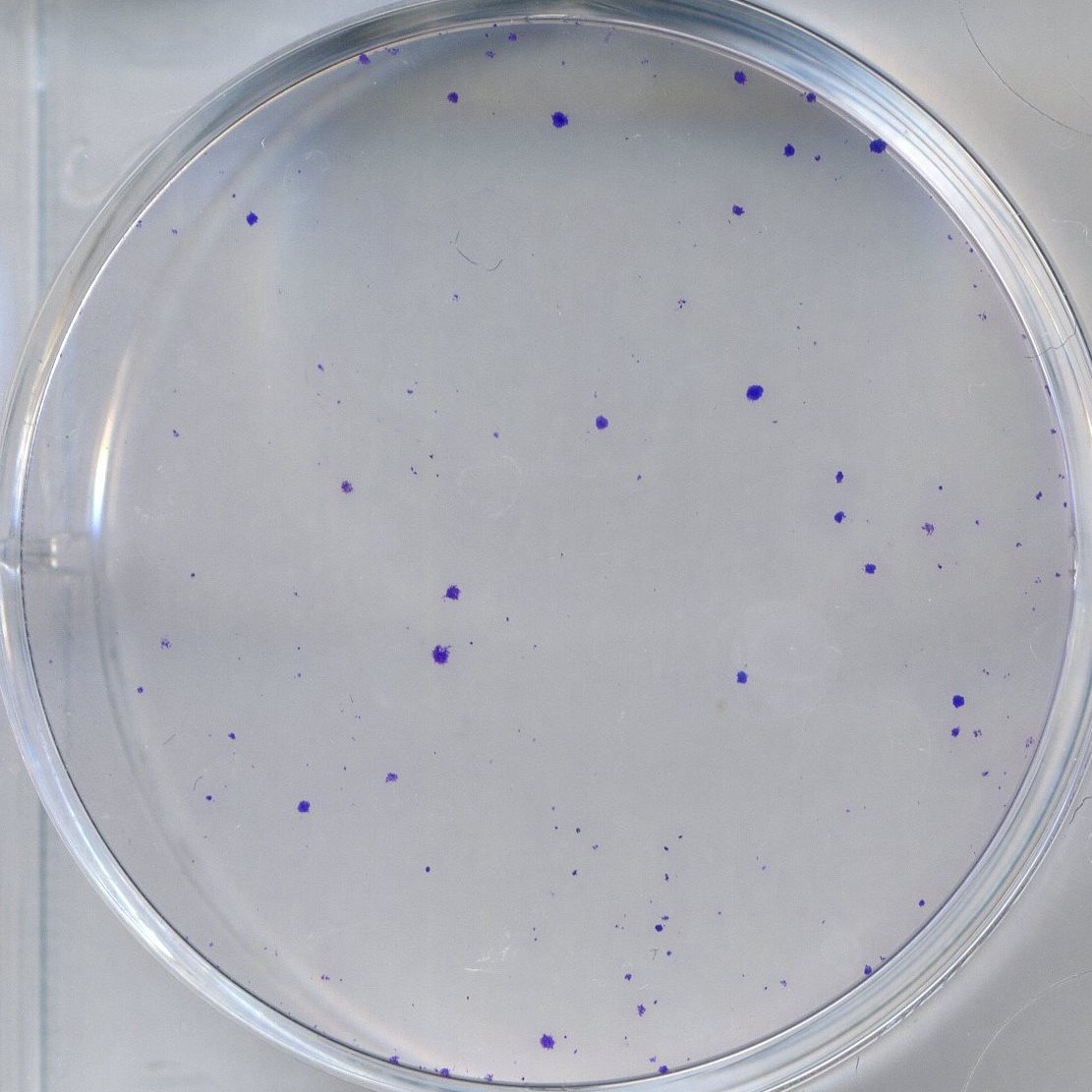

Supplement: Supplemental Information 3 [file peerj-11-15373-s003.zip › Figure 4D-E/BEL-shFBXO43.jpg]

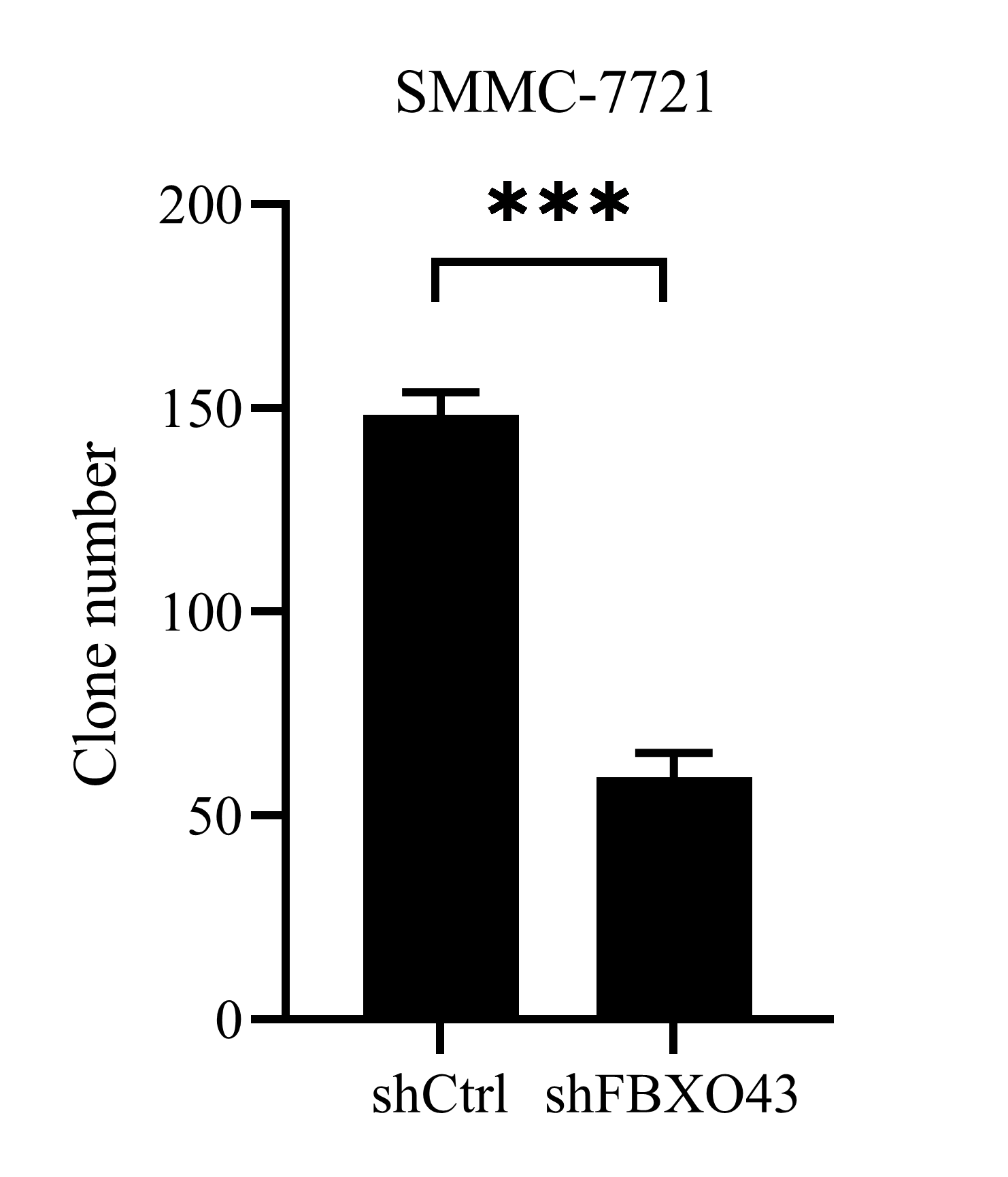

Supplement: Supplemental Information 3 [file peerj-11-15373-s003.zip › Figure 4D-E/SMMC-7721.png]

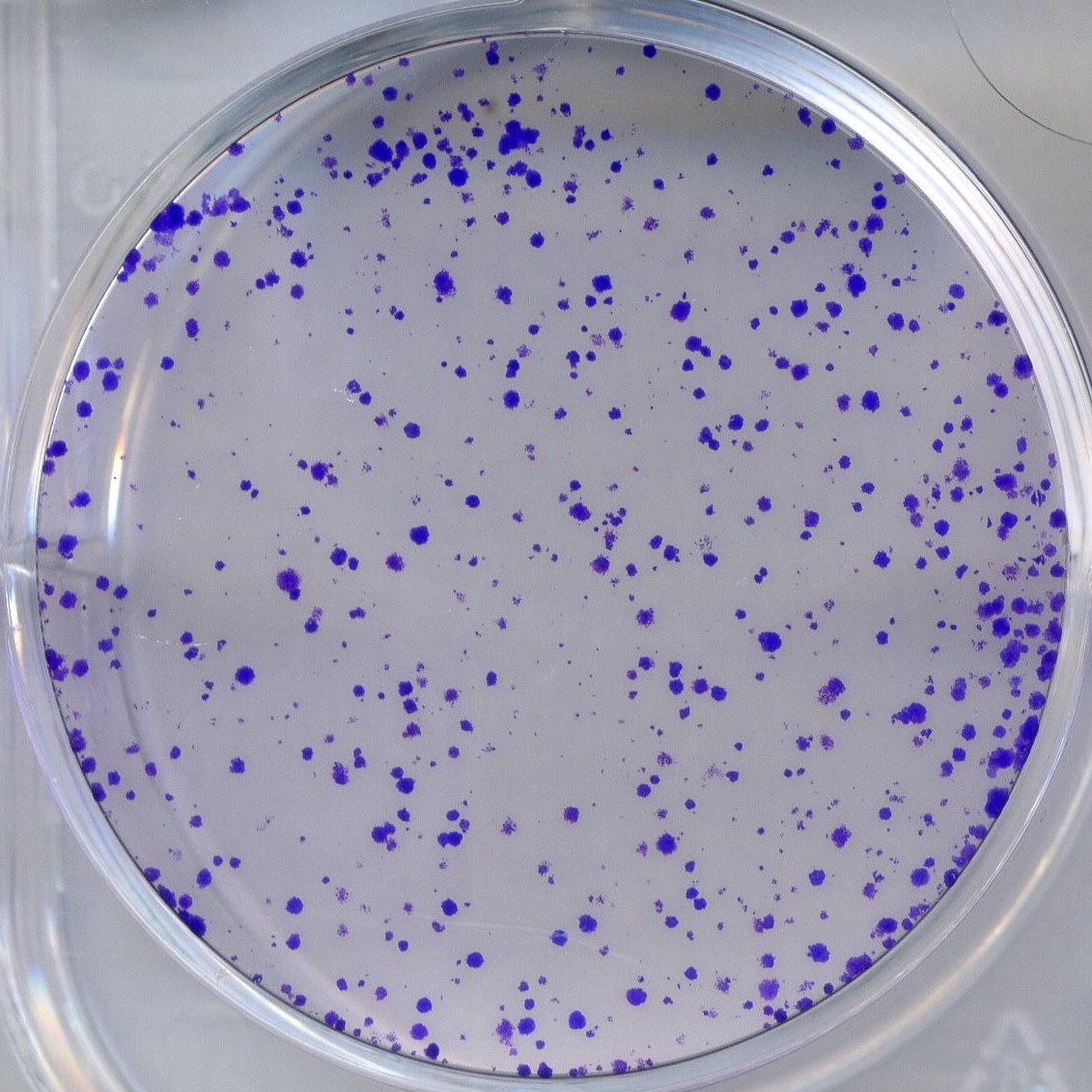

Supplement: Supplemental Information 3 [file peerj-11-15373-s003.zip › Figure 4D-E/SMMC-shCtrl.jpg]

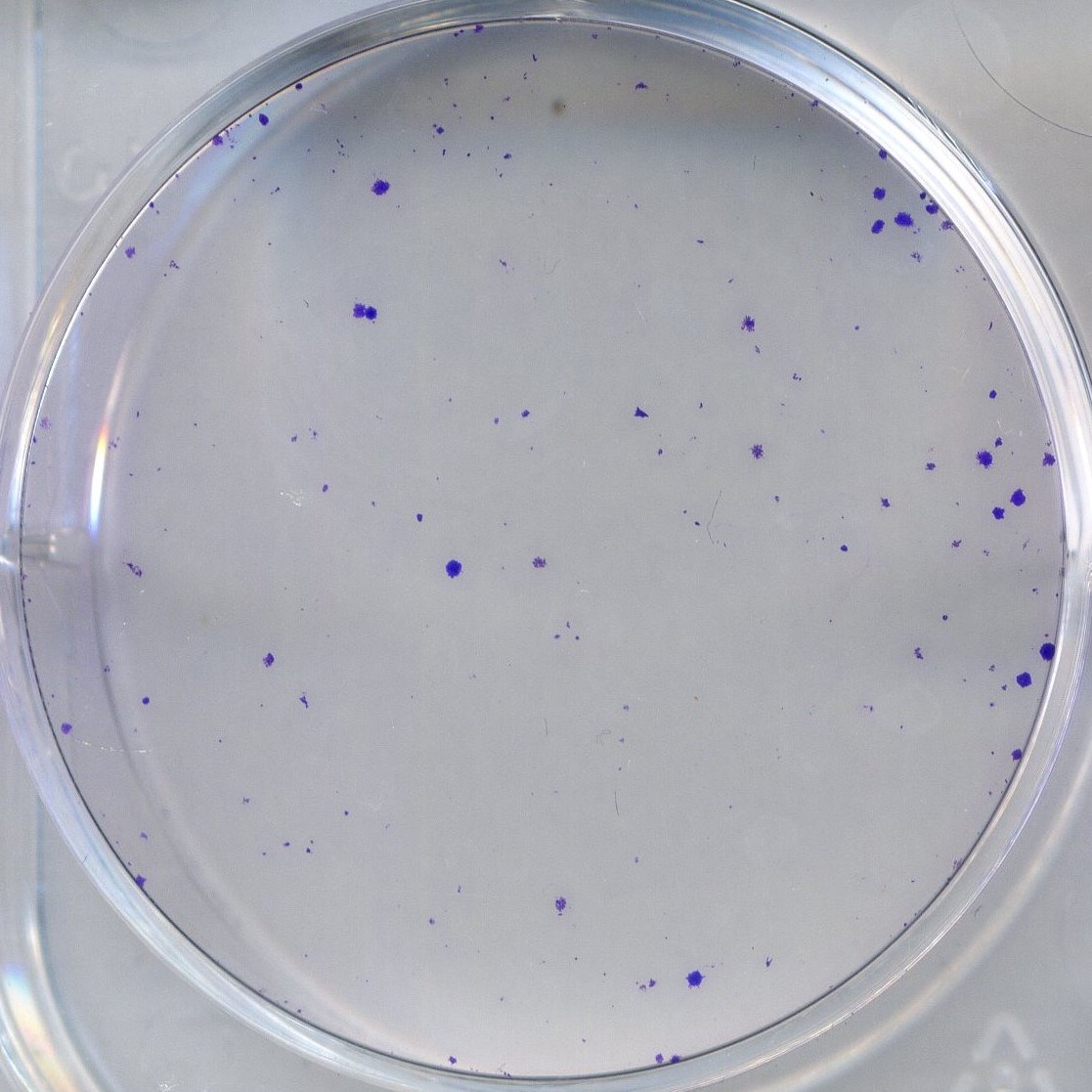

Supplement: Supplemental Information 3 [file peerj-11-15373-s003.zip › Figure 4D-E/SMMC-shFBXO43.jpg]

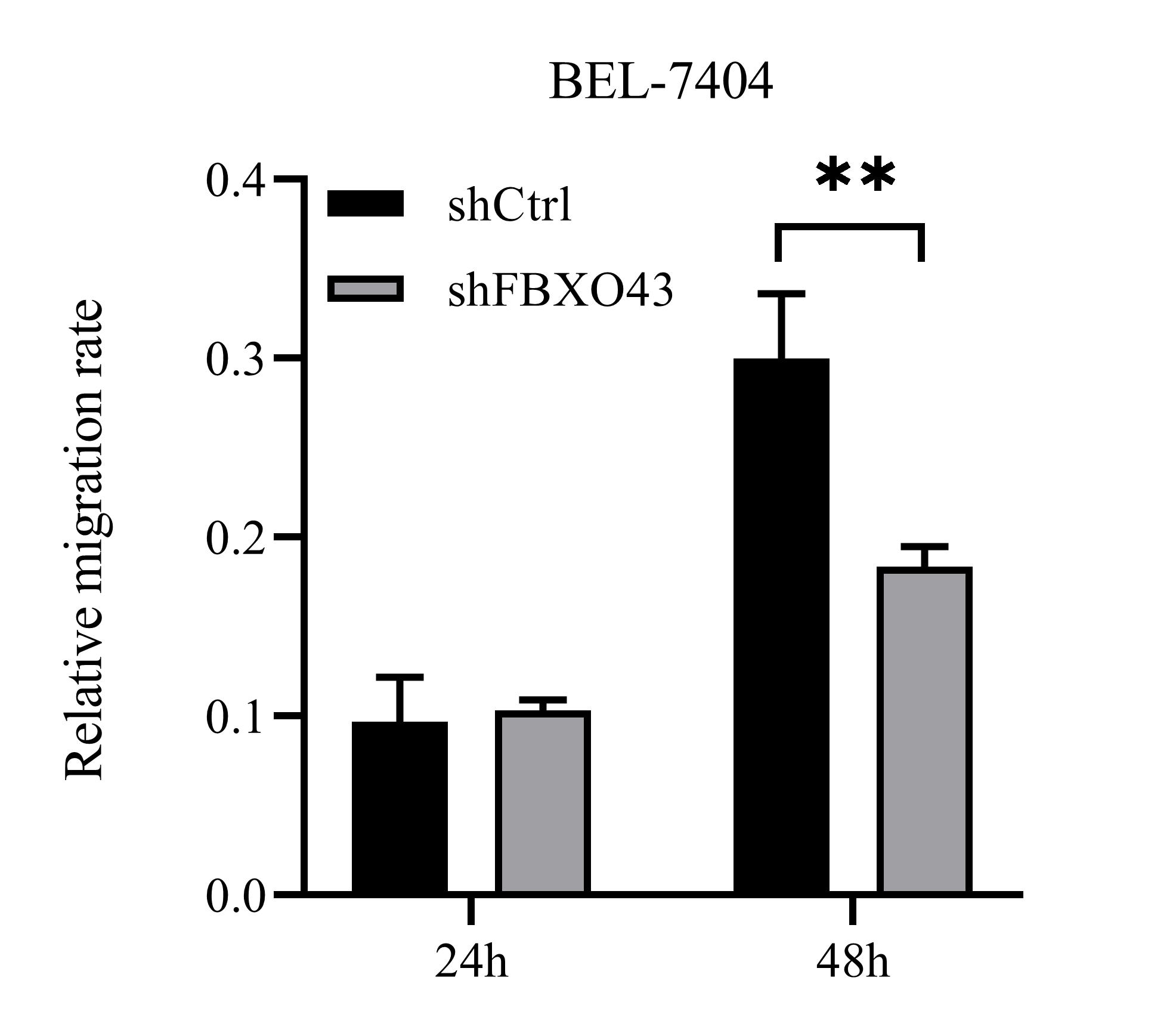

Supplement: Supplemental Information 4 [file peerj-11-15373-s004.zip › Figure 5A-B/BEL-7404.png]

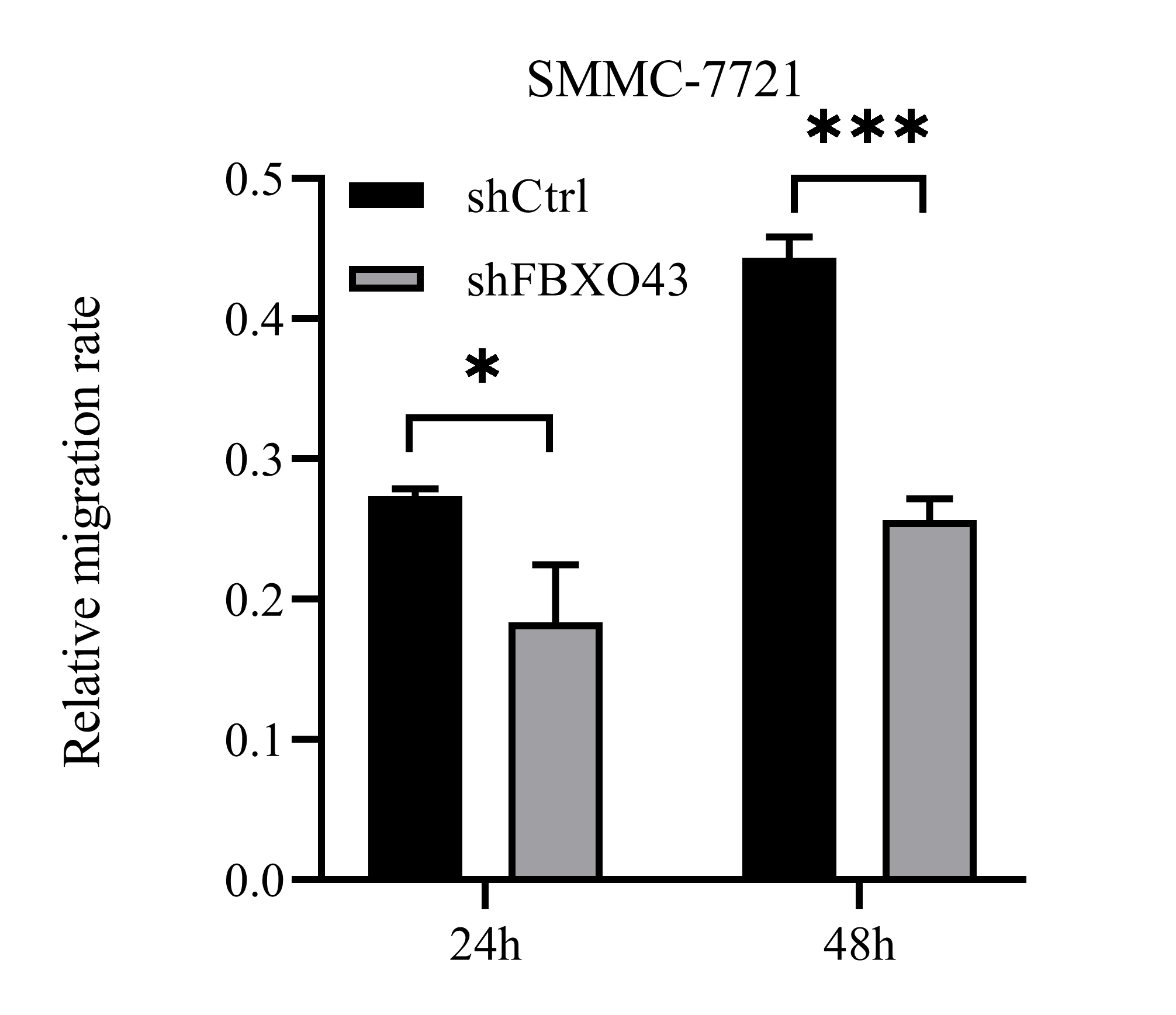

Supplement: Supplemental Information 4 [file peerj-11-15373-s004.zip › Figure 5A-B/SMMC-7721.png]

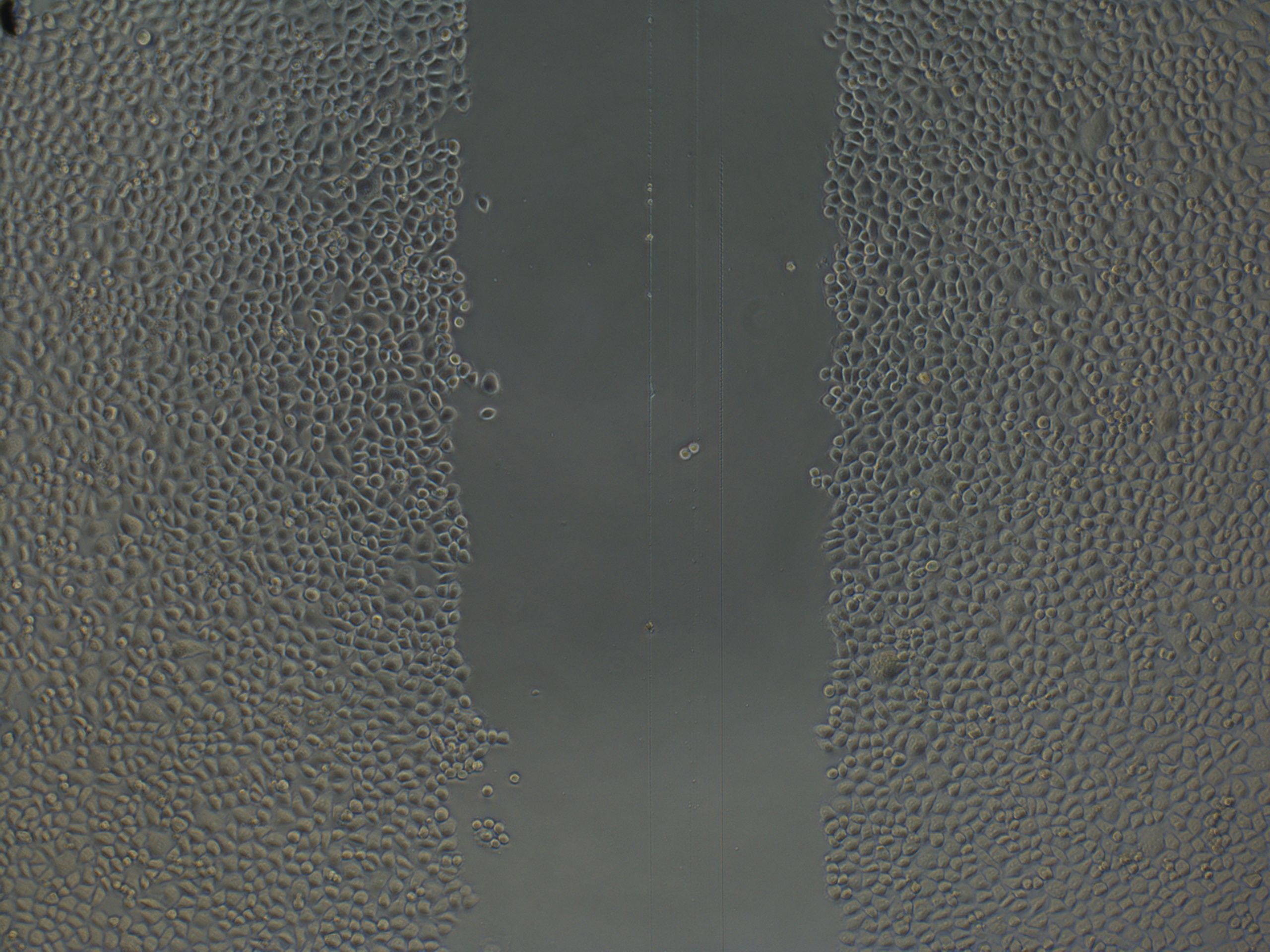

Supplement: Supplemental Information 5 [file peerj-11-15373-s005.zip › Raw data-Figure 5A-B-images-SMMC-7721/shCtrl/0h/1.jpg]

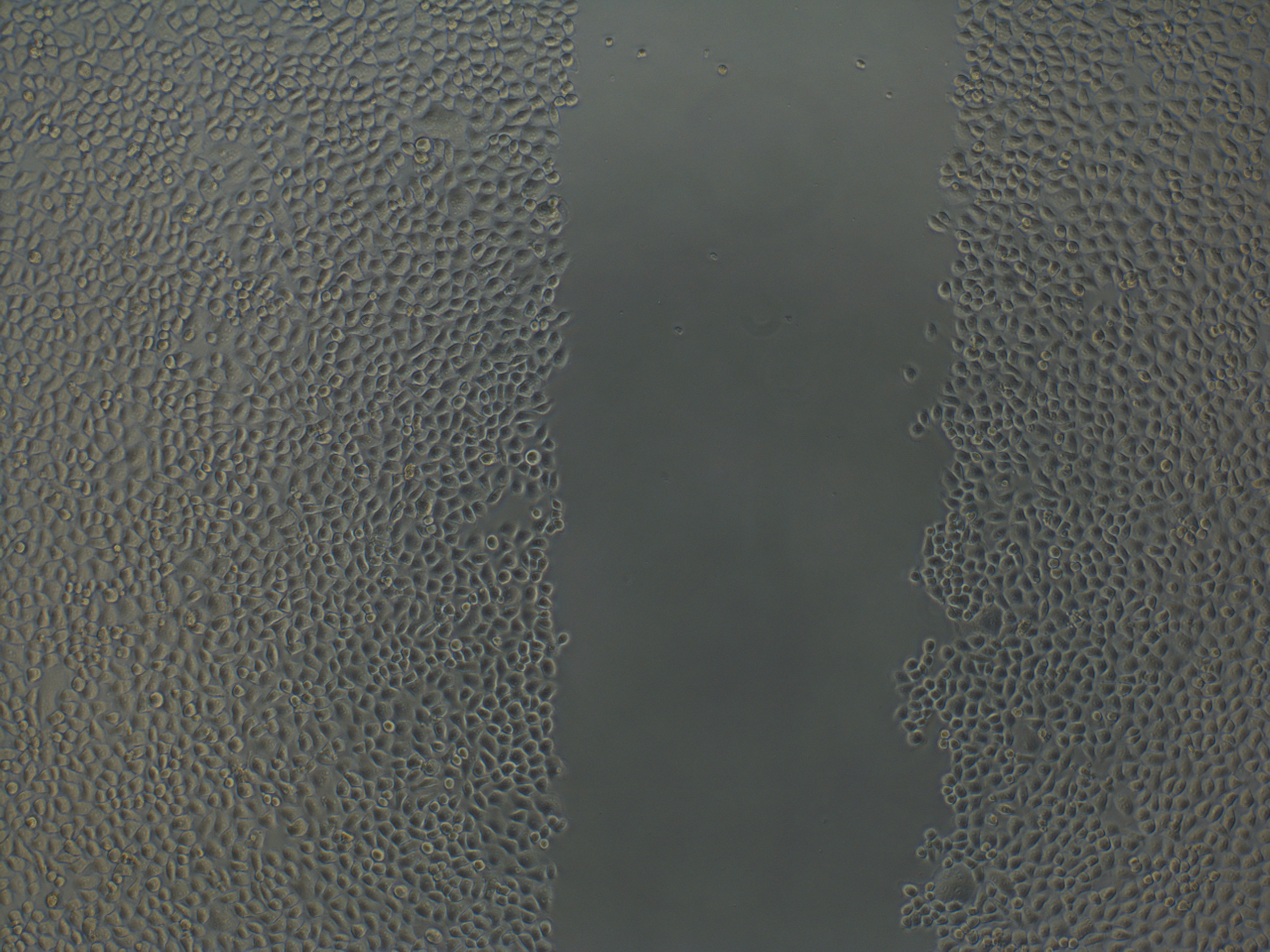

Supplement: Supplemental Information 5 [file peerj-11-15373-s005.zip › Raw data-Figure 5A-B-images-SMMC-7721/shCtrl/0h/2.jpg]

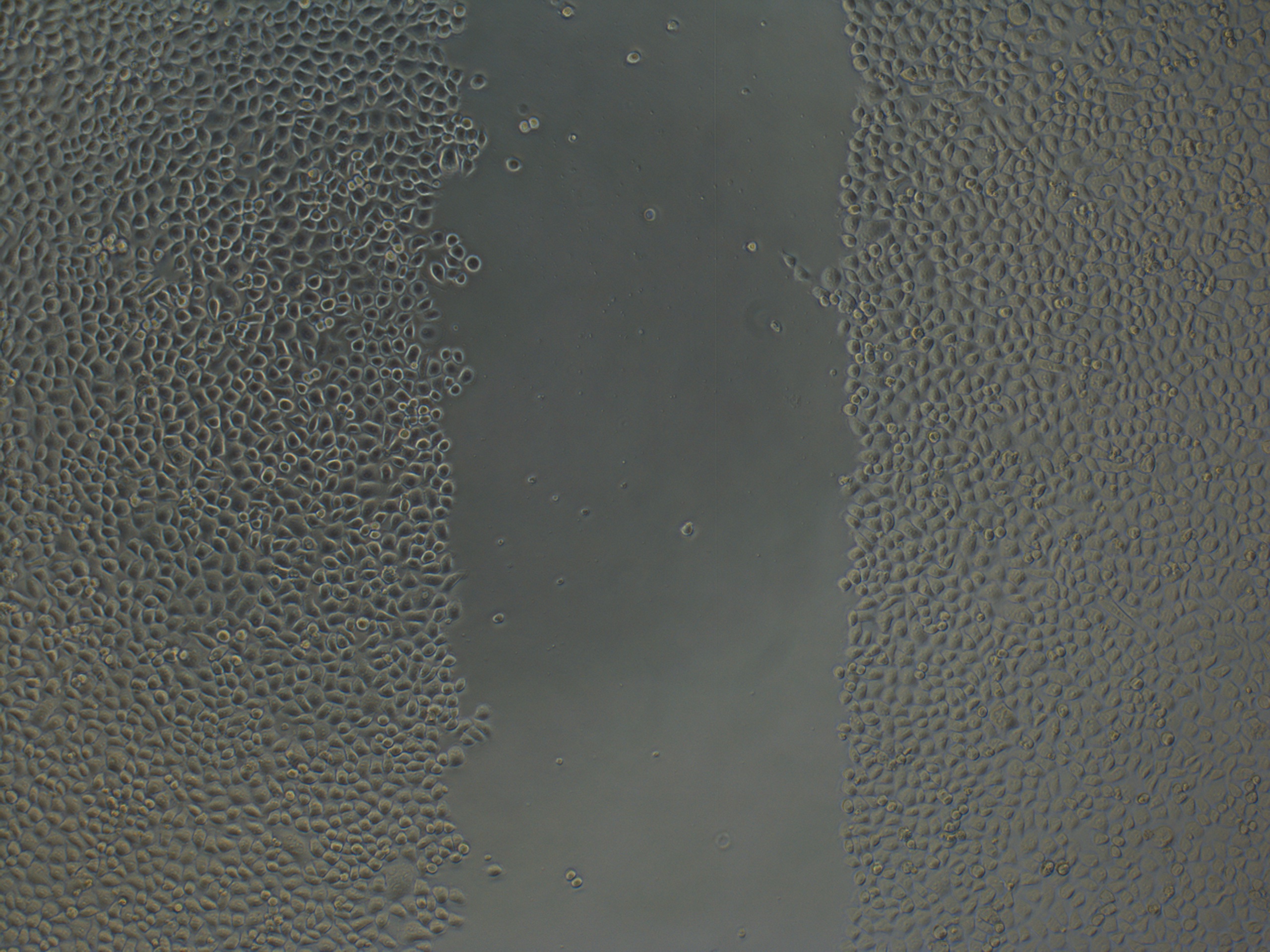

Supplement: Supplemental Information 5 [file peerj-11-15373-s005.zip › Raw data-Figure 5A-B-images-SMMC-7721/shCtrl/0h/3.jpg]

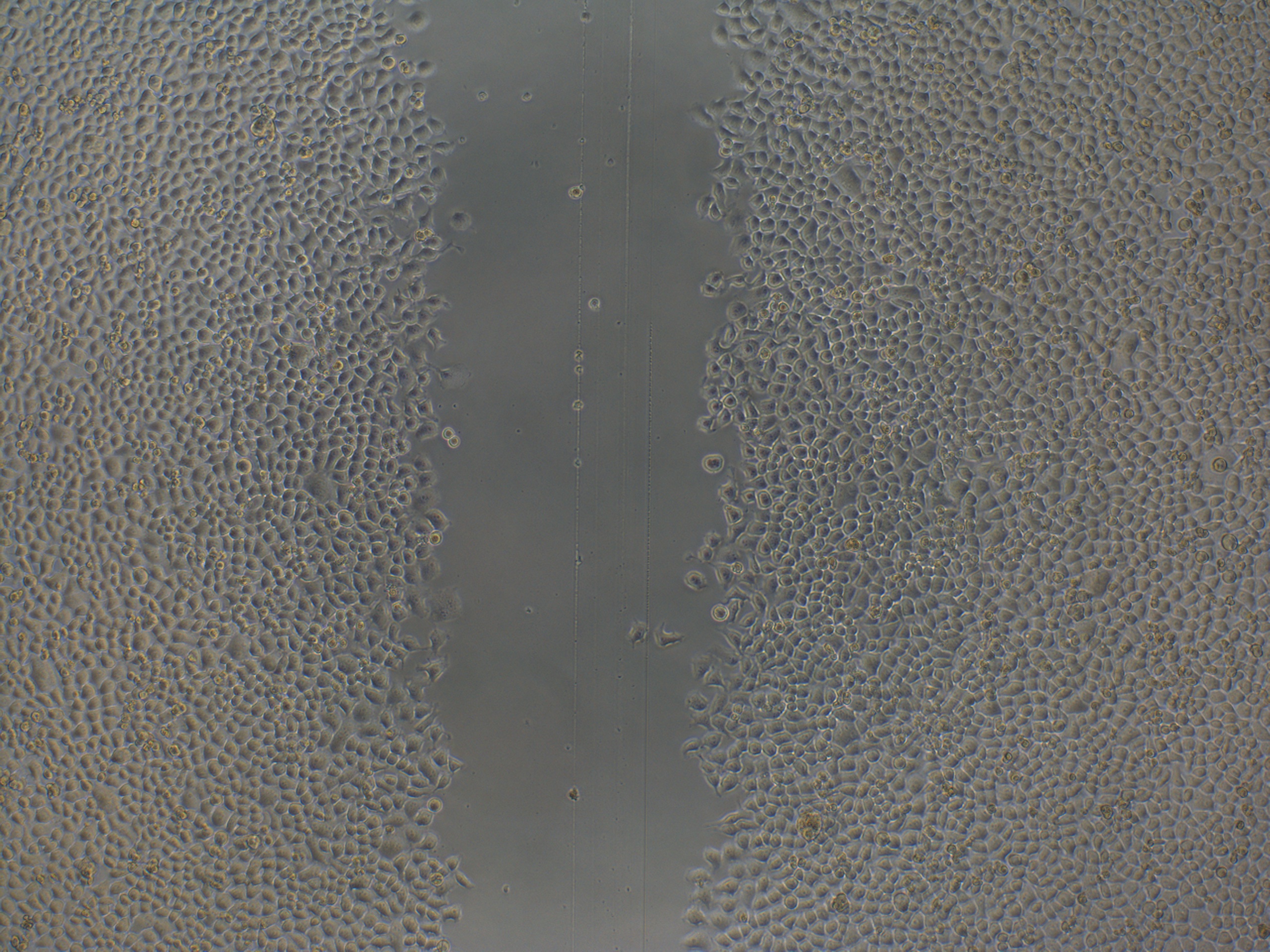

Supplement: Supplemental Information 5 [file peerj-11-15373-s005.zip › Raw data-Figure 5A-B-images-SMMC-7721/shCtrl/24h/1.jpg]

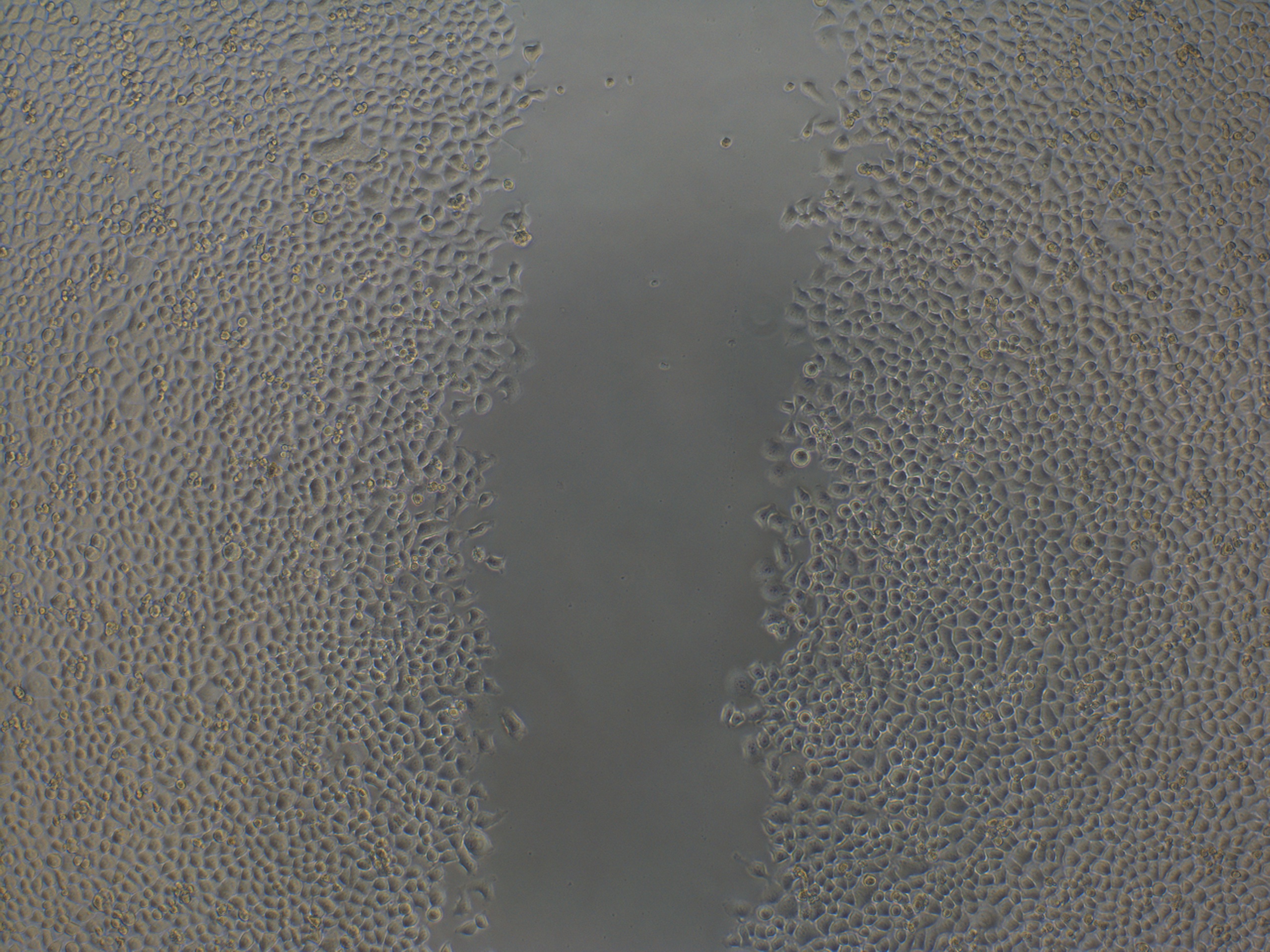

Supplement: Supplemental Information 5 [file peerj-11-15373-s005.zip › Raw data-Figure 5A-B-images-SMMC-7721/shCtrl/24h/2.jpg]

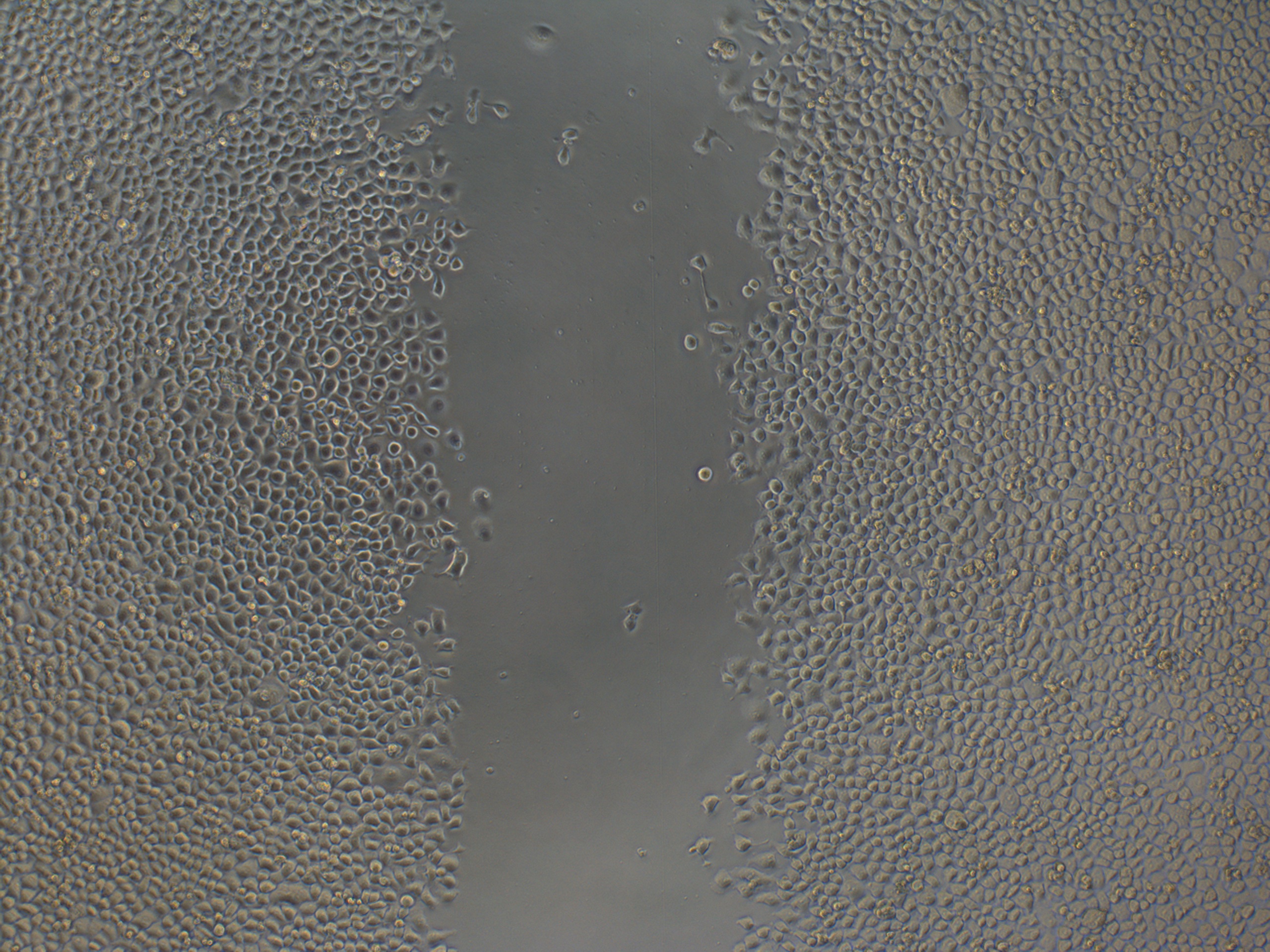

Supplement: Supplemental Information 5 [file peerj-11-15373-s005.zip › Raw data-Figure 5A-B-images-SMMC-7721/shCtrl/24h/3.jpg]

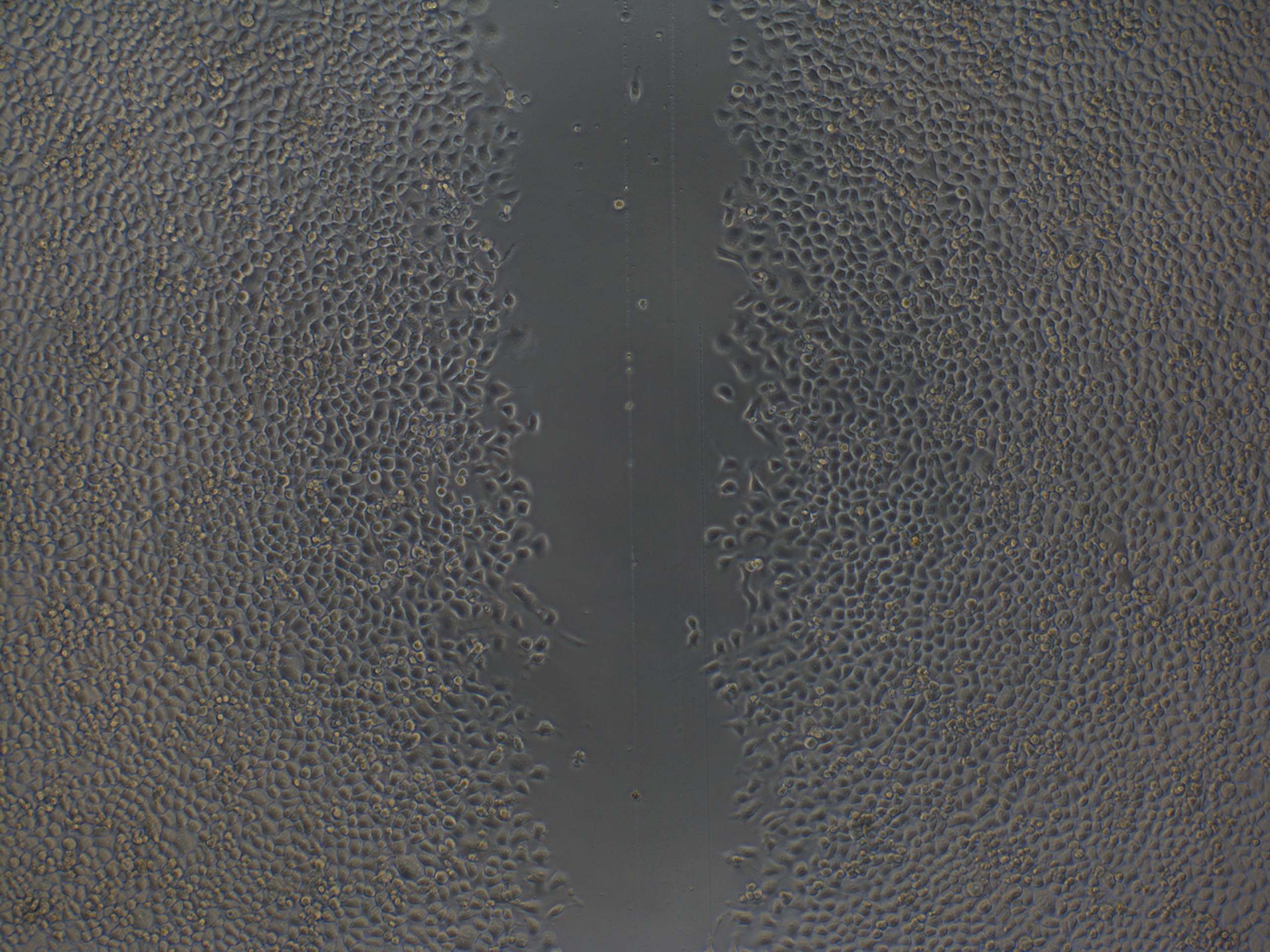

Supplement: Supplemental Information 5 [file peerj-11-15373-s005.zip › Raw data-Figure 5A-B-images-SMMC-7721/shCtrl/48h/1.jpg]

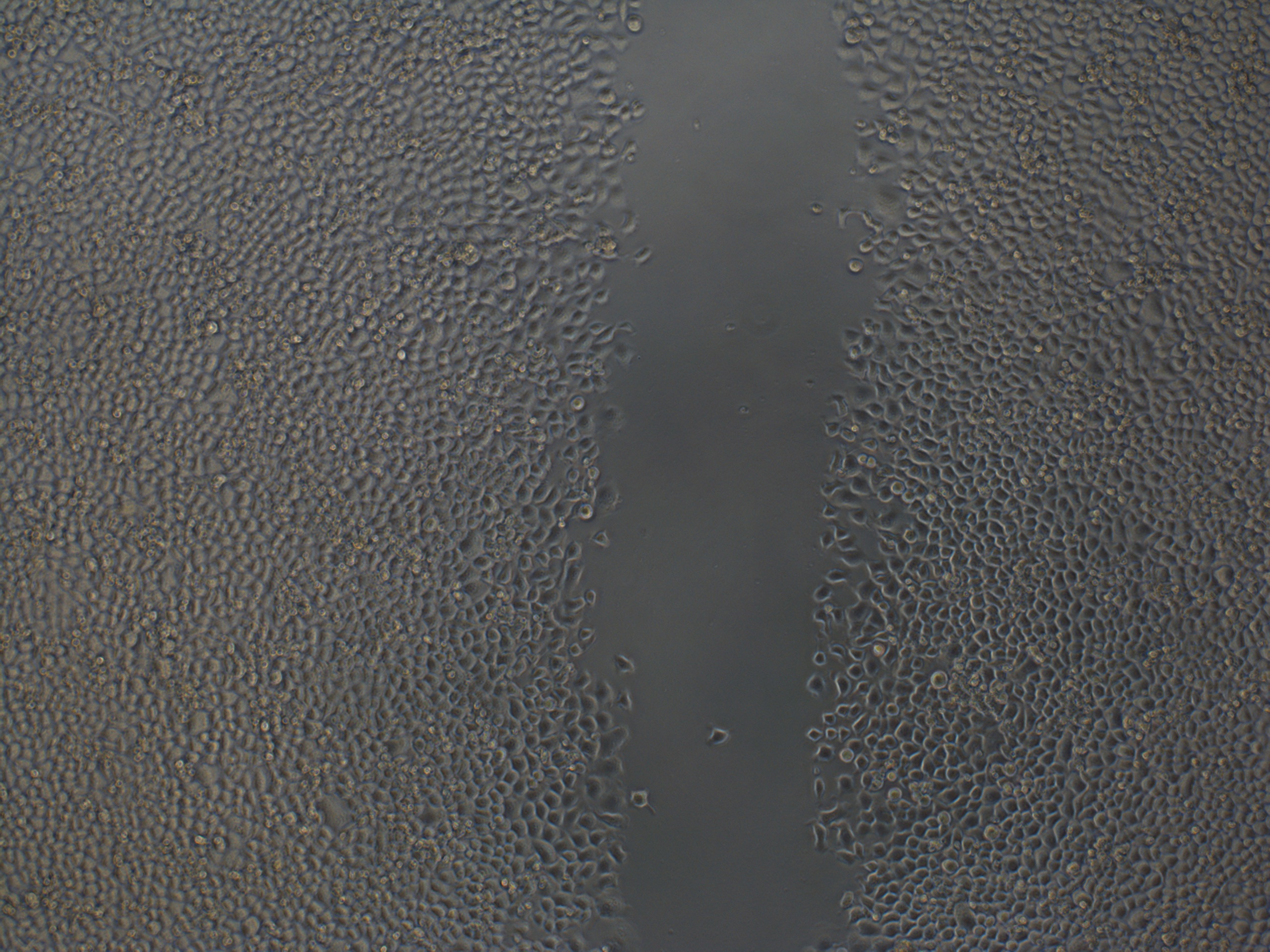

Supplement: Supplemental Information 5 [file peerj-11-15373-s005.zip › Raw data-Figure 5A-B-images-SMMC-7721/shCtrl/48h/2.jpg]

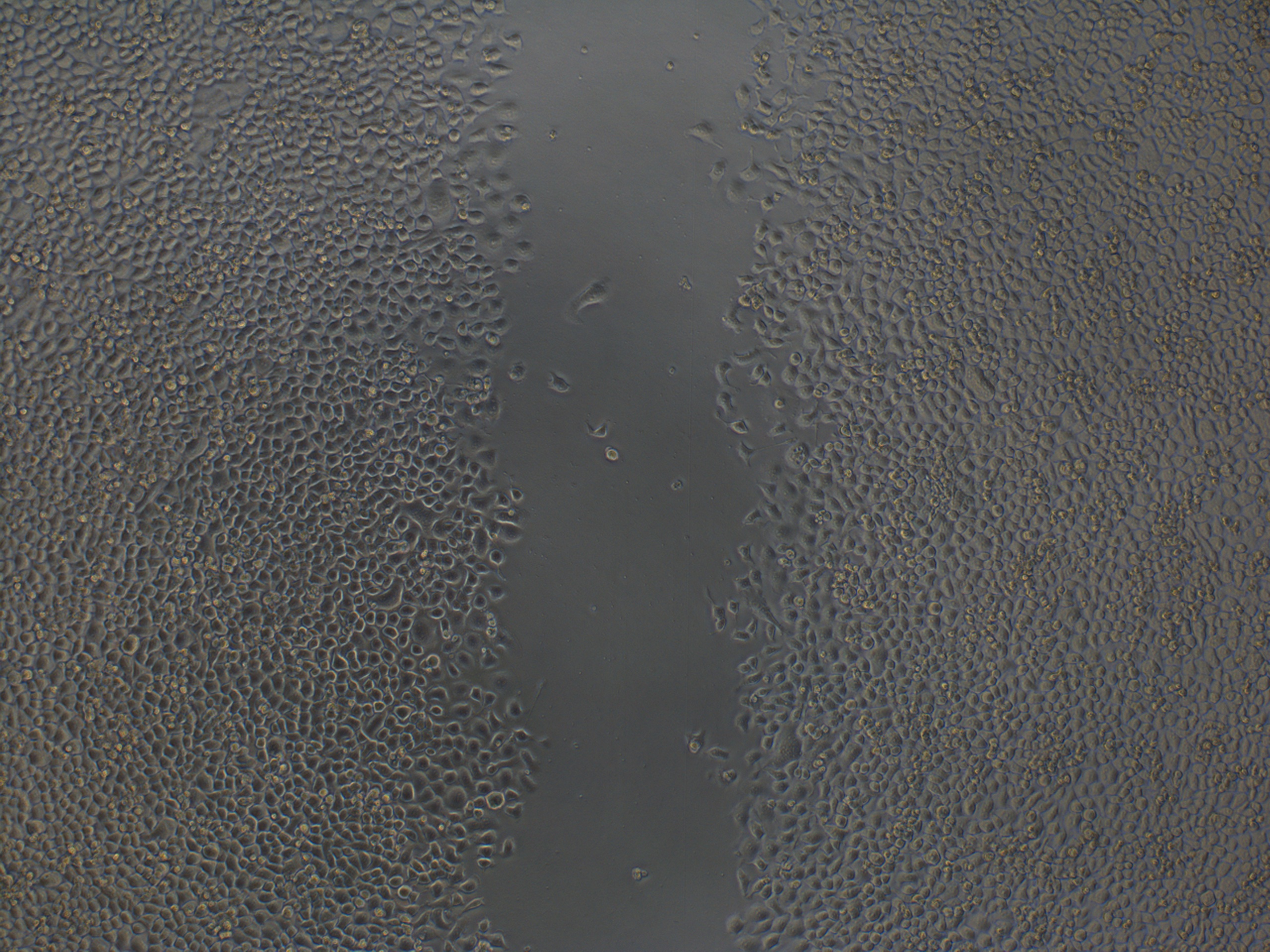

Supplement: Supplemental Information 5 [file peerj-11-15373-s005.zip › Raw data-Figure 5A-B-images-SMMC-7721/shCtrl/48h/3.jpg]

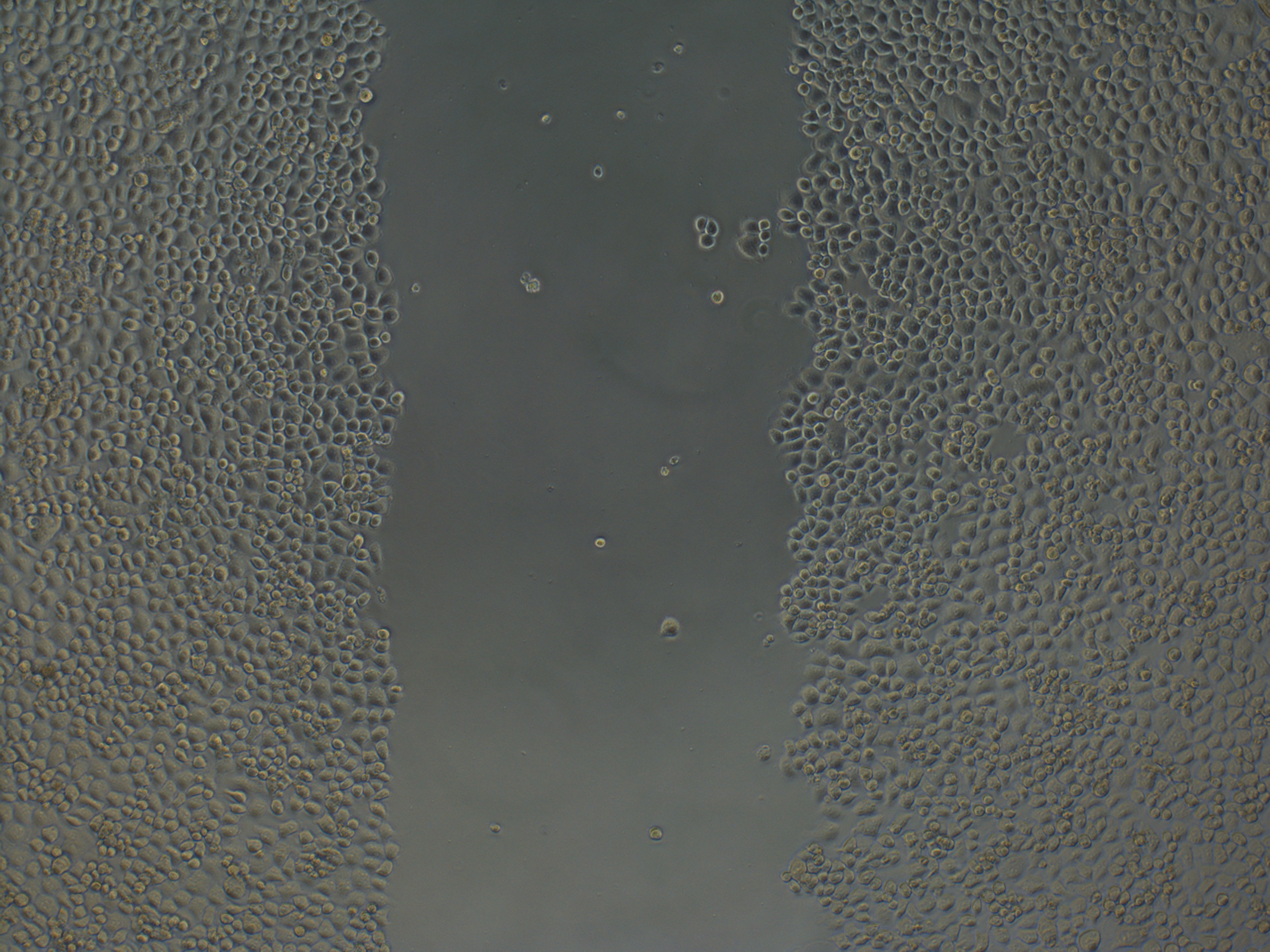

Supplement: Supplemental Information 5 [file peerj-11-15373-s005.zip › Raw data-Figure 5A-B-images-SMMC-7721/shFBXO43/0h/1.jpg]

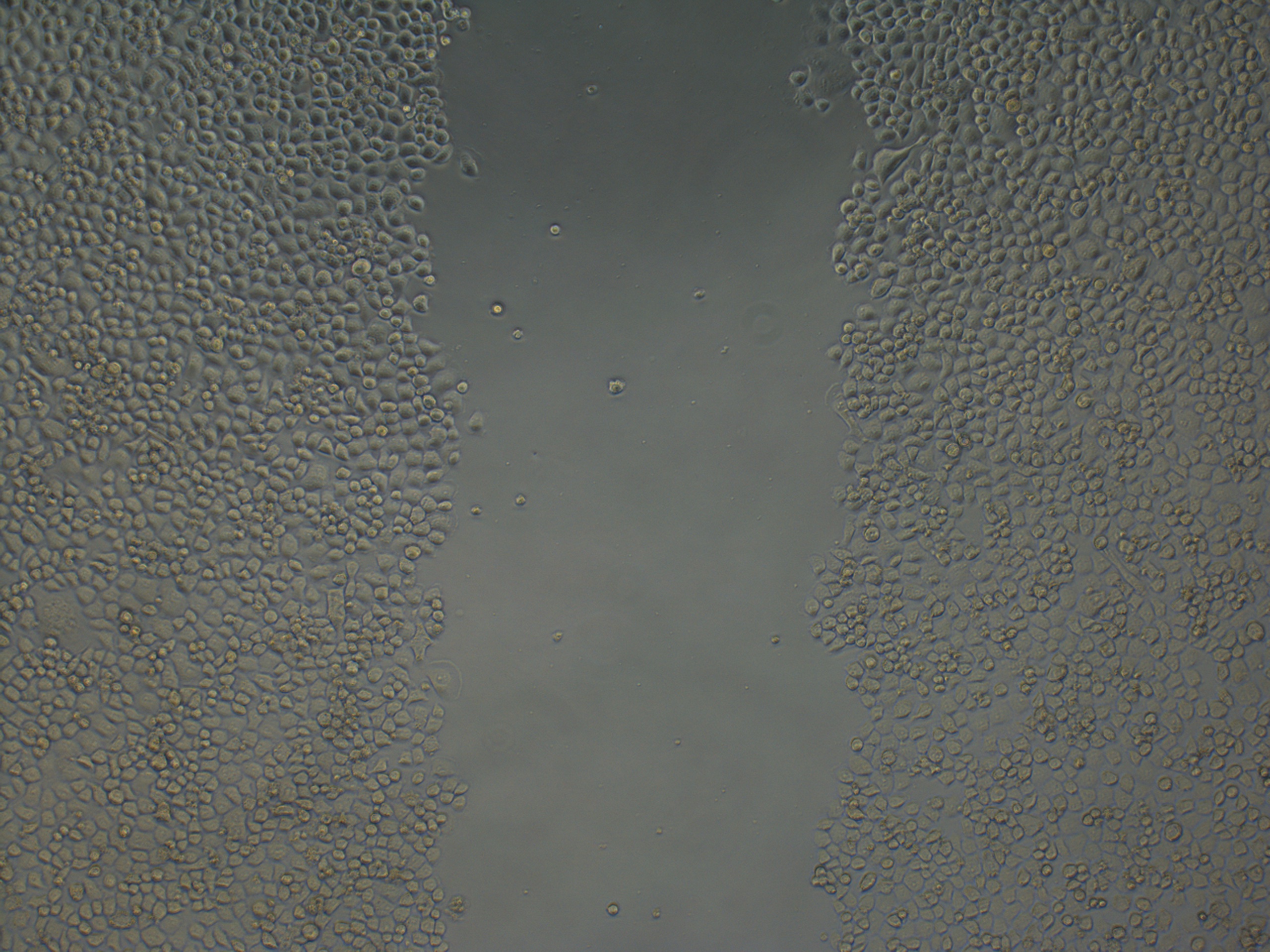

Supplement: Supplemental Information 5 [file peerj-11-15373-s005.zip › Raw data-Figure 5A-B-images-SMMC-7721/shFBXO43/0h/2.jpg]

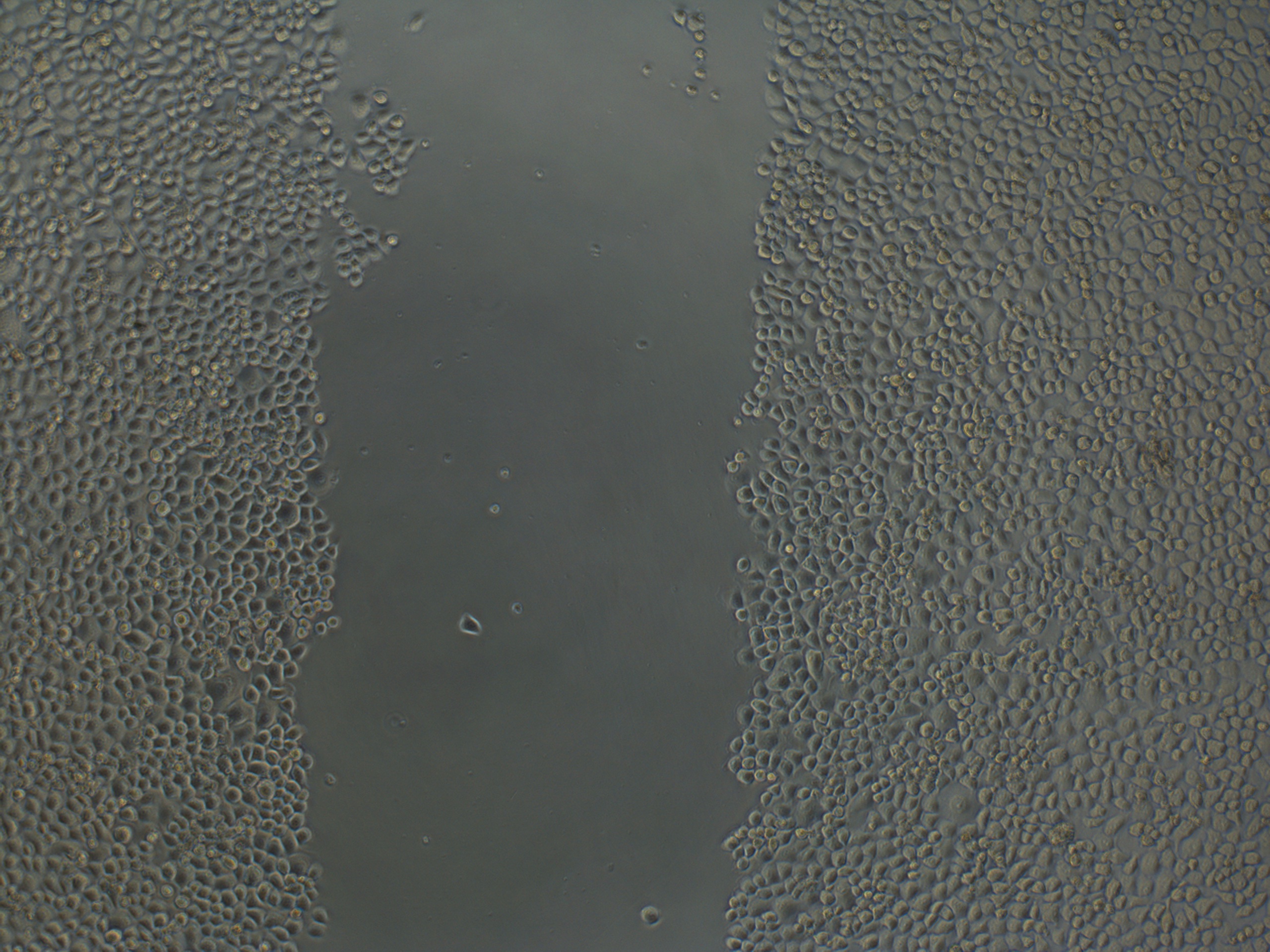

Supplement: Supplemental Information 5 [file peerj-11-15373-s005.zip › Raw data-Figure 5A-B-images-SMMC-7721/shFBXO43/0h/3.jpg]

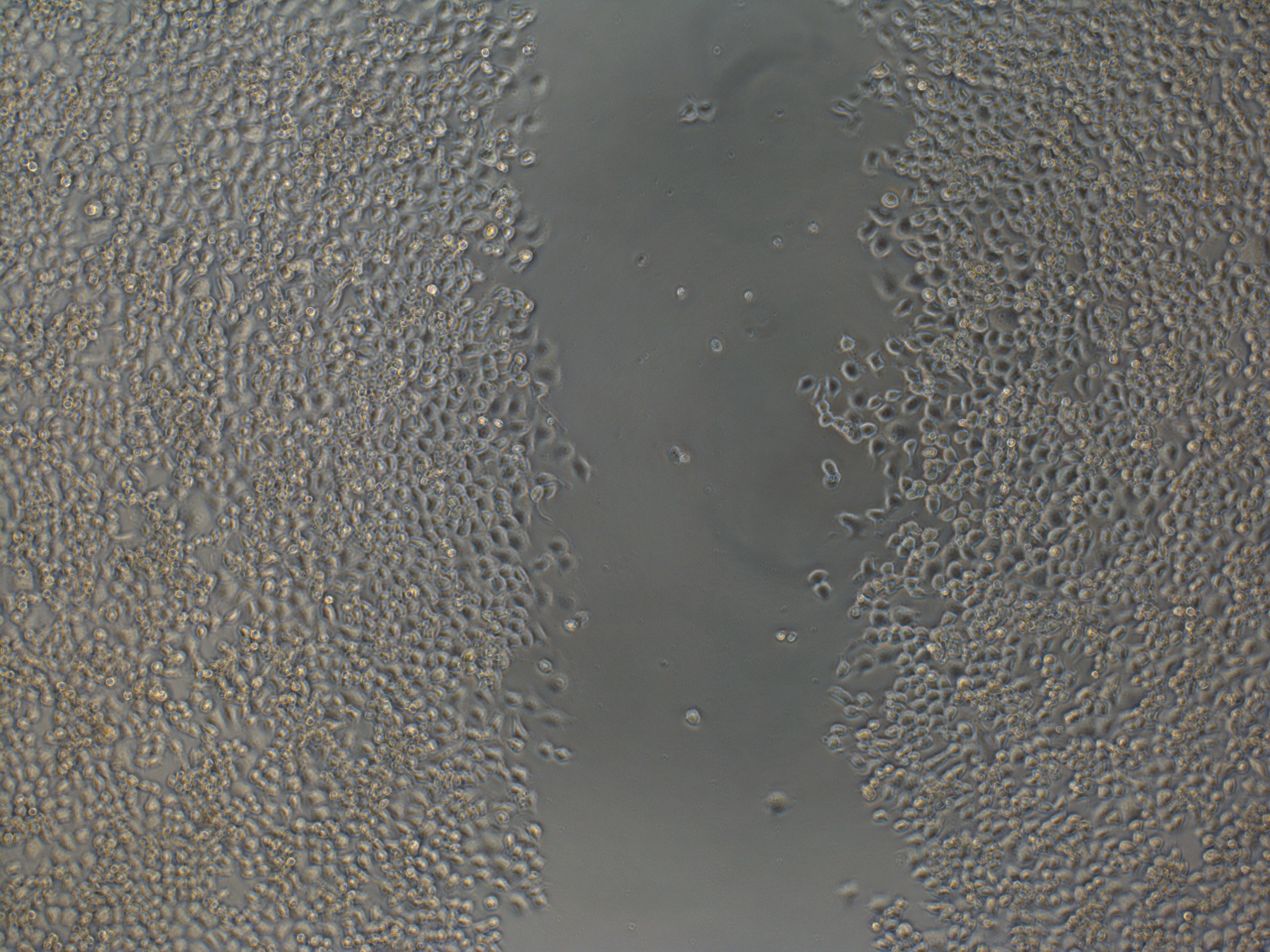

Supplement: Supplemental Information 5 [file peerj-11-15373-s005.zip › Raw data-Figure 5A-B-images-SMMC-7721/shFBXO43/24h/1.jpg]

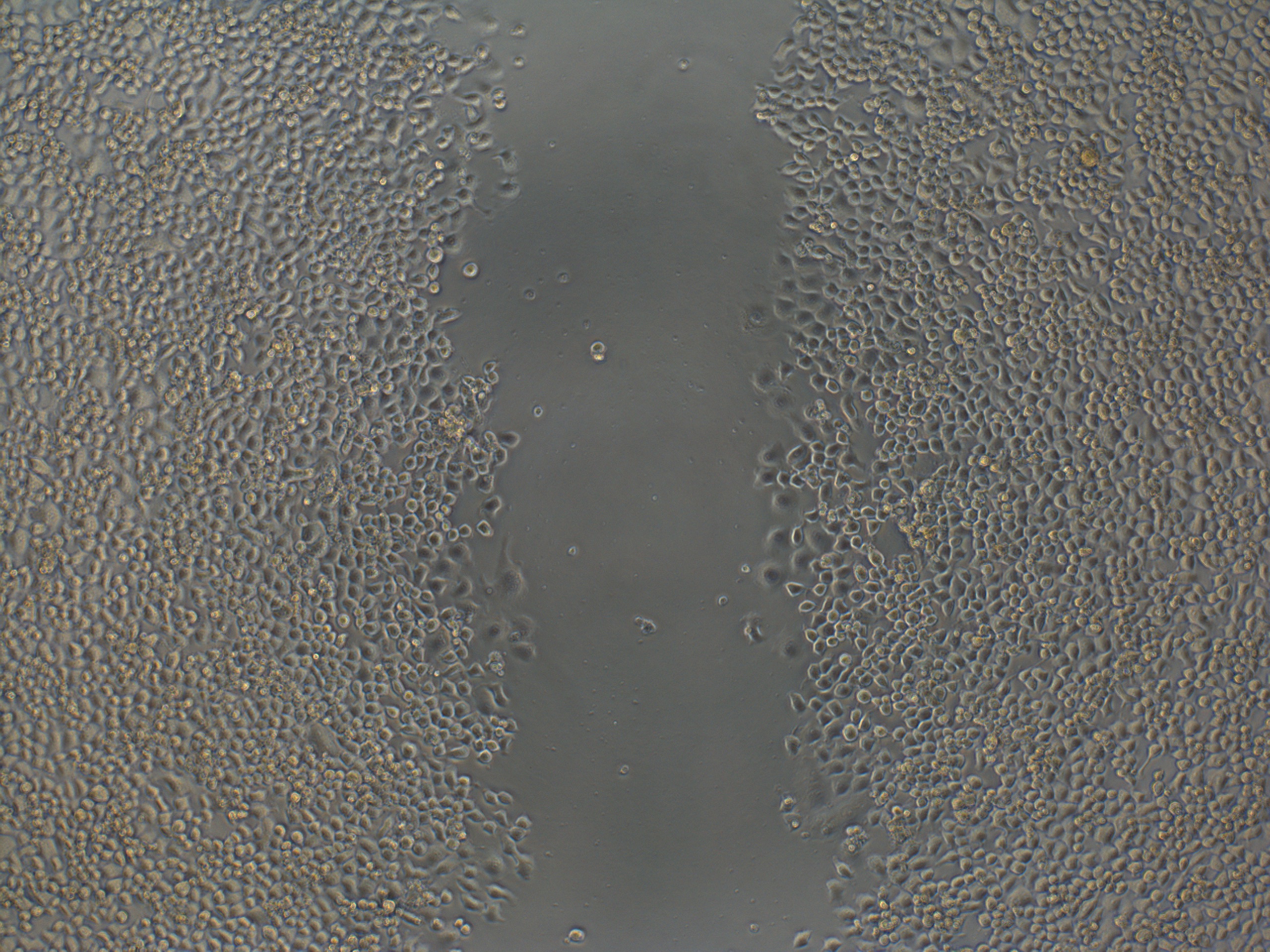

Supplement: Supplemental Information 5 [file peerj-11-15373-s005.zip › Raw data-Figure 5A-B-images-SMMC-7721/shFBXO43/24h/2.jpg]

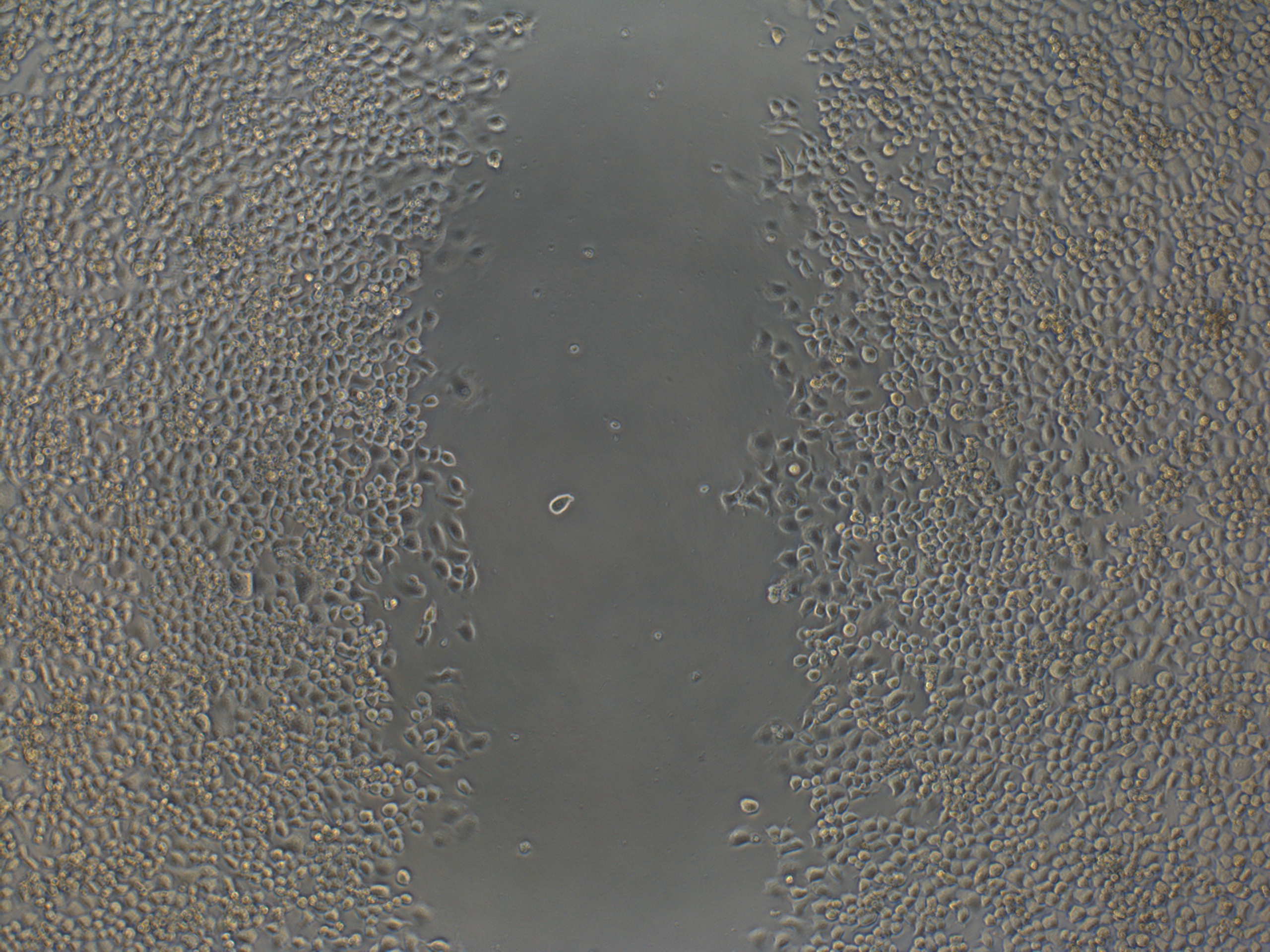

Supplement: Supplemental Information 5 [file peerj-11-15373-s005.zip › Raw data-Figure 5A-B-images-SMMC-7721/shFBXO43/24h/3.jpg]

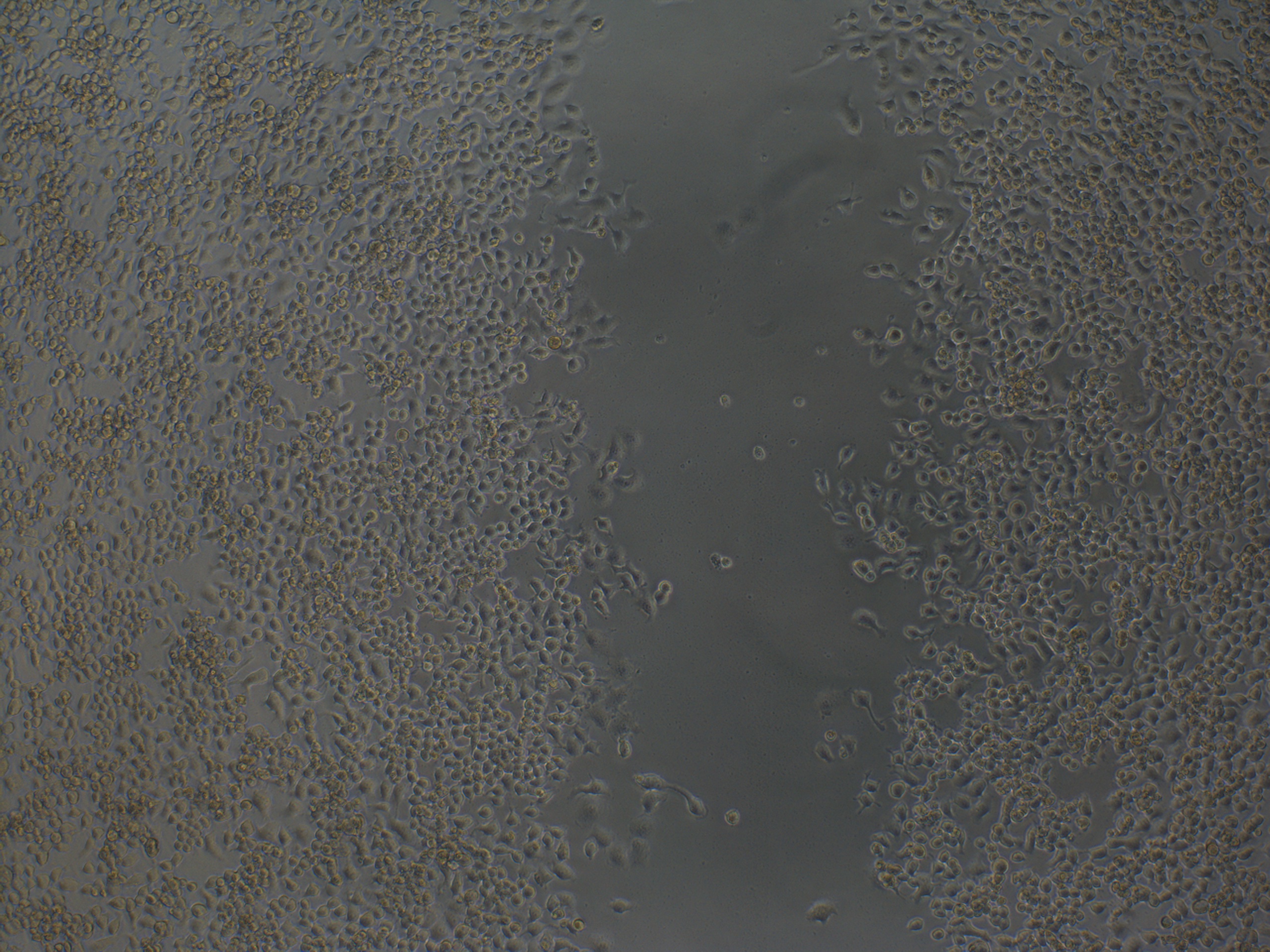

Supplement: Supplemental Information 5 [file peerj-11-15373-s005.zip › Raw data-Figure 5A-B-images-SMMC-7721/shFBXO43/48h/1.jpg]

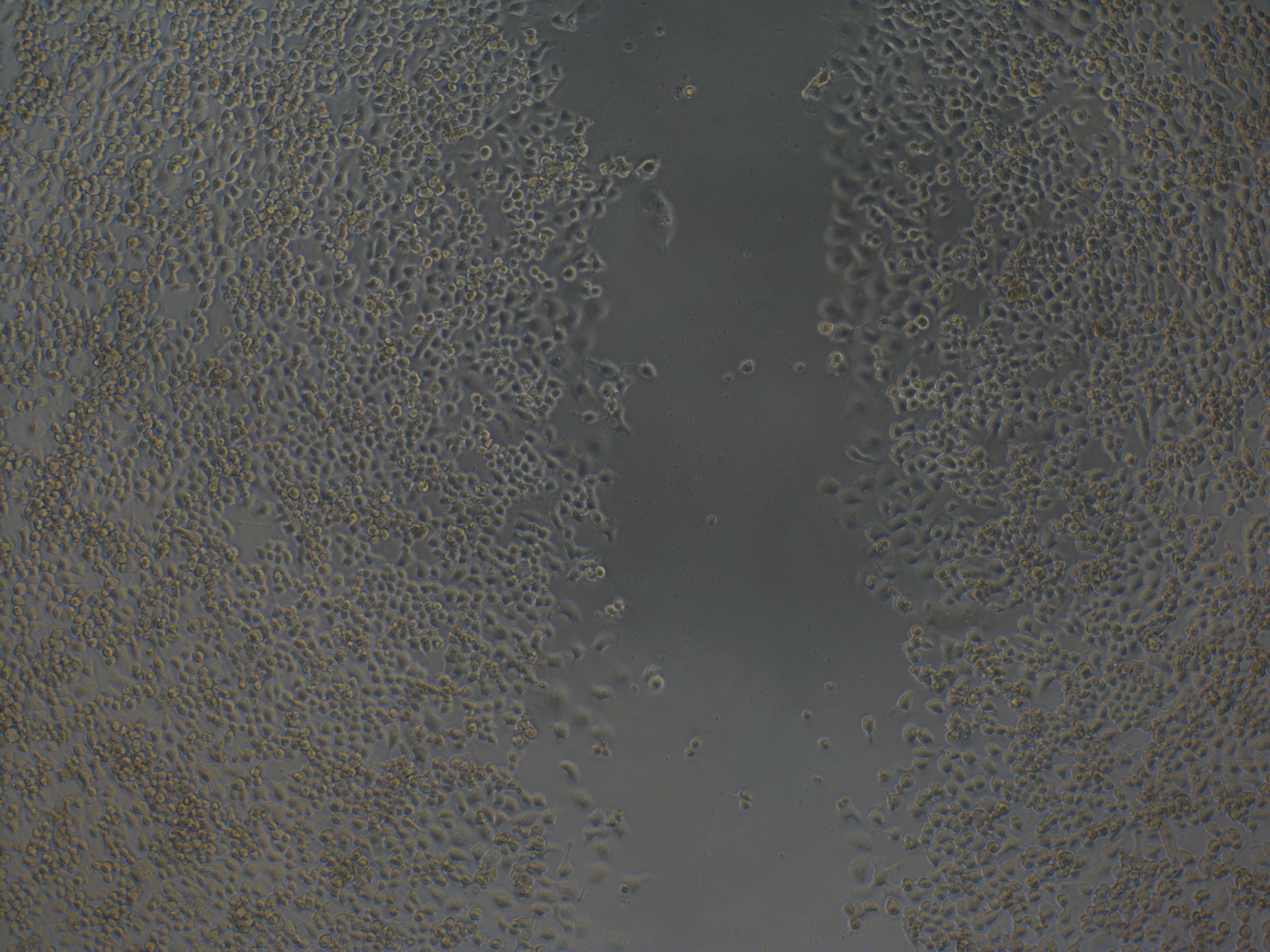

Supplement: Supplemental Information 5 [file peerj-11-15373-s005.zip › Raw data-Figure 5A-B-images-SMMC-7721/shFBXO43/48h/2.jpg]

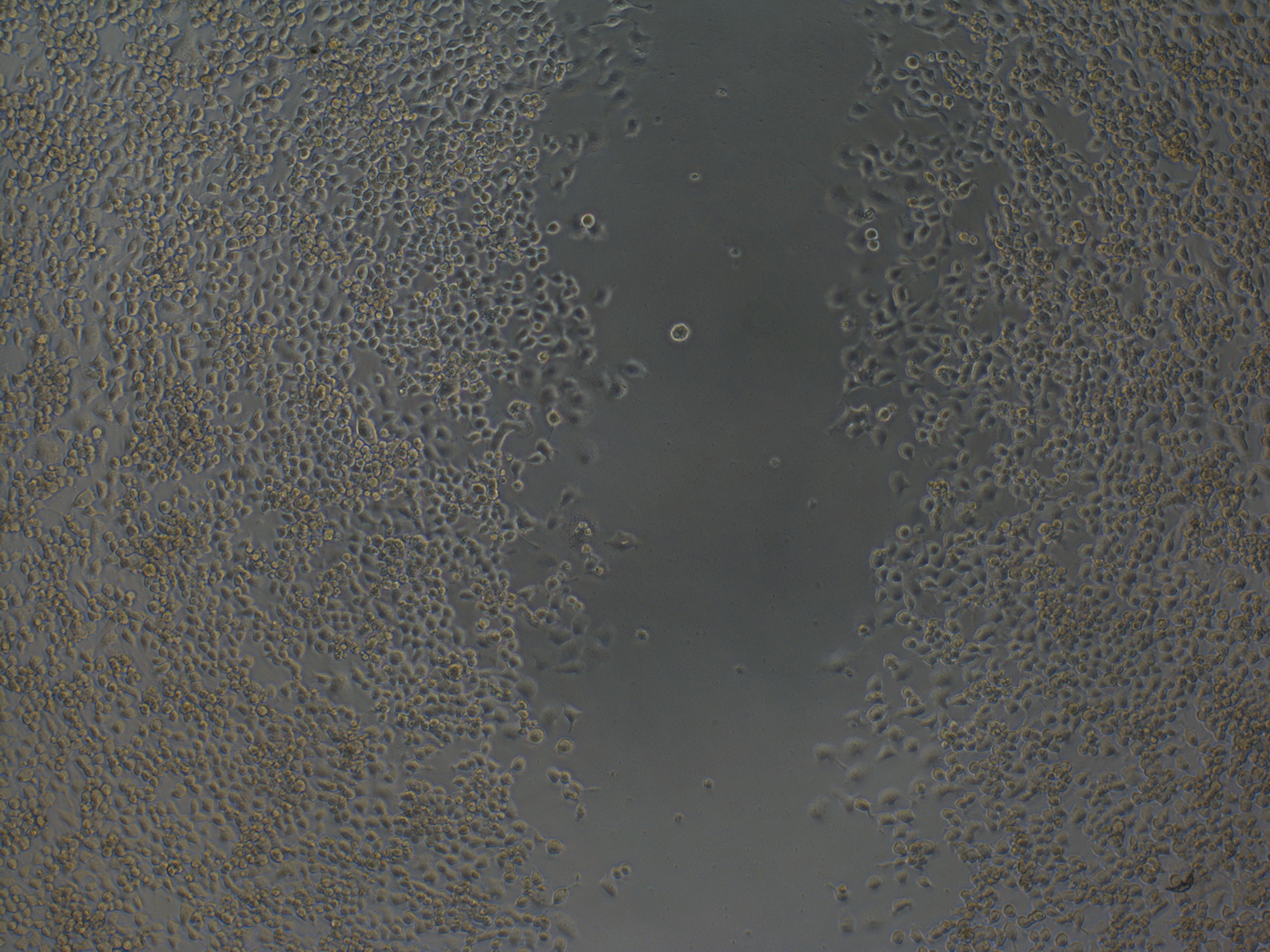

Supplement: Supplemental Information 5 [file peerj-11-15373-s005.zip › Raw data-Figure 5A-B-images-SMMC-7721/shFBXO43/48h/3.jpg]

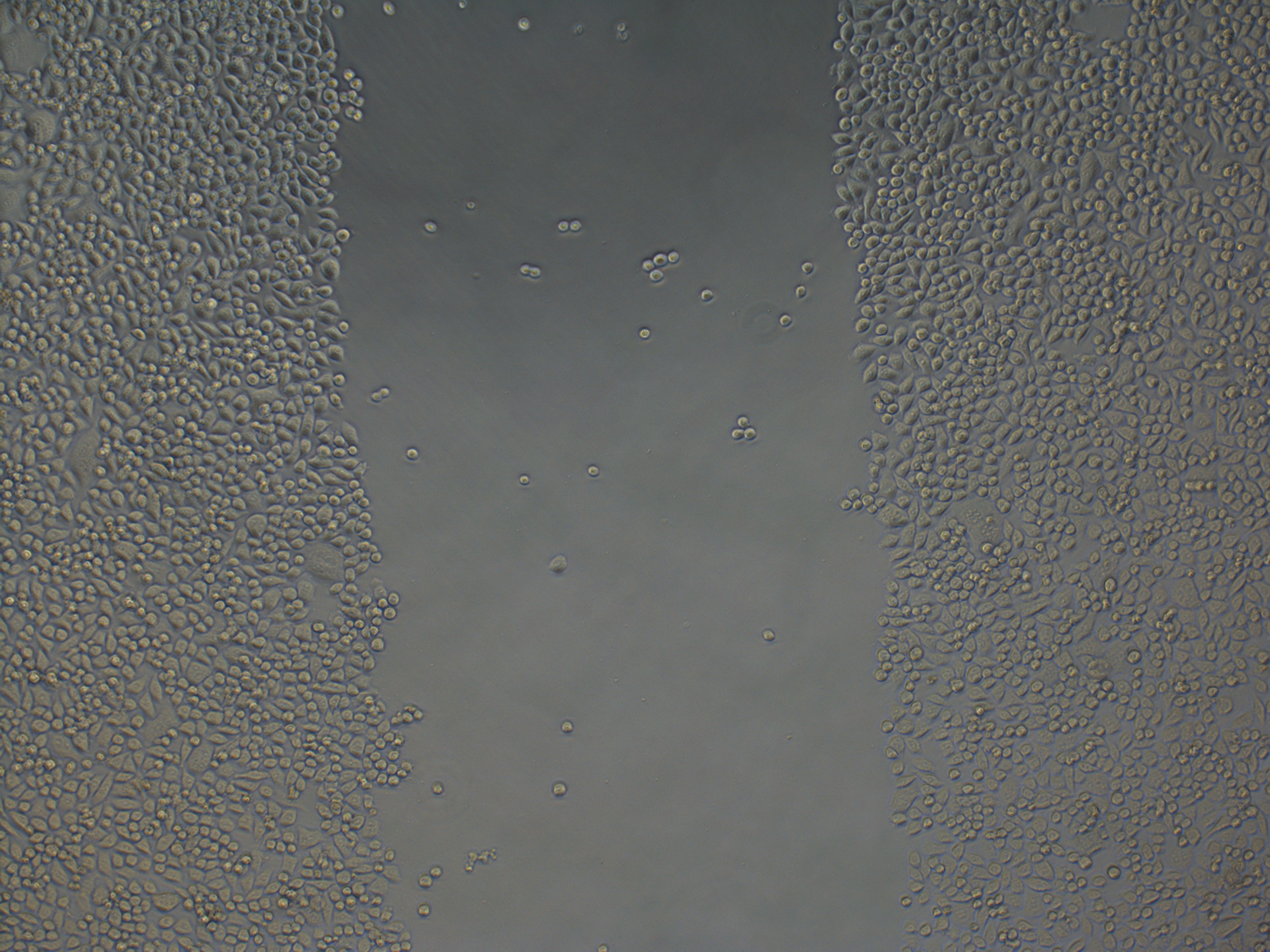

Supplement: Supplemental Information 6 [file peerj-11-15373-s006.zip › Raw data-Figure 5A-B-images-BEL-7404/shCtrl/0h/1.jpg]

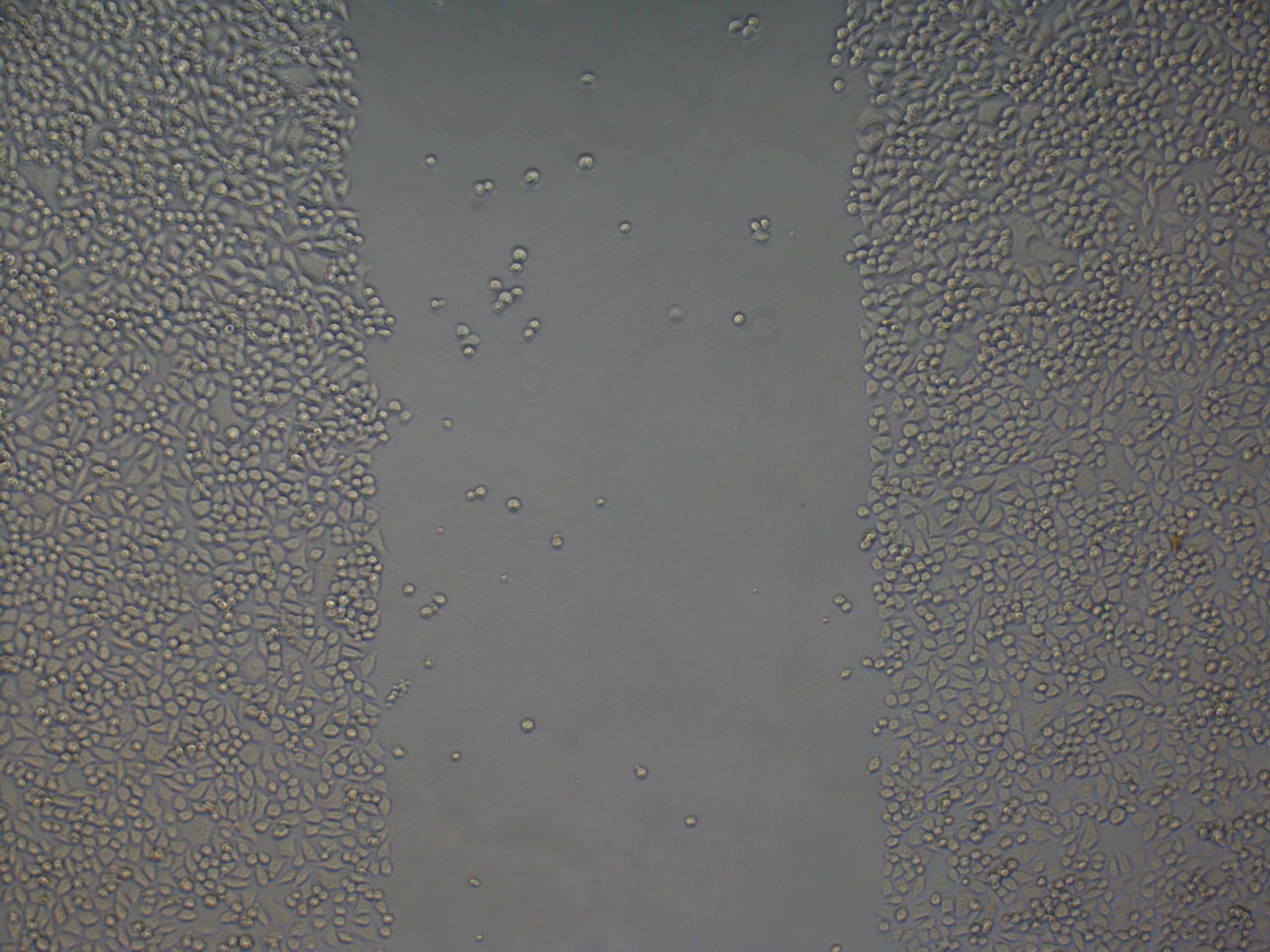

Supplement: Supplemental Information 6 [file peerj-11-15373-s006.zip › Raw data-Figure 5A-B-images-BEL-7404/shCtrl/0h/2.jpg]

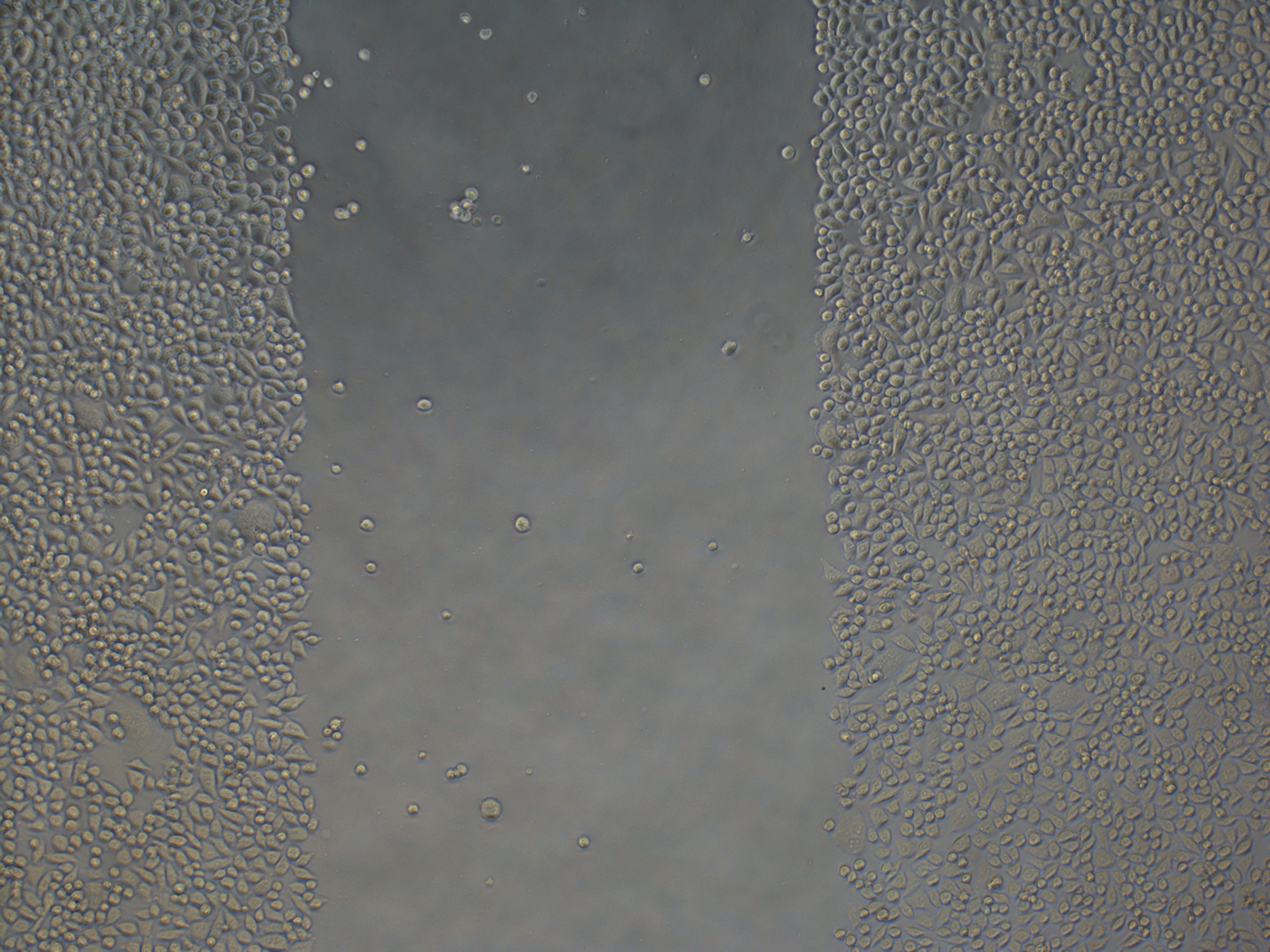

Supplement: Supplemental Information 6 [file peerj-11-15373-s006.zip › Raw data-Figure 5A-B-images-BEL-7404/shCtrl/0h/3.jpg]

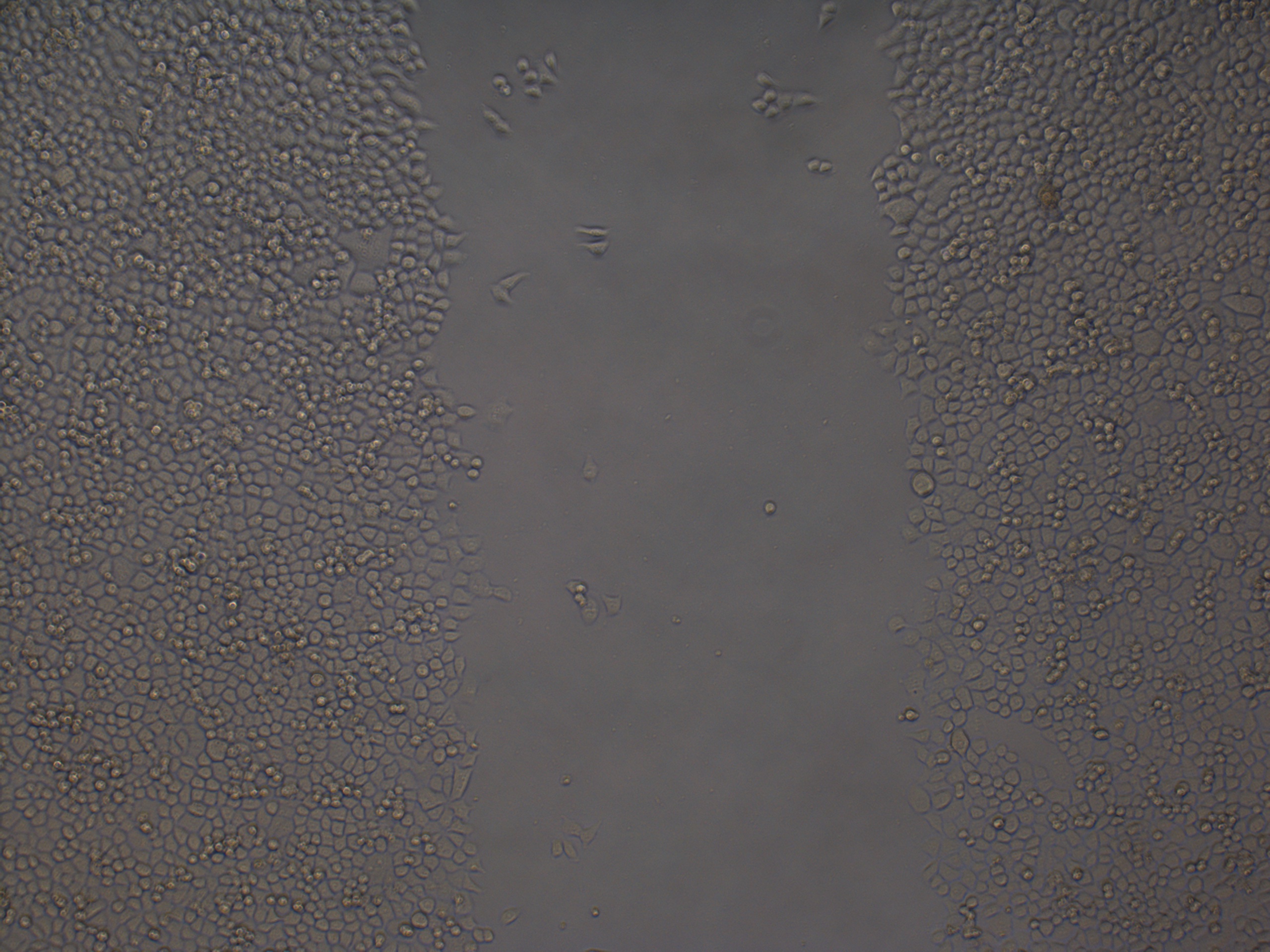

Supplement: Supplemental Information 6 [file peerj-11-15373-s006.zip › Raw data-Figure 5A-B-images-BEL-7404/shCtrl/24h/1.jpg]

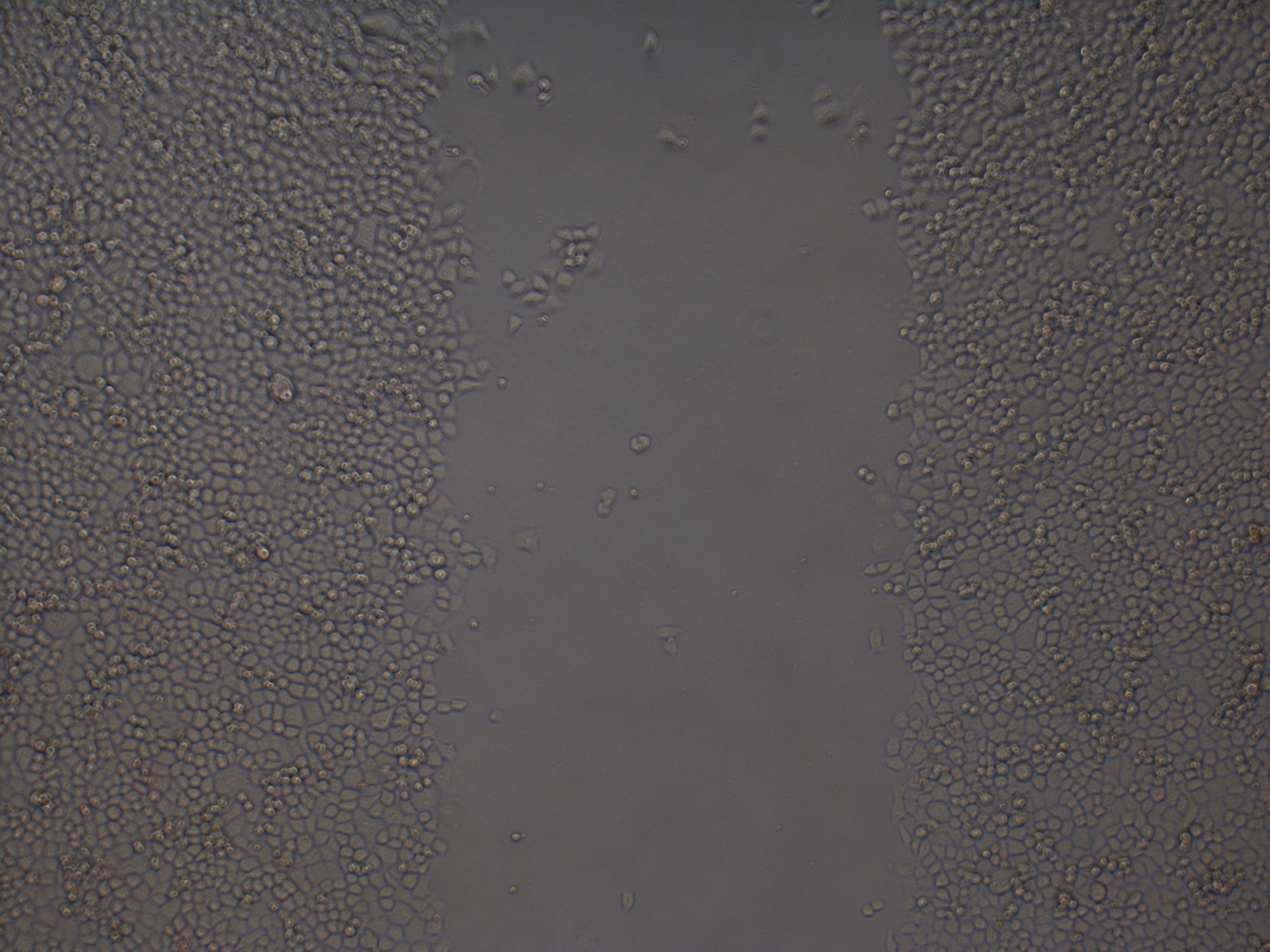

Supplement: Supplemental Information 6 [file peerj-11-15373-s006.zip › Raw data-Figure 5A-B-images-BEL-7404/shCtrl/24h/2.jpg]

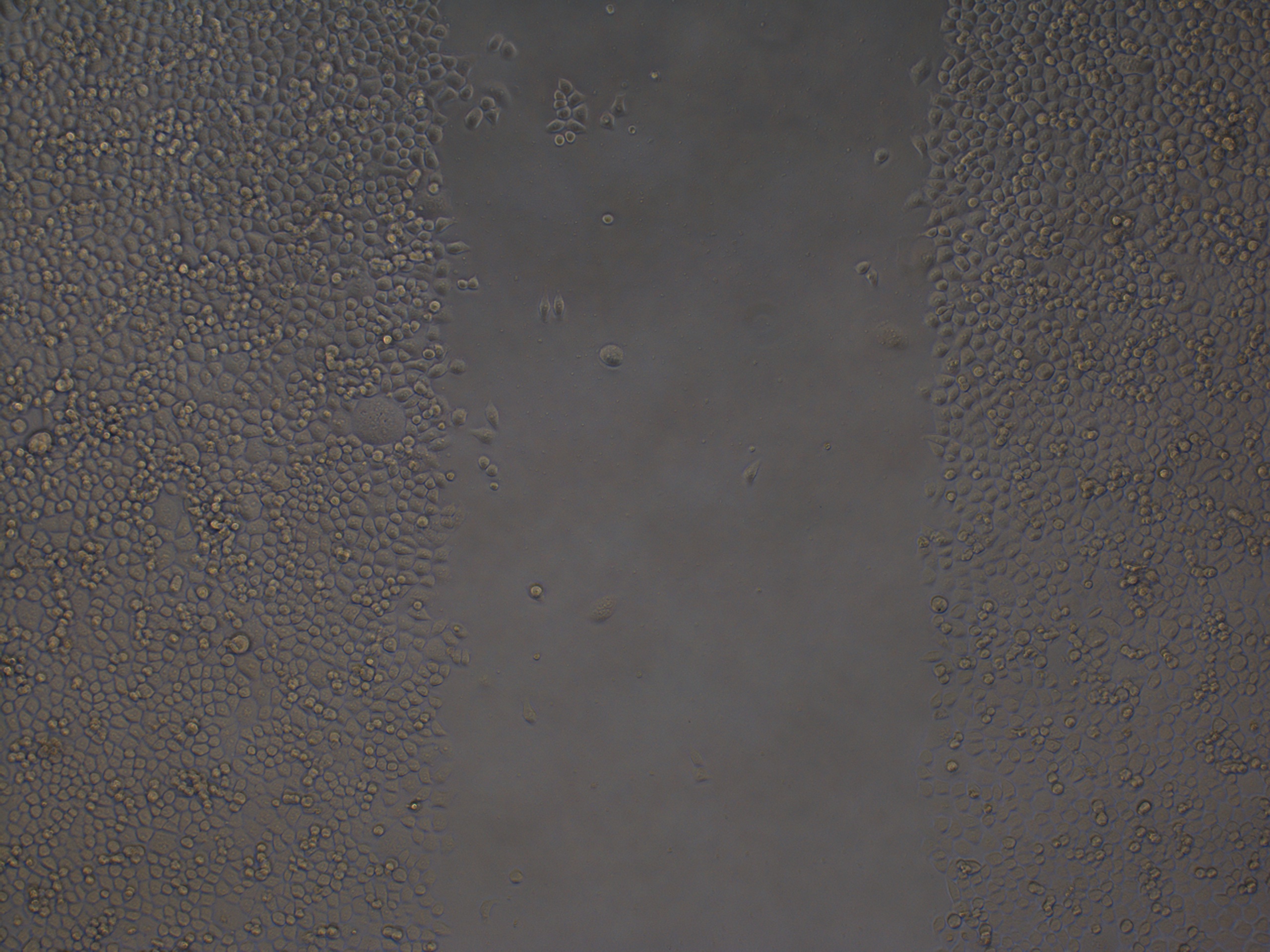

Supplement: Supplemental Information 6 [file peerj-11-15373-s006.zip › Raw data-Figure 5A-B-images-BEL-7404/shCtrl/24h/3.jpg]

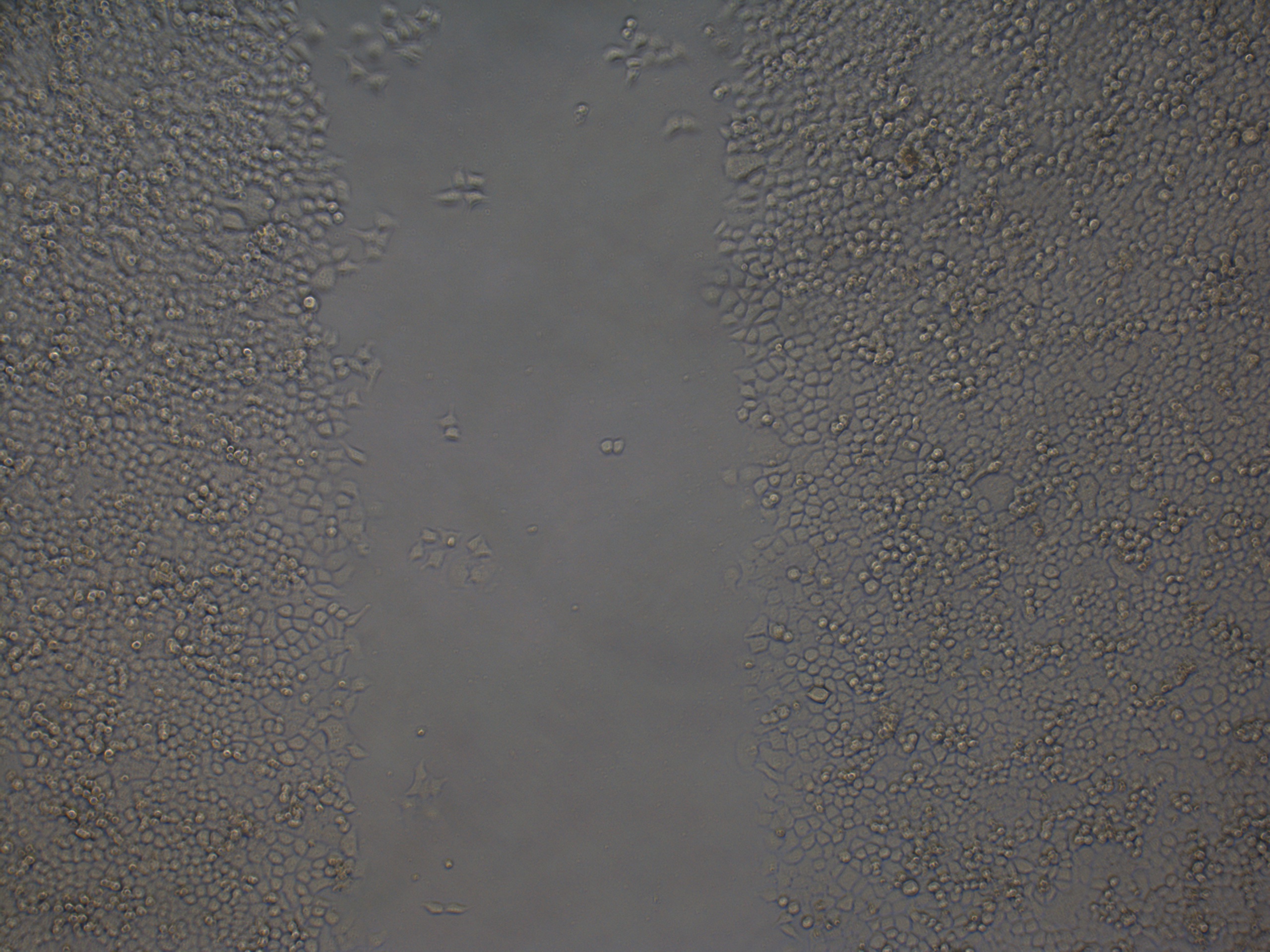

Supplement: Supplemental Information 6 [file peerj-11-15373-s006.zip › Raw data-Figure 5A-B-images-BEL-7404/shCtrl/48h/1.jpg]

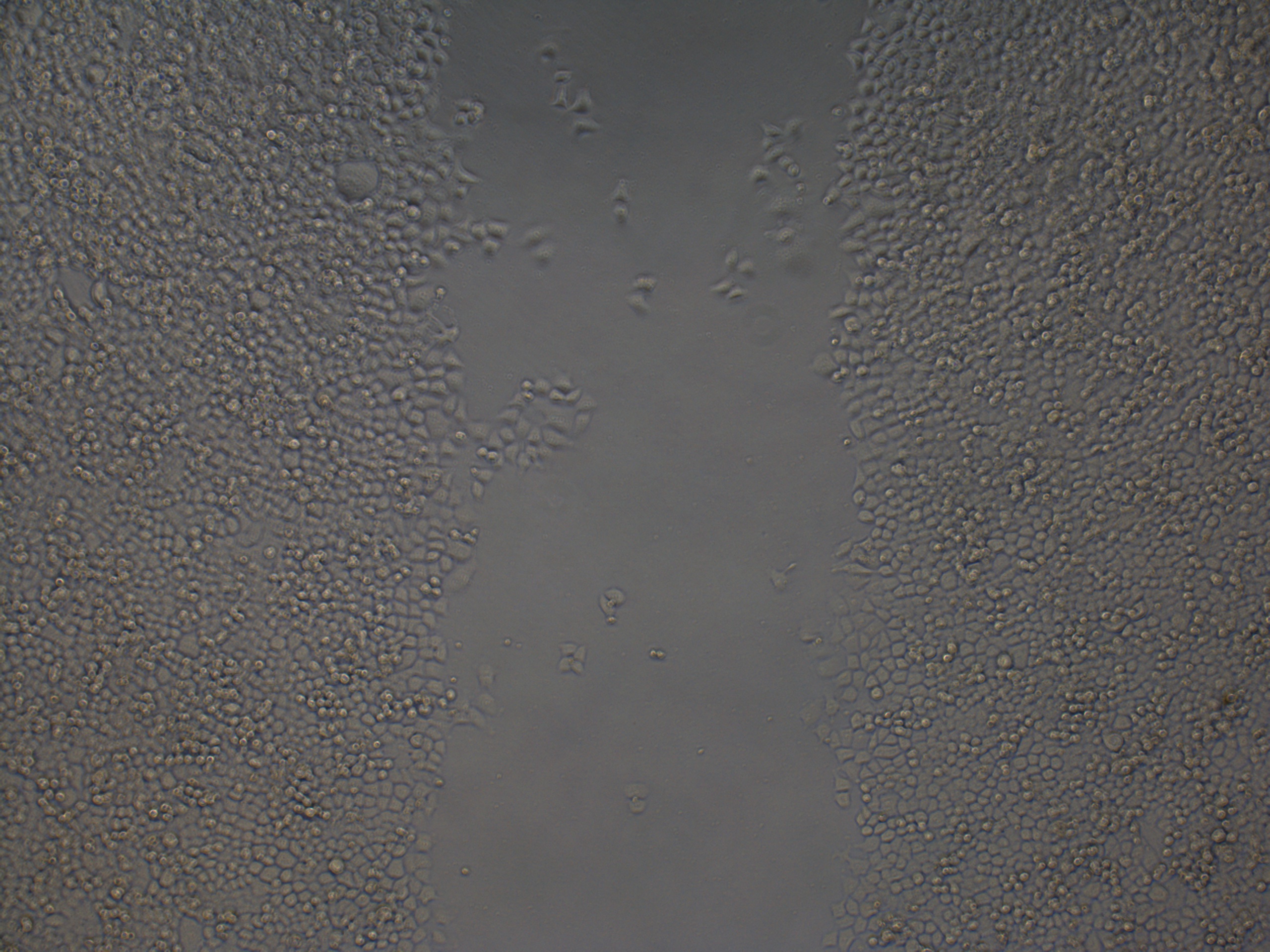

Supplement: Supplemental Information 6 [file peerj-11-15373-s006.zip › Raw data-Figure 5A-B-images-BEL-7404/shCtrl/48h/2.jpg]

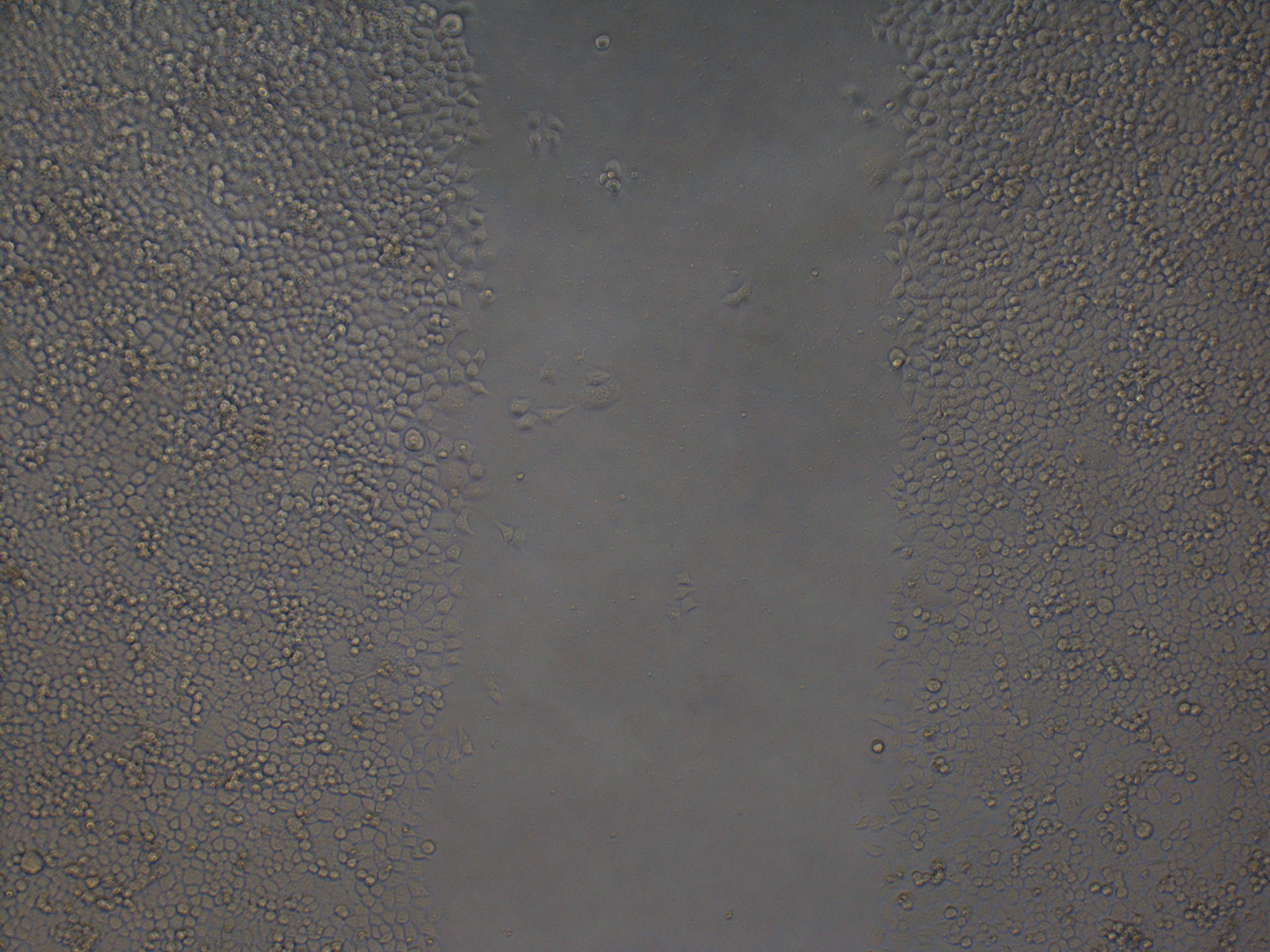

Supplement: Supplemental Information 6 [file peerj-11-15373-s006.zip › Raw data-Figure 5A-B-images-BEL-7404/shCtrl/48h/3.jpg]

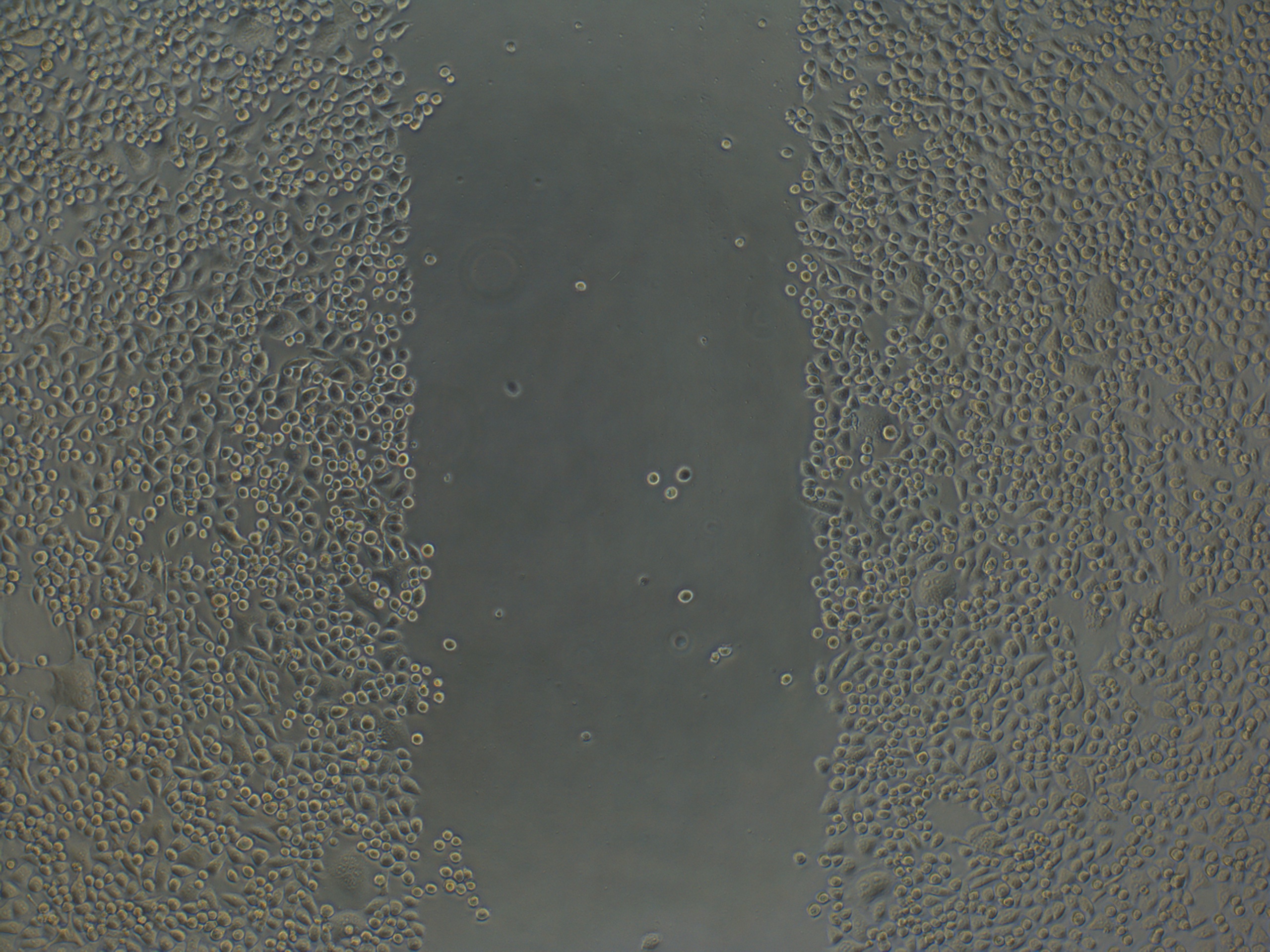

Supplement: Supplemental Information 6 [file peerj-11-15373-s006.zip › Raw data-Figure 5A-B-images-BEL-7404/shFBXO43/0h/1.jpg]

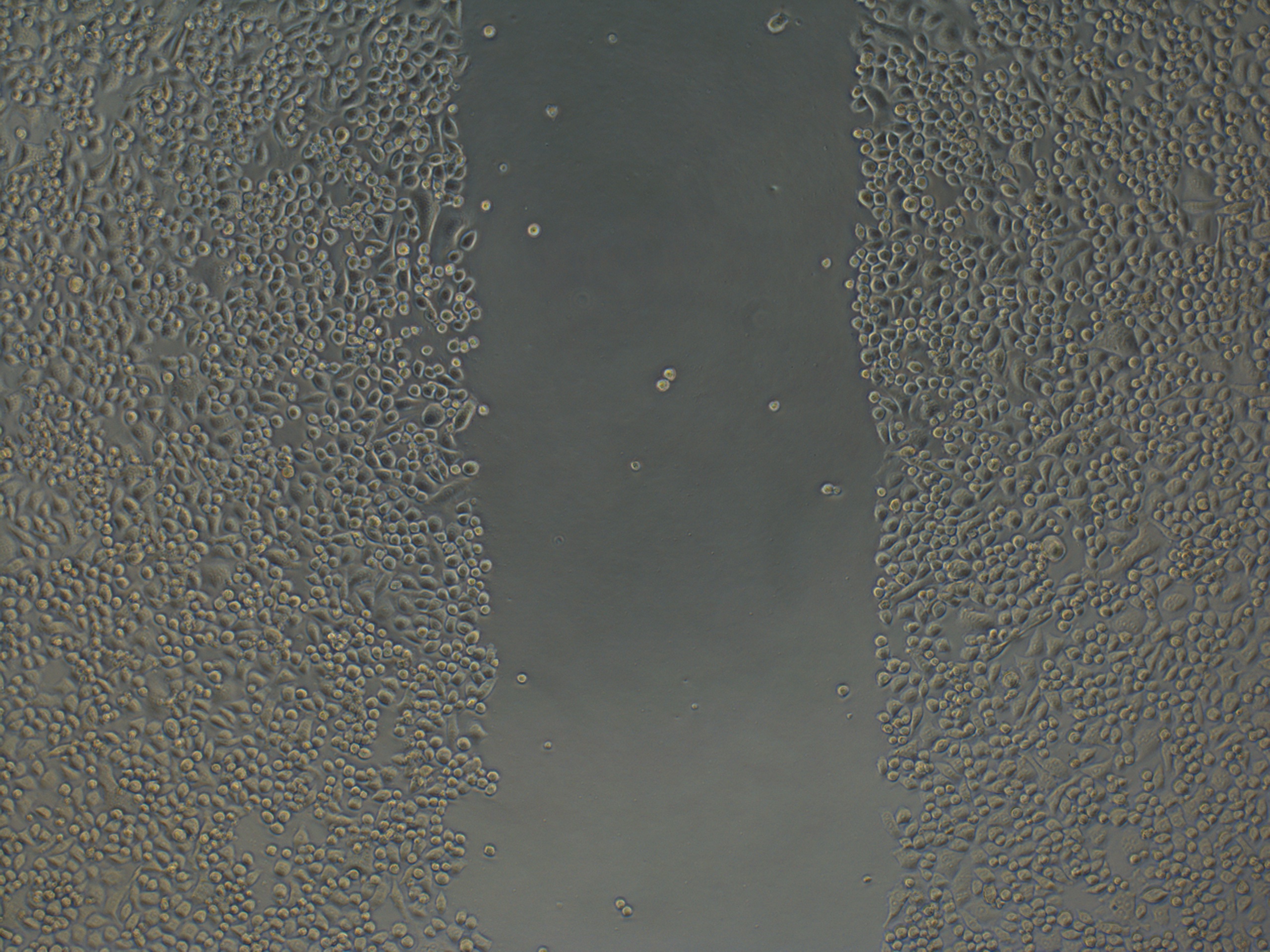

Supplement: Supplemental Information 6 [file peerj-11-15373-s006.zip › Raw data-Figure 5A-B-images-BEL-7404/shFBXO43/0h/2.jpg]

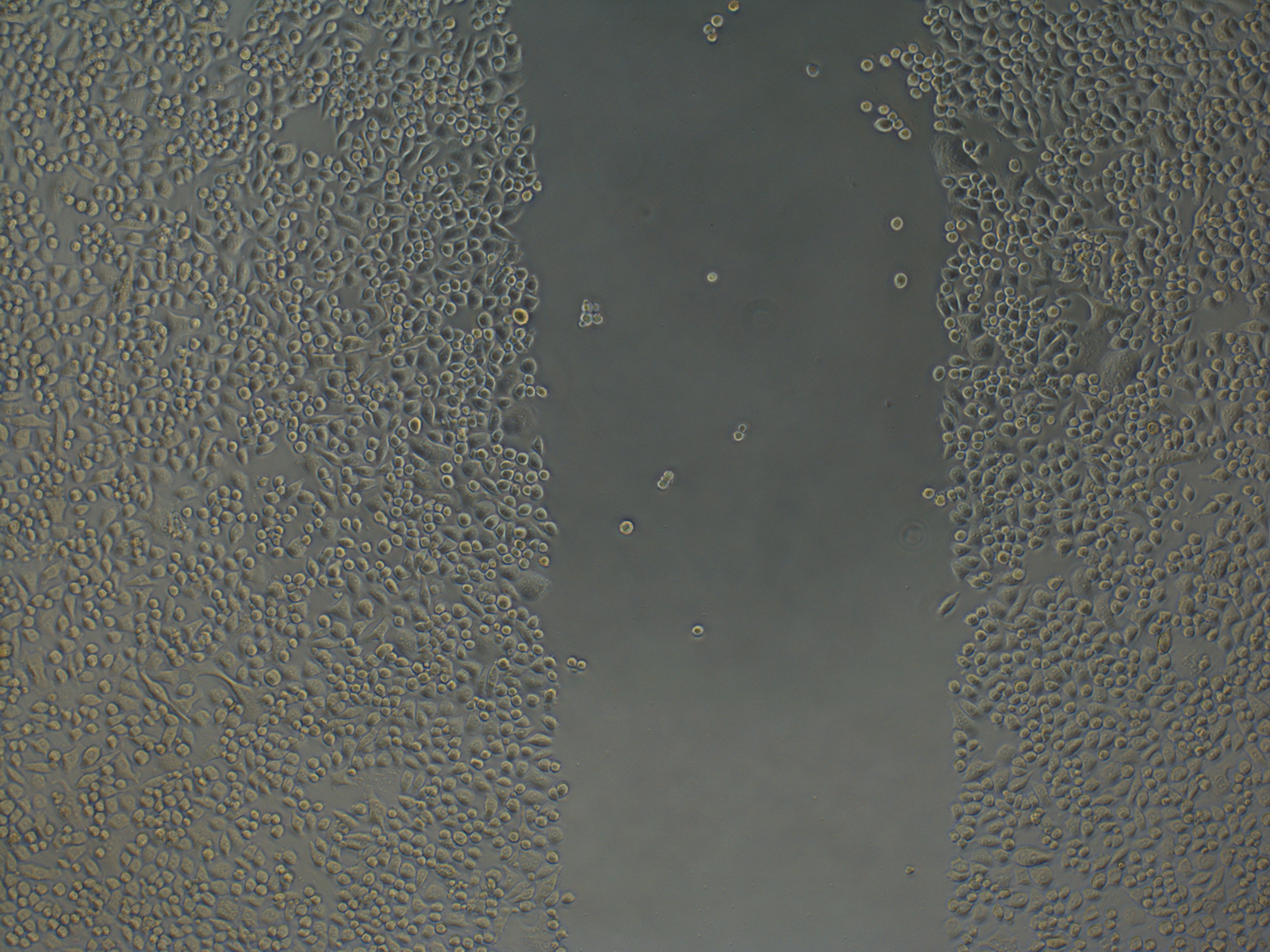

Supplement: Supplemental Information 6 [file peerj-11-15373-s006.zip › Raw data-Figure 5A-B-images-BEL-7404/shFBXO43/0h/3.jpg]

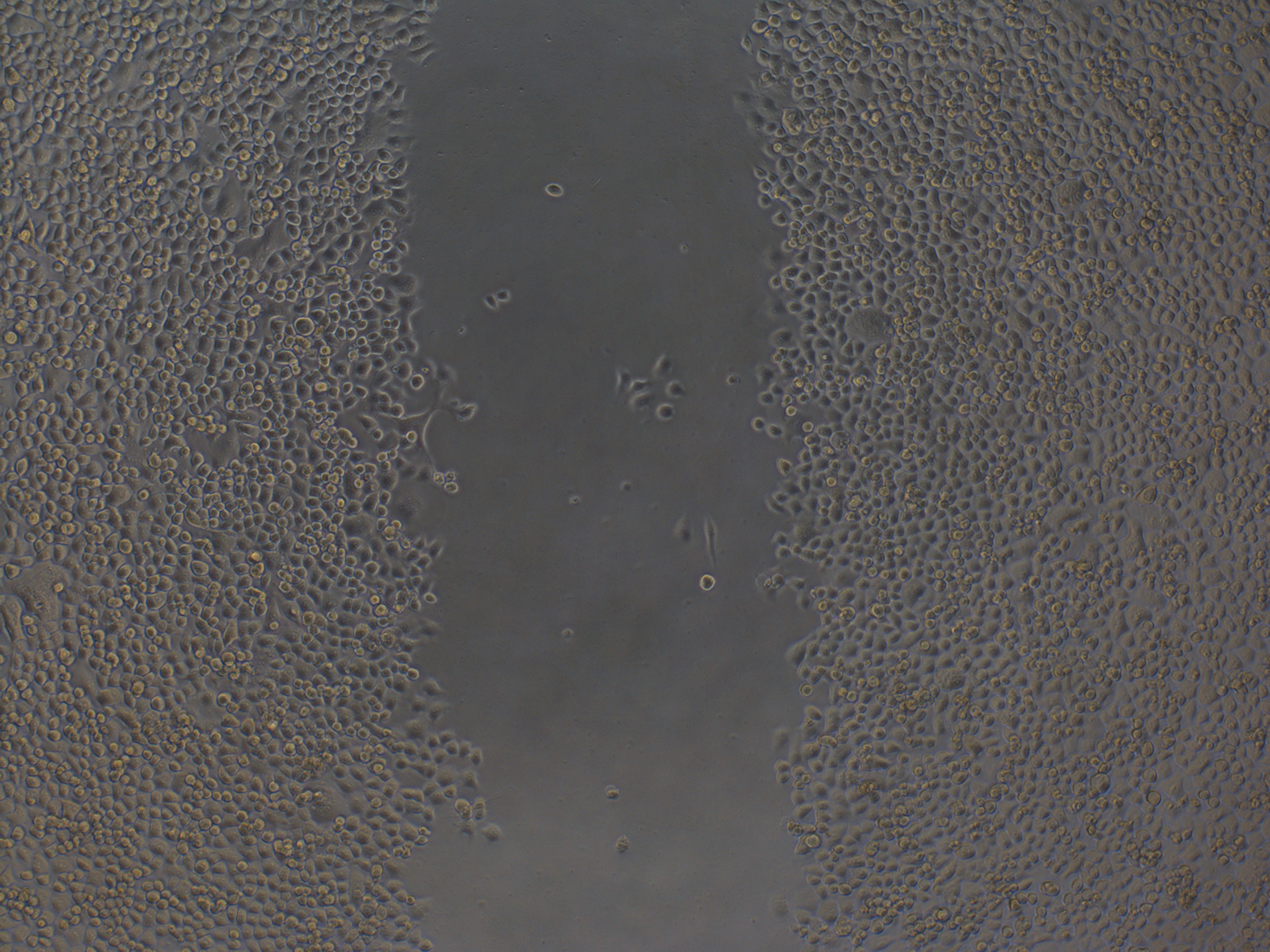

Supplement: Supplemental Information 6 [file peerj-11-15373-s006.zip › Raw data-Figure 5A-B-images-BEL-7404/shFBXO43/24h/1.jpg]

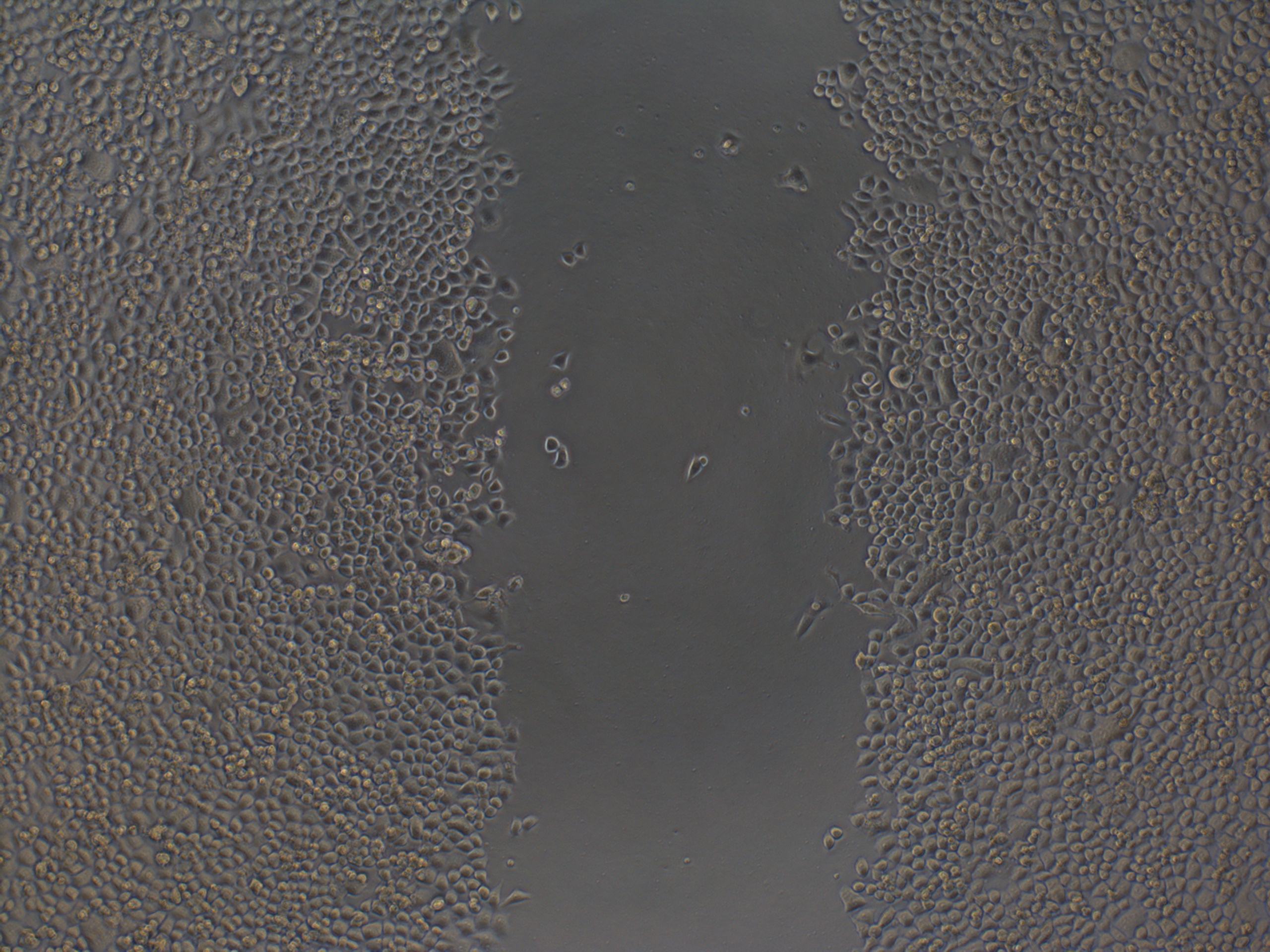

Supplement: Supplemental Information 6 [file peerj-11-15373-s006.zip › Raw data-Figure 5A-B-images-BEL-7404/shFBXO43/24h/2.jpg]

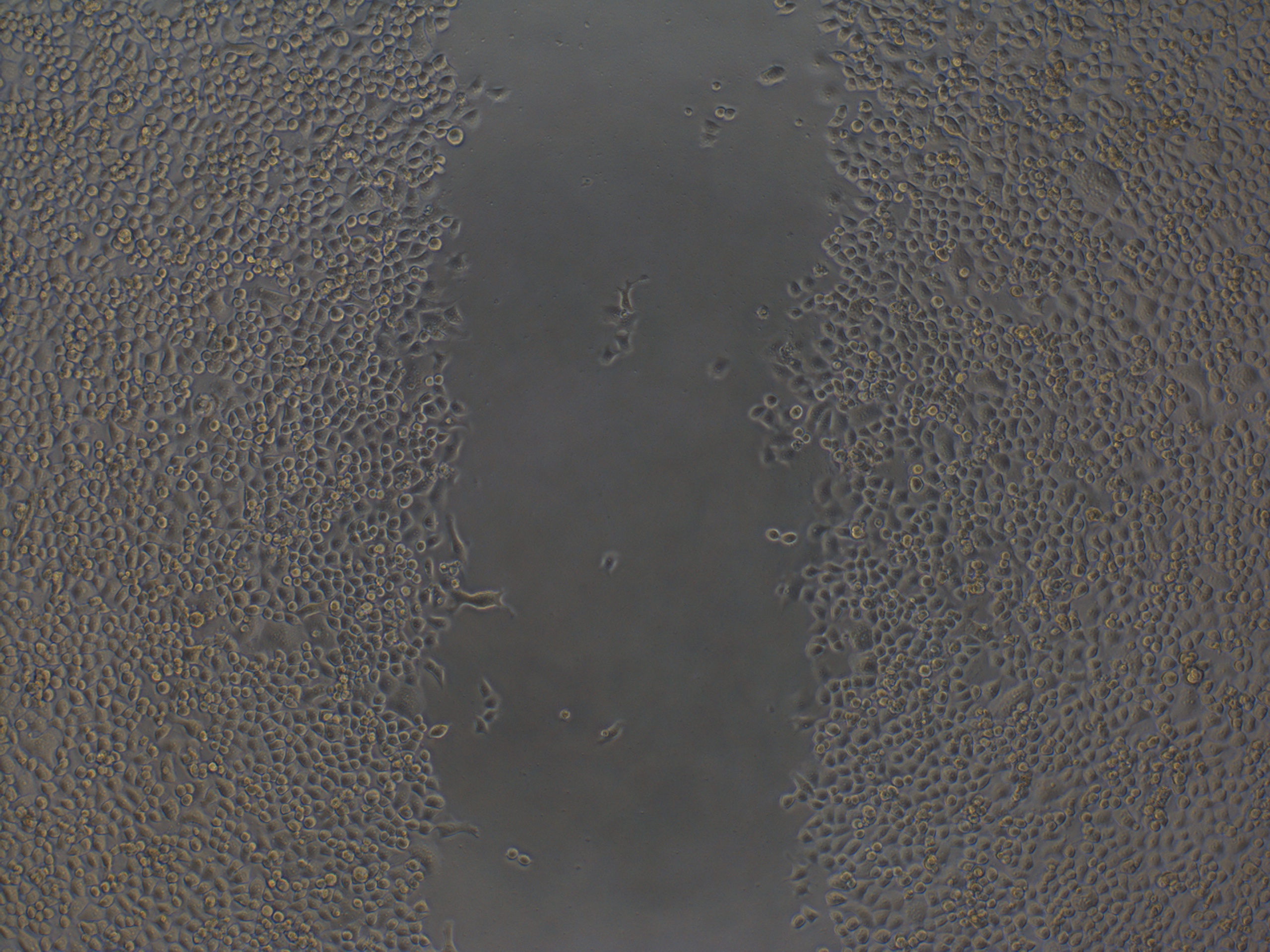

Supplement: Supplemental Information 6 [file peerj-11-15373-s006.zip › Raw data-Figure 5A-B-images-BEL-7404/shFBXO43/24h/3.jpg]

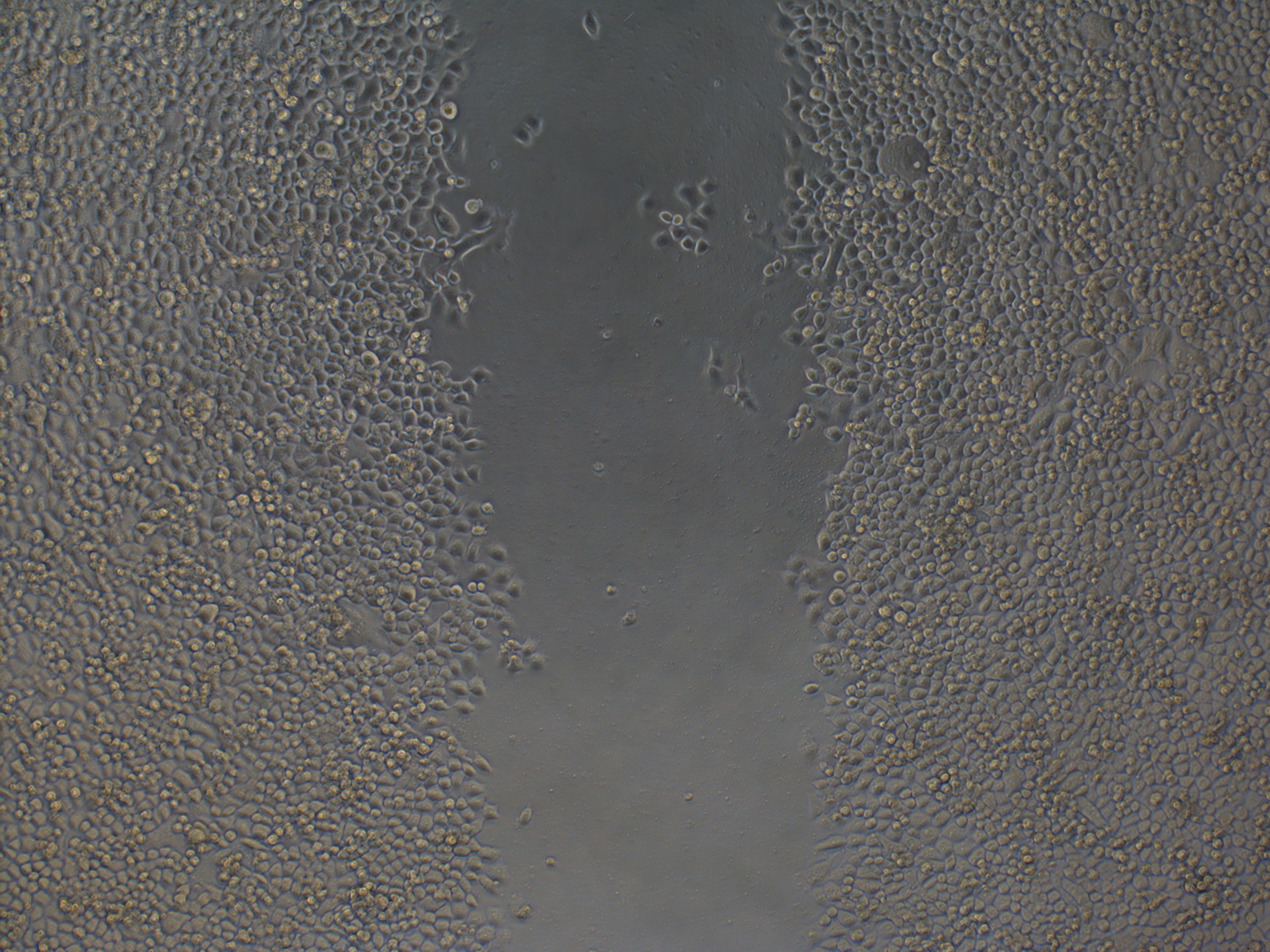

Supplement: Supplemental Information 6 [file peerj-11-15373-s006.zip › Raw data-Figure 5A-B-images-BEL-7404/shFBXO43/48h/1.jpg]

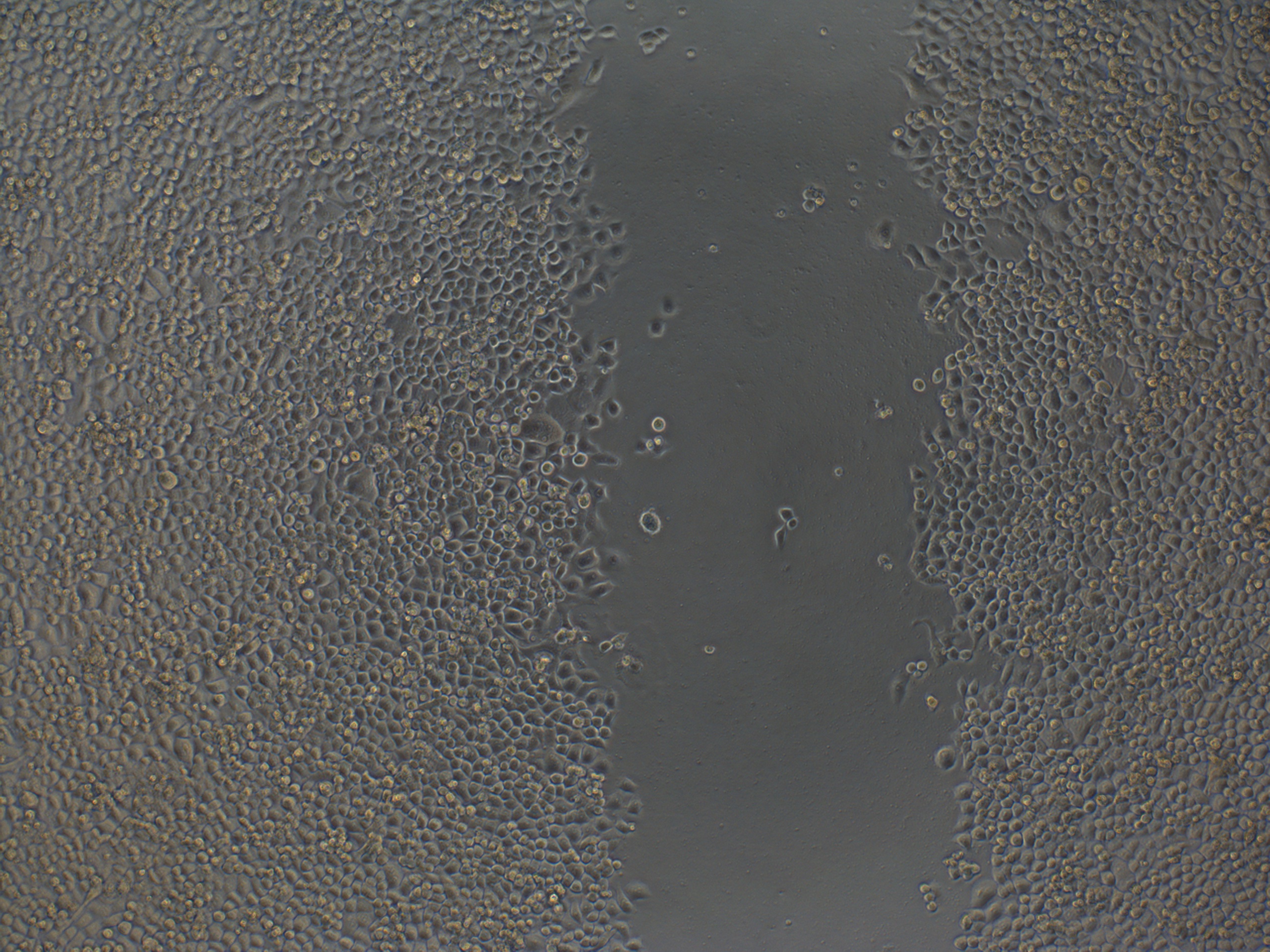

Supplement: Supplemental Information 6 [file peerj-11-15373-s006.zip › Raw data-Figure 5A-B-images-BEL-7404/shFBXO43/48h/2.jpg]

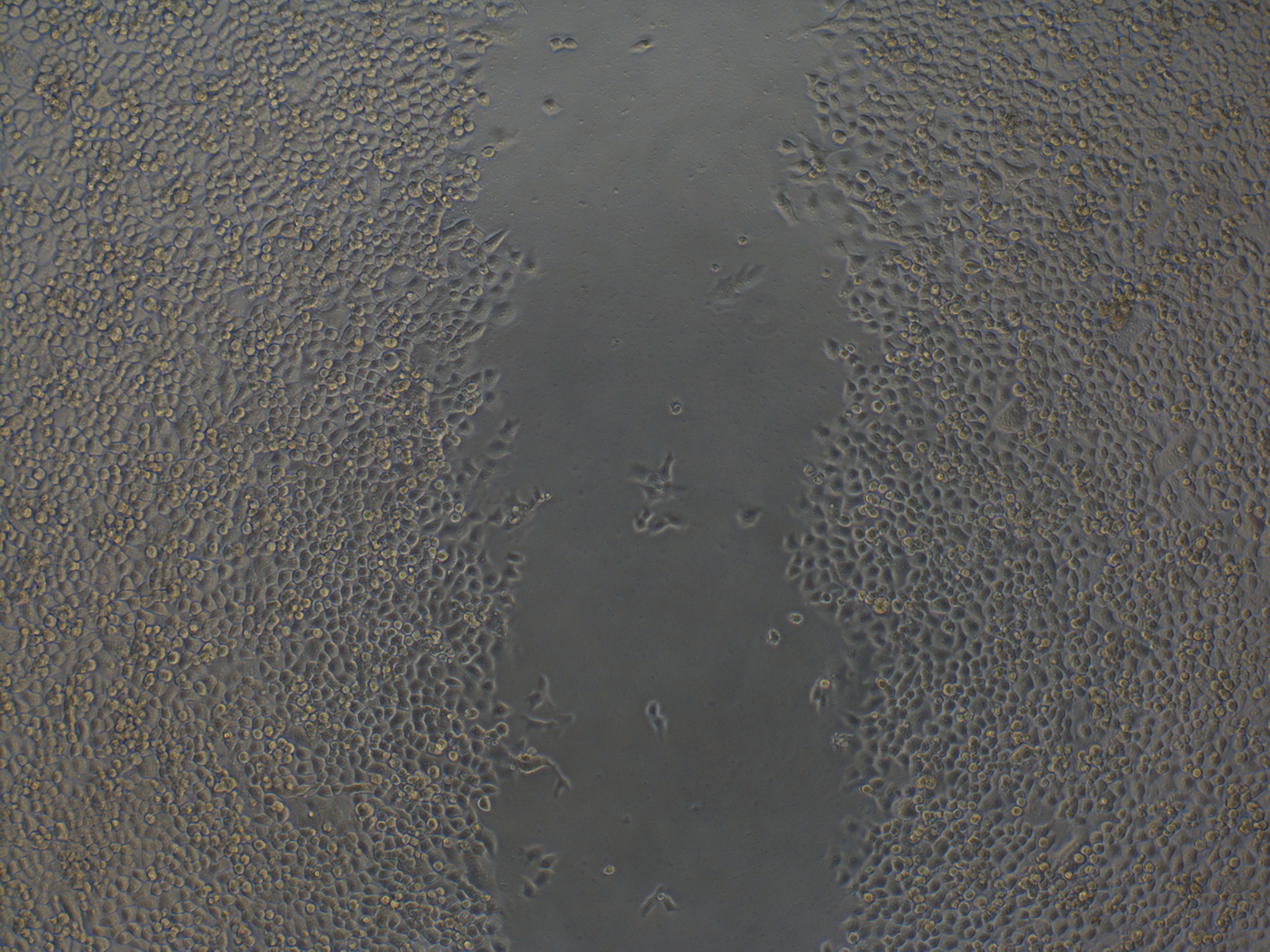

Supplement: Supplemental Information 6 [file peerj-11-15373-s006.zip › Raw data-Figure 5A-B-images-BEL-7404/shFBXO43/48h/3.jpg]

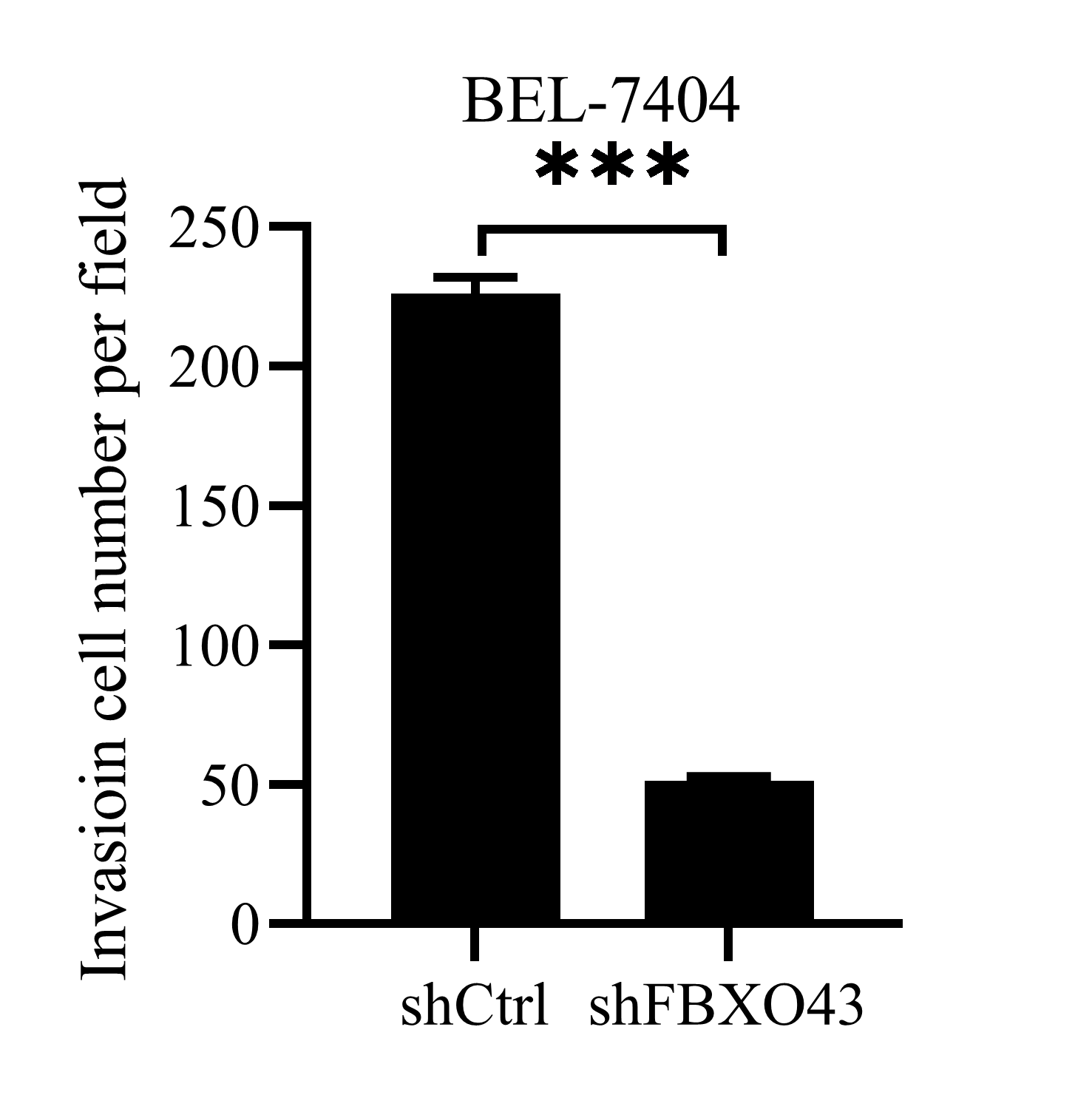

Supplement: Supplemental Information 7 [file peerj-11-15373-s007.zip › Figure 5C-D/BEL-7404.png]

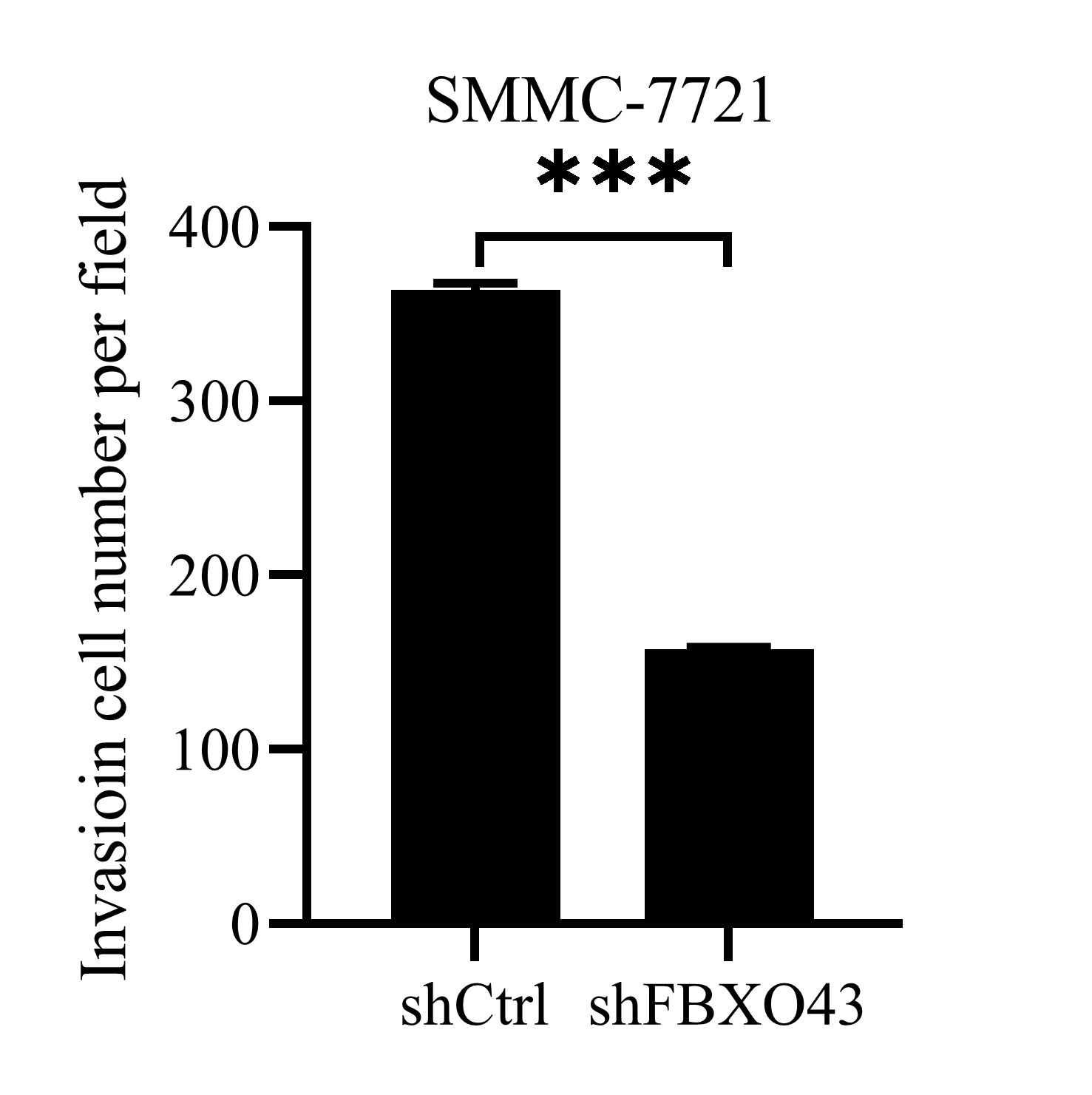

Supplement: Supplemental Information 7 [file peerj-11-15373-s007.zip › Figure 5C-D/SMMC-7721.png]

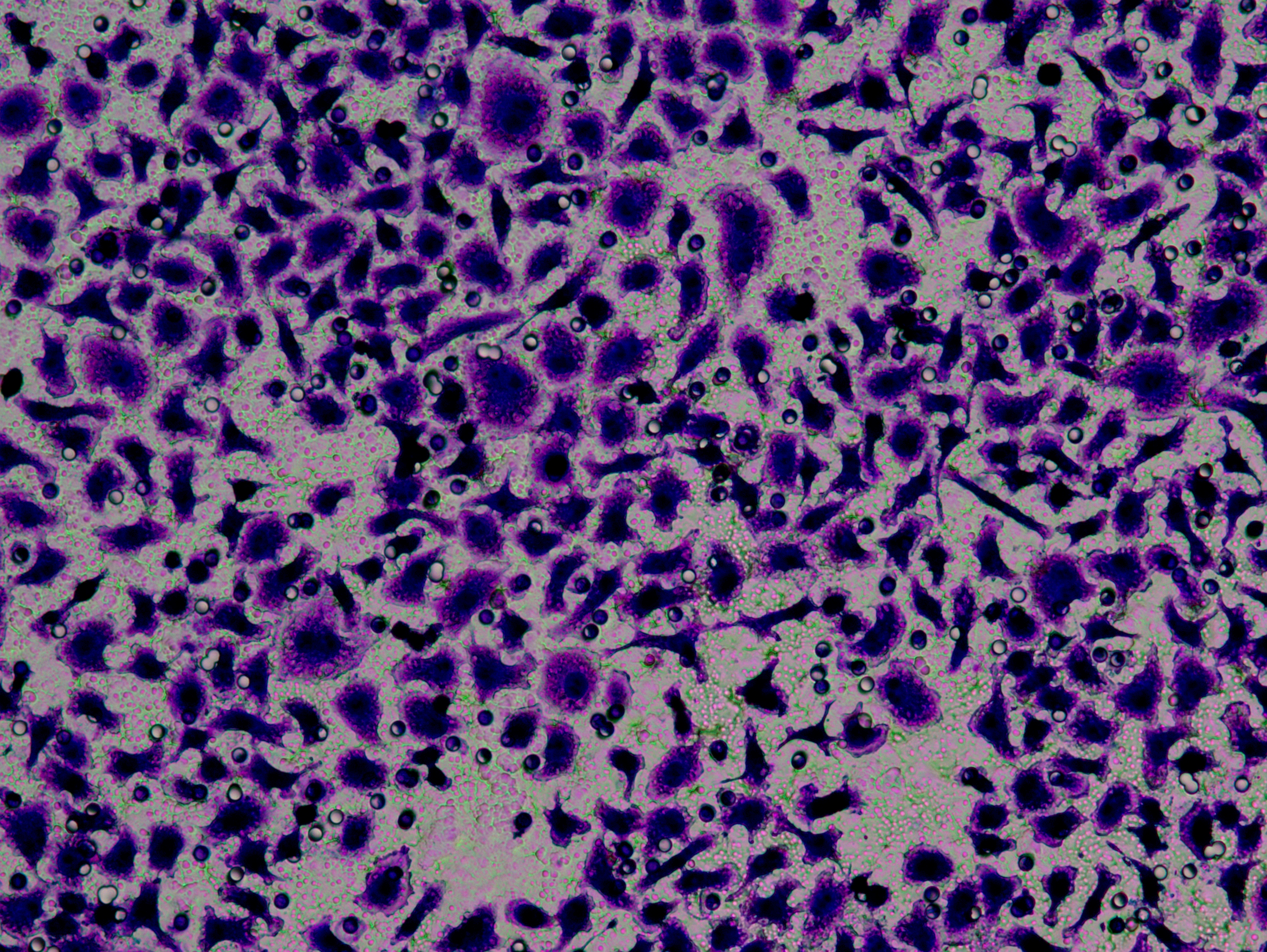

Supplement: Supplemental Information 8 [file peerj-11-15373-s008.zip › Figure 5C-D-images200X-SMMC-7721/shCtrl.jpg]

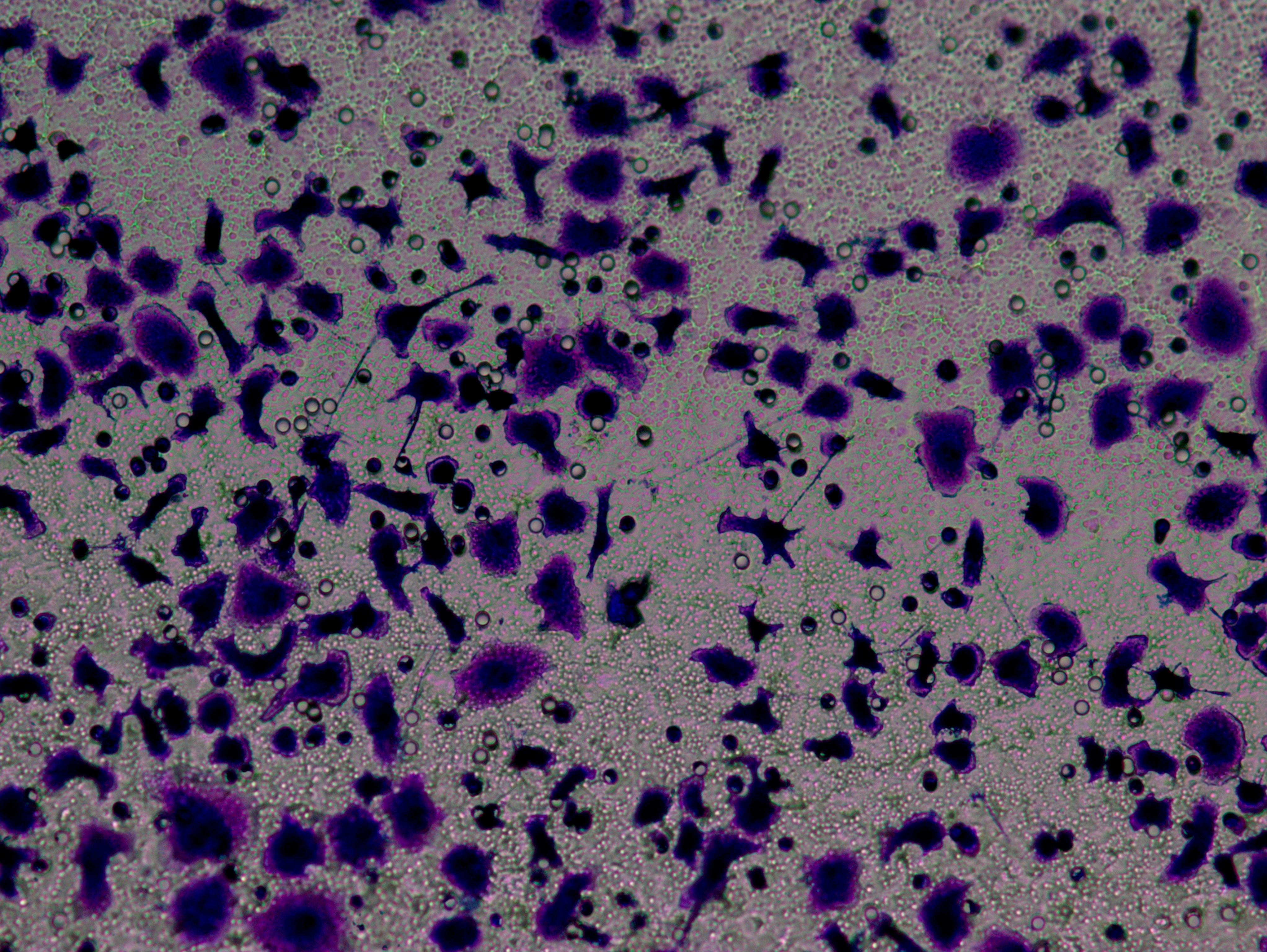

Supplement: Supplemental Information 8 [file peerj-11-15373-s008.zip › Figure 5C-D-images200X-SMMC-7721/shFBXO43.jpg]

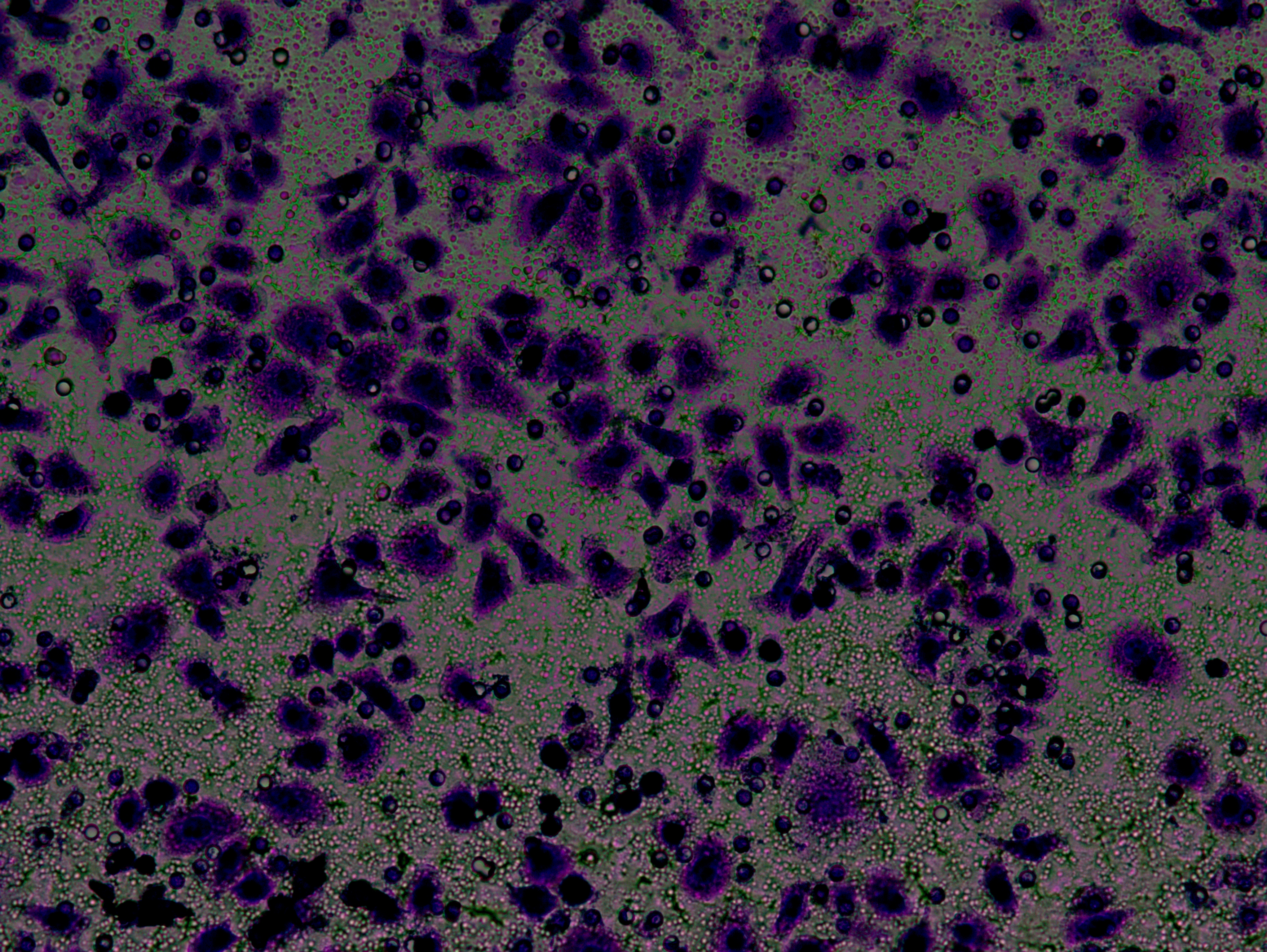

Supplement: Supplemental Information 9 [file peerj-11-15373-s009.zip › Figure 5C-D-images200X-BEL-7404/shCtrl.jpg]

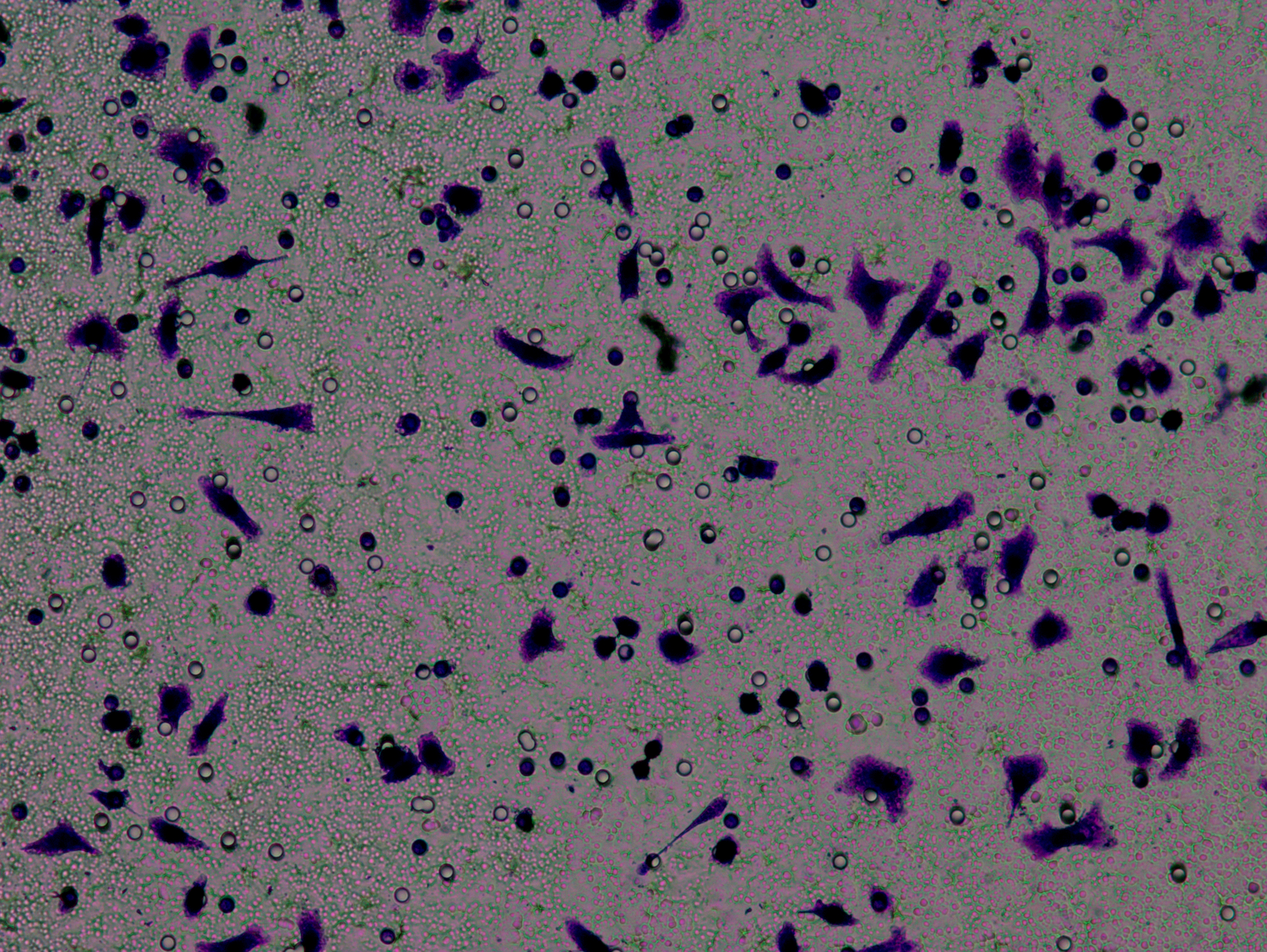

Supplement: Supplemental Information 9 [file peerj-11-15373-s009.zip › Figure 5C-D-images200X-BEL-7404/shFBXO43.jpg]
